# Ceramic-Processing Perspectives on Colloidal CIGS and CZTSSe Thin-Film Solar Absorbers: Green-Body Formation, Reactive Chalcogenization, and Defect Engineering

**DOI:** 10.3390/ma19142989

**Published:** 2026-07-10

**Authors:** Hsing-I. Hsiang

**Affiliations:** Department of Resources Engineering, National Cheng Kung University, Tainan 70101, Taiwan; hsingi@mail.ncku.edu.tw

**Keywords:** CIGS, CZTSSe, colloidal processing, constrained sintering, reactive chalcogenization, ligand exchange, secondary phases, defect chemistry, photovoltaic devices

## Abstract

**Highlights:**

- Colloidal CIGS/CZTSSe films are treated as constrained particulate green bodies.
- Ligand removal, packing, drying, burnout and chalcogenization control absorber quality.
- Densification alone is insufficient; defects and interfaces govern CZTSSe device losses.
- Ceramic-processing concepts are linked to photovoltaic loss mechanisms and diagnostics.

**Abstract:**

Colloidal processing provides a scalable non-vacuum route for fabricating CIGS and CZTSSe thin-film absorbers, but nanoparticle-derived films should be treated as constrained particulate green bodies rather than as simple chemically deposited semiconductor layers. This review reorganizes colloidal chalcogenide photovoltaics using ceramic-processing concepts: ink dispersion, green-body packing, capillary drying stress, ligand burnout, constrained shrinkage, reactive chalcogenization, transient liquid-assisted coarsening, secondary-phase control, defect chemistry, and interface reactions. The central argument is that film densification and grain growth are necessary but insufficient for high-performance CZTSSe devices. Residual carbon, Sn loss, Cu/Zn disorder, ZnSe or Cu_2−x_Se secondary phases, excessive MoSe_2_, and nonideal absorber/buffer band alignment can dominate open-circuit-voltage loss, fill factor, and carrier collection even when the absorber appears dense in cross-sectional microscopy. By linking ceramic-processing variables to photovoltaic loss mechanisms, this review identifies practical routes for improving colloidal chalcogenide solar cells: controlled ligand exchange and binder burnout, high-green-density precursor design, moderated chalcogen chemical potential, transient liquid management, depth-resolved phase analysis, and integrated front/back-interface engineering.

## 1. Introduction

### 1.1. Motivation

The rapid expansion of renewable energy is no longer only an environmental preference; it is an urgent technological requirement for decarbonizing electricity generation, transportation, buildings, and industrial heat. Photovoltaics are central to this transition because they can be deployed from distributed rooftops to utility-scale plants, but no single photovoltaic technology satisfies all requirements for efficiency, cost, material availability, toxicity, stability, and manufacturability. Crystalline Si dominates the present market because of its high efficiency, reliability, and mature manufacturing base, whereas perovskite and perovskite/Si tandem solar cells offer very high performance but still face stability, scale-up, and lead-management concerns [1,2]. CdTe is commercially mature and scalable, but it is constrained by Cd toxicity perception and Te availability. III-V absorbers and III-V nanowire architectures offer exceptional optoelectronic quality, strong light trapping, and prospects for advanced multi-junction or intermediate-band concepts, but their cost, epitaxial complexity, and nanowire-device integration remain major challenges [3,4]. Within this broader landscape, CIGS and CZTSSe are important because they combine direct band gaps, high optical absorption, thin absorber requirements, and compatibility with flexible or lightweight modules while also exposing distinct materials challenges that motivate the present review.

**Figure 1:**
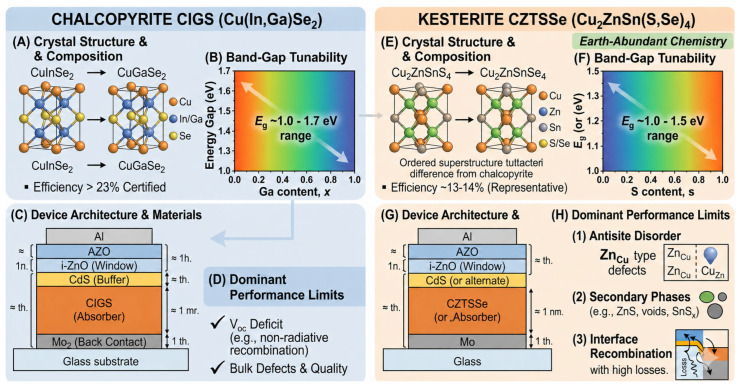
Schematic comparison of chalcopyrite CIGS and kesterite CZTSSe thin-film solar cells, highlighting absorber composition, crystal-structure relationship, band-gap tunability, representative device architecture, and dominant performance-limiting factors. CIGS has reached certified efficiencies above 23%, whereas CZTSSe offers earth-abundant chemistry but remains limited by antisite disorder, secondary phases, and interface recombination. AI-assisted schematic; scientific basis supported by Refs. [1,5,6,7,8,9].

Against this background, chalcopyrite Cu(In,Ga)Se_2_ (CIGS) and kesterite Cu_2_ZnSn(S,Se)_4_ (CZTSSe) occupy a distinctive position among high-efficiency thin-film absorbers. CIGS is one of the most successful compound thin-film photovoltaic materials; recent certified CIGS-family devices have reached 23.64% efficiency through Ag alloying and steep back-contact Ga grading [1,5]. CZTSSe has lower demonstrated efficiency than CIGS, CdTe, perovskite, or III-V devices, but it replaces In and Ga with more earth-abundant Zn and Sn and therefore remains scientifically and technologically important as a sustainable kesterite absorber platform [6,7,8,9]. The comparison with competing photovoltaic systems reinforces the focus of this review: for CIGS and especially CZTSSe, the central issue is not only achieving high optical absorption but also understanding how precursor processing, chalcogenization, defects, secondary phases, and interfaces determine the gap between material potential and device performance. The structural and device-level contrast is therefore introduced schematically in Figure 1.

Conventional high-efficiency CIGS and CZTSSe devices are commonly fabricated by vacuum-based routes such as co-evaporation, sputtering followed by selenization, or sequential elemental deposition. These methods provide strong control over film composition, crystallinity, and absorber/buffer interfaces, but they require high-vacuum equipment, relatively high capital investment, and precise process control over large areas [1,5,9].

Unlike previous reviews that mainly emphasize photovoltaic performance, kesterite defect chemistry, or solution chemistry, this review reorganizes colloidal CIGS/CZTSSe processing using ceramic-processing concepts: green-body packing, binder burnout, capillary-stress-driven cracking, constrained shrinkage, reactive chalcogenization, transient liquid-assisted coarsening, and interface-reaction control. The objective is not to relabel photovoltaic processing with ceramic terminology, but to use ceramic science to identify the processing variables that determine film continuity, densification, secondary-phase distribution, defect formation, and device loss.

**Figure 2:**
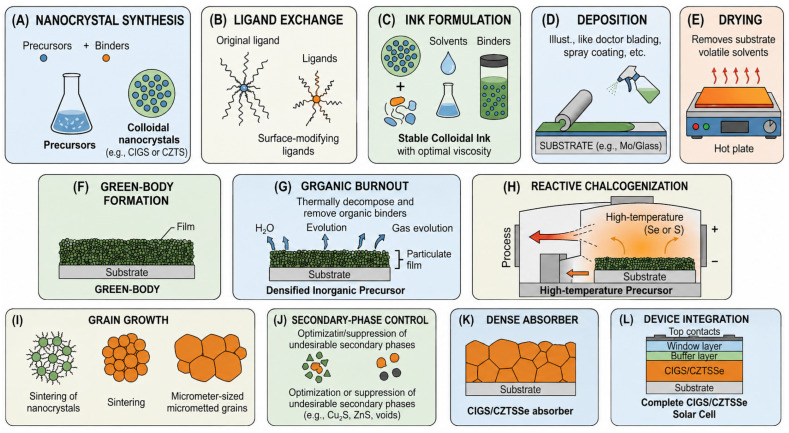
Ceramic-processing framework for colloidal CIGS/CZTSSe absorbers. The sequence links nanocrystal synthesis, ligand exchange, ink formulation, deposition, drying, green-body formation, organic burnout, reactive chalcogenization, grain growth, secondary-phase control, and device integration. AI-assisted schematic; scientific basis supported by Refs. [10,11,12,13,14,15,16,17].

Among the various non-vacuum strategies, colloidal processing is especially attractive because it decouples precursor synthesis from film formation. In this approach, CIGS, CZTS, CZTSe, or CZTSSe nanocrystals are first synthesized with controlled composition, particle size, and surface chemistry, then dispersed into printable inks for subsequent deposition and thermal conversion [10,11,12,13,14,15]. From a ceramic-processing perspective, this route is analogous to the fabrication of a thin ceramic green body, but the analogy has a defined scope: particle packing, dispersion stability, solvent evaporation, binder or ligand removal, and constrained shrinkage are ceramic-style precursor phenomena, whereas S/Se exchange, Sn volatility, chalcogen chemical potential, secondary-phase redistribution, and MoSe_2_ formation are chalcogenide-specific chemical processes. The ceramic sequence is therefore used here as an organizing framework for precursor-state control, not as a claim that colloidal chalcogenide absorber formation is directly equivalent to oxide-ceramic sintering (Figure 2).

However, the conversion of a nanoparticle compact into a dense, large-grained semiconductor absorber remains challenging. Long-chain organic ligands such as oleylamine are often required to stabilize nanocrystals in solution, but incomplete ligand removal can leave residual carbon at particle contacts and grain boundaries, suppressing mass transport and increasing carrier recombination [10,13,14,15]. During drying and heat treatment, the film is constrained by the rigid Mo-coated glass substrate, preventing free in-plane shrinkage and generating tensile stresses that promote mud cracking, delamination, and residual porosity [16,17].

The motivation of this review is therefore to reinterpret colloidal CIGS and CZTSSe thin-film fabrication through a ceramic-processing lens. Rather than treating the absorber layer simply as a chemically deposited semiconductor film, this review emphasizes the absorber precursor as a constrained particulate compact whose final photovoltaic performance is governed by the coupled evolution of packing density, organic residues, grain growth, secondary phases, point defects, and interfaces. This perspective provides a unified framework for understanding why non-vacuum colloidal routes are promising for scalable manufacturing, while also clarifying why their performance still lags behind the best vacuum-processed devices, especially for CZTSSe.

Unlike previous reviews that have primarily summarized device performance, kesterite defect chemistry, or solution-precursor chemistry, this review focuses on a less explicitly addressed but technologically important question: how does the as-deposited nanoparticle compact predetermine the subsequent chalcogenization pathway and, ultimately, the photovoltaic loss mechanisms? This distinction is central to colloidal CIGS and CZTSSe processing because the absorber is not initially formed as a continuous semiconductor layer. It is first deposited as a constrained particulate green body composed of nanocrystals, surface ligands, solvent residues, interparticle pores, particle–particle contacts, drying-induced stresses, and a mechanically rigid Mo-coated substrate. These precursor-state features are established before high-temperature chalcogenization, but they strongly influence whether the final absorber becomes dense and electronically continuous, carbon-contaminated, cracked, nonuniformly converted, or locally enriched with buried secondary phases.

The green-body framework therefore provides more than a ceramic terminology for known processing problems. Its value lies in converting colloidal absorber fabrication into a set of precursor-state criteria that can be measured before chalcogenization and correlated with final device behavior. In this framework, drying defects are interpreted through a shrinkage–constraint criterion, in which green density, film thickness, solvent evaporation rate, particle packing, and substrate adhesion determine the tendency for cracking, delamination, and nonuniform chalcogen transport. Organic removal is treated through a carbon-removal criterion, in which ligand exchange and burnout must be judged by residual carbon at particle contacts, grain boundaries, and interfaces, rather than by improved surface morphology or loss of organic functional groups alone. Chalcogenization is described through a reaction-transport criterion, in which pore connectivity, crack networks, agglomerates, and carbon-blocked contacts control Se/S penetration, phase-conversion uniformity, secondary-phase redistribution, and MoSe_2_ formation. Grain growth is further evaluated through a transient liquid management criterion, which distinguishes beneficial liquid-assisted coarsening from excessive liquid segregation, conductive Cu_2−x_Se formation, Sn-related phase loss, or nonuniform interface reaction. Finally, the framework introduces a microstructure–defect decoupling criterion, emphasizing that densification and grain growth are necessary but not sufficient for CZTSSe because Cu/Zn disorder, Sn-related defects, band tailing, secondary phases, and interface recombination can still dominate the V_OC_ deficit.

This perspective provides predictive insight by shifting attention from final morphology alone to the precursor conditions that bias the entire processing trajectory. It explains why nominally similar compositions and annealing conditions can yield very different absorber quality and device performance: the high-temperature reaction is already constrained by the green-body packing density, organic loading, pore structure, drying history, and Mo/precursor contact established during film formation. The framework also clarifies why a dense or large-grained absorber is not automatically a high-quality photovoltaic absorber, especially in CZTSSe. A film may appear compact in cross-sectional SEM but still contain buried carbon, localized ZnSe or Cu_2−x_Se, excessive MoSe_2_, Sn-related defects, or disorder-induced band tails that degrade V_OC_, FF, EQE, and carrier lifetime. In this sense, the green-body framework complements existing photovoltaic and defect-chemistry reviews by linking colloidal film formation to experimentally testable processing–structure–defect–device relationships for non-vacuum CIGS and CZTSSe solar cells.

Critical summary: The central strength of colloidal CIGS/CZTSSe processing is its compatibility with scalable, non-vacuum manufacturing, but its weakness is that precursor-state defects are easily hidden until they appear as voltage, current-collection, or reproducibility losses. The review therefore treats the green body not as a rhetorical analogy, but as the earliest measurable state from which later device limitations can be anticipated.

#### Organization of This Review

This review is organized to guide readers from processing chemistry to device-level consequences. Section 1 first frames the renewable-energy motivation and the need for scalable thin-film absorbers, then defines the ceramic-processing viewpoint used throughout the article and contrasts the established CIGSSe platform with earth-abundant CZTSSe.

Section 2 examines precursor synthesis and ink formulation. Section 2.1 compares nanocrystal synthesis routes, including hot-injection, heating-up, solvothermal/hydrothermal, and molecular or hybrid precursor approaches (Section 2.1.1, Section 2.1.2, Section 2.1.3 and Section 2.1.4). Section 2.2 and Section 2.3 then connect ligand exchange, carbon removal, ink stability, and green-body packing to the density and chemical homogeneity of the deposited precursor layer.

Section 3 discusses constrained drying, burnout, and pre-sintering. It separately treats drying-induced shrinkage and cracking (Section 3.1), organic burnout and residual-carbon control (Section 3.2), and pressure-assisted pre-sintering or green-body compaction as routes to improved precursor contact and reduced pore volume (Section 3.3).

Section 4 focuses on reactive chalcogenization and microstructural evolution. The discussion distinguishes the ceramic analogy of reactive sintering (Section 4.1) from chalcogenide-specific pressure-assisted selenization (Section 4.2), transient liquid-assisted grain growth (Section 4.3), and bilayer microstructure formation (Section 4.4).

Section 5 connects phase evolution, secondary phases, and defect chemistry. Section 5.1.1 and Section 5.1.2 describe phase-evolution pathways in CIGSSe and CZTSSe, respectively, while Section 5.2 and Section 5.3 discuss how secondary phases and intrinsic defects influence band tailing, recombination, and carrier transport.

Section 6 reviews alloying, surface cleaning, and interface engineering. It covers Ag substitution, Ge alloying, Sb treatment, and Li/alkali doping (Section 6.1.1, Section 6.1.2, Section 6.1.3 and Section 6.1.4), followed by absorber-surface cleaning (Section 6.2) and interface/back-contact control strategies (Section 6.3).

Section 7 translates the processing and microstructural issues into transport and performance metrics. It discusses carrier-transport mechanisms (Section 7.1), device metrics and diagnostic signatures (Section 7.2), and the remaining bottlenecks that limit voltage, fill factor, and carrier collection (Section 7.3).

Section 8 outlines future directions, including operando characterization of reactive chalcogenization (Section 8.1), interface passivation and contact selectivity (Section 8.2), alloying and defect control linked to the V_OC_ deficit (Section 8.3), and scalable, environmentally safer manufacturing (Section 8.4). Section 9 closes with the principal conclusions and the processing–structure–property criteria that emerge from the review.

### 1.2. Ceramic-Processing Viewpoint: From Descriptive Analogy to Predictive Criteria

Nanoparticle-derived CIGS and CZTSSe absorber layers should not be viewed only as chemically deposited semiconductor films. In colloidal processing, the deposited layer first exists as a constrained particulate precursor compact composed of nanocrystals, surface ligands, solvent residues, interparticle pores, particle–particle contacts, drying-induced stresses, and a mechanically rigid Mo-coated substrate. In this review, the term “green body” is used in a restricted precursor-state sense: it denotes the as-deposited or partially dried particulate film before full sulfurization or selenization. The term does not imply a free-standing ceramic compact, conventional powder pressing, or direct equivalence to bulk ceramic green-body compaction. Its usefulness is that green density, pore connectivity, organic loading, substrate adhesion, and drying stress can bias later chalcogenization, secondary-phase redistribution, interface reaction, defect formation, and photovoltaic loss [15,16,17,18,19,20,21,22].

To avoid overextending ceramic terminology, this review separates three levels of discussion. First, ceramic concepts such as green-body formation, constrained shrinkage, burnout, and consolidation are used only to describe measurable precursor-state variables. Second, chalcogenide-specific chemistry describes the actual absorber-forming reactions, including Se/S vapor transport, S/Se exchange, Sn-containing volatile species, kesterite or chalcopyrite phase formation, transient chalcogenide liquids, and MoSe_2_ growth. Third, a mechanism is described as directly demonstrated only when supported by appropriate evidence, such as density or thickness change for compaction, quenched or in situ phase analysis for transient liquids, Raman/XPS/SIMS/TEM evidence for secondary phases, and device diagnostics for recombination or carrier-collection losses [7,8,9,16,17,23,24,25,26,27,28,29,30,31,32,33].

**Figure 3:**
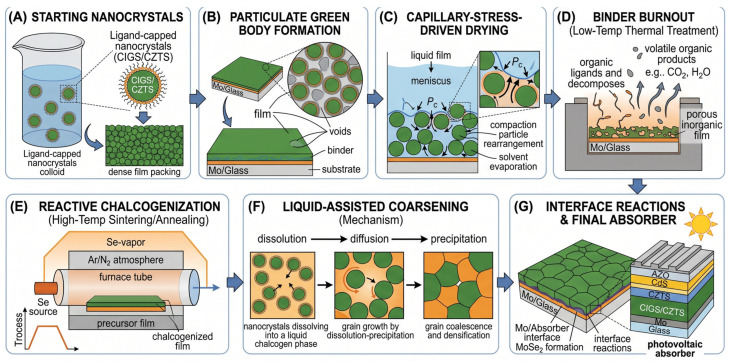
Ceramic-processing view of a nanoparticle-derived chalcogenide absorber. Ligand-capped nanocrystals form a constrained particulate green body that undergoes capillary-stress-driven drying, binder burnout, reactive chalcogenization, liquid-assisted coarsening, and interface reactions before becoming a photovoltaic absorber. AI-assisted schematic; scientific basis supported by Refs. [15,16,17,20,23,24,32,34].

This concept is illustrated schematically in Figure 3. Ligand-capped nanocrystals are first assembled into a particulate film on a Mo-coated glass substrate. During solvent evaporation, capillary forces pull the particles together, while the rigid substrate restricts in-plane shrinkage and generates tensile stress. During organic burnout, residual ligands and carbonaceous species may either be removed cleanly or remain at particle contacts and grain boundaries. During reactive chalcogenization, Se/S vapor transport, S/Se exchange, phase transformation, possible transient liquid formation, grain coarsening, secondary-phase redistribution, and Mo back-contact reactions can occur simultaneously. Thus, Figure 3 is not intended to claim a single universal sintering mechanism. It defines the central hypothesis of this review: precursor-state physical variables and chalcogenide-specific reaction chemistry jointly predetermine absorber quality [16,17,20,23,24,32,34].

This viewpoint differs from previous reviews that primarily emphasize final device performance, intrinsic kesterite defect chemistry, or solution precursor chemistry. Those perspectives are essential, but they often begin with the final absorber or final device response. The ceramic-processing framework instead asks which precursor-state variables can be measured before chalcogenization and how those variables determine the probability of obtaining a dense, chemically uniform, and electronically benign absorber. Its value is therefore not the replacement of photovoltaic or defect-chemistry analysis, but the addition of a processing–structure–defect–device bridge that links the as-deposited compact to the final solar-cell behavior [7,8,9,15,21,28].

The first predictive element is the shrinkage–constraint relationship. During drying, solvent evaporation produces capillary pressure within the nanoparticle network and drives particle rearrangement and film shrinkage. Because the film is attached to a rigid Mo-coated glass substrate, in-plane shrinkage is restricted. The resulting tensile stress depends on green density, film thickness, particle size distribution, solvent evaporation rate, organic fraction, and adhesion to the substrate. This relationship predicts that cracking, delamination, and thickness nonuniformity are not merely post-deposition defects, but consequences of an imbalance between the shrinkage required by the particle compact and the shrinkage permitted by the substrate. From this viewpoint, thinner sequential coatings, higher green density, controlled drying rate, reduced unnecessary organic loading, and improved wetting on Mo are not empirical adjustments; they are routes to reduce constrained-shrinkage stress and to improve the uniformity of subsequent Se/S transport [16,17,18,20,21].

The second predictive element is the carbon-removal relationship. Long-chain ligands such as oleylamine are beneficial for nanocrystal synthesis and ink stability, but they can become detrimental during absorber formation if they are not removed or exchanged effectively. Residual carbon at particle contacts, grain boundaries, or the Mo/absorber interface can suppress interparticle diffusion, inhibit liquid-assisted coarsening, increase series resistance, and create recombination-active regions. Therefore, ligand removal should not be judged only by smoother film morphology or by the apparent disappearance of organic bands in FTIR. A green-body-based assessment requires correlating TGA/DSC, FTIR, Raman carbon signals, XPS, SIMS depth profiles, and, where possible, TEM/EELS with microstructure and device response. This criterion explains why a film can appear dense in cross-sectional SEM but still show poor carrier collection or low fill factor if carbon-rich barriers remain buried within the absorber [15,21,22,34].

The third predictive element is the reaction-transport relationship. Selenization or sulfurization of a nanoparticle compact is a coupled process involving chalcogen vapor transport, S/Se exchange, phase transformation, cation redistribution, grain coarsening, secondary-phase redistribution, and Mo back-contact reaction. The efficiency and uniformity of this process are controlled by the precursor pore network, crack distribution, agglomeration state, and cleanliness of particle contacts. A homogeneous green body with connected but not excessive porosity can promote uniform Se/S penetration and reaction-front propagation. In contrast, dense surface skins, large agglomerates, drying cracks, or carbon-blocked contacts can produce nonuniform chalcogenization, fine-grained bottom layers, buried ZnSe or Sn-related phases, conductive Cu_2−x_Se regions, or excessive MoSe_2_ growth. Thus, Raman mapping, SIMS depth profiling, EDS mapping, cross-sectional SEM/TEM, and EQE analysis should be interpreted together, because they reflect different consequences of the same precursor-controlled reaction pathway [21,23,24,25,26,27,28,29,30,31,32,35].

The fourth predictive element is the transient liquid management relationship. In this review, “transient liquid-assisted coarsening” means a liquid or liquid-like chalcogenide phase that exists only during part of the thermal treatment, enhances mass transport by dissolution–reprecipitation or liquid-phase redistribution, and is then consumed or incorporated without leaving a harmful residue. This mechanism should be separated from simple grain growth, volatile-assisted Sn or Se transport, and residual secondary-phase segregation. In CZTSSe, a beneficial transient liquid can promote particle coalescence, grain growth, and pore elimination, but a poorly controlled liquid-derived pathway can also promote Cu-, Sn-, or Se-rich segregation, conductive Cu_2−x_Se formation, Sn loss, ZnSe accumulation, or nonuniform back-contact reaction. Therefore, large grains should not be regarded automatically as evidence of beneficial liquid-assisted coarsening. The mechanism requires supporting evidence for liquid composition, location, lifetime, consumption, and final device effect [23,24,32,35].

The fifth predictive element is the microstructure–defect decoupling relationship. Ceramic-style processing can reduce extrinsic defects such as cracks, residual pores, carbon-contaminated particle contacts, incomplete coalescence, and nonuniform chalcogenization. However, these improvements do not by themselves eliminate the intrinsic electronic penalties of the kesterite lattice. In CZTSSe, Cu/Zn disorder, Sn-related defects, band tailing, secondary phases, and front/back-interface recombination can still dominate the VOC deficit even when the absorber appears dense and large-grained. Therefore, the green-body framework must be coupled with defect-sensitive and device-physics measurements, including PL, TRPL, temperature-dependent J–V, admittance spectroscopy, EQE, Hall measurements, Raman disorder analysis, and interface-sensitive characterization. This prevents the overinterpretation of microstructural densification as proof of electronic quality [7,8,9,28,36,37,38,39].

Taken together, the ceramic-processing viewpoint provides a predictive framework for colloidal CIGS and CZTSSe absorbers by shifting attention from final morphology alone to the precursor conditions that define the later processing trajectory. As summarized conceptually in Figure 3, the high-temperature reaction is already biased by green-body packing density, organic residue, pore connectivity, drying stress, and Mo/precursor contact. This framework explains why nominally similar compositions, annealing temperatures, and chalcogenization atmospheres can yield very different device outcomes. It also clarifies why colloidal CZTSSe optimization cannot rely solely on achieving dense films or large grains. Instead, effective processing must simultaneously control green-body formation, organic removal, reaction transport, transient liquid behavior, secondary-phase localization, and interface reactions, while independently verifying that intrinsic and interfacial recombination losses have been reduced [7,8,9,16,17,21,23,24,32,34].

Critical summary: The ceramic-processing viewpoint is useful because it converts drying, burnout, packing, and constrained shrinkage into measurable precursor variables. Its limitation is that ceramic terminology alone cannot explain chalcogenide-specific chemistry; reliable interpretation requires coupling green-body metrics with Se/S transport, Sn volatility, secondary-phase evolution, and recombination-sensitive device diagnostics [7,8,9,16,17,21,23,24,28,32,34].

### 1.3. CIGS Versus CZTSSe

**Figure 4:**
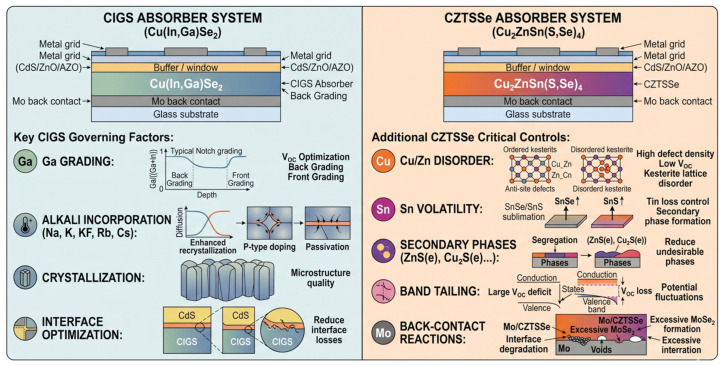
Comparison of CIGS and CZTSSe absorber systems. CIGS is governed mainly by Ga grading, alkali incorporation, crystallization, and interface optimization, whereas CZTSSe additionally requires stringent control of Cu/Zn disorder, Sn volatility, secondary phases, band tailing, and back-contact reactions. AI-assisted schematic; scientific basis supported by Refs. [5,6,7,8,9,23,24,28,29,30,31,32,33,34,36,37,38,39].

Although CIGS and CZTSSe are both multinary chalcogenide thin-film absorbers, they should not be treated as equivalent material systems. Their structural chemistry, defect tolerance, phase stability, and processing limitations are fundamentally different. These material-specific differences are summarized schematically in Figure 4.

The relatively high performance of CIGS arises partly from its ability to tolerate a range of intrinsic defects without forming extremely detrimental recombination centers. In CIGS, Cu-poor growth conditions promote benign defect complexes and help produce favorable electronic properties, while Ga incorporation allows band-gap tuning through the Ga/(In + Ga) ratio [1,5,9].

CZTSSe, by contrast, has a narrower phase-stability window and a more complex defect landscape. The similar ionic sizes of Cu^+^ and Zn^2+^ promote Cu/Zn antisite disorder, leading to band tailing, potential fluctuations, and a large open-circuit-voltage deficit [7,8,9]. This distinction should be interpreted as a fundamental materials difference rather than solely as a processing difficulty. In CIGS, microstructural improvement, alkali incorporation, and band-gap grading can often be translated efficiently into higher device performance because the chalcopyrite lattice is relatively defect-tolerant. In CZTSSe, however, the kesterite lattice intrinsically favors Cu/Zn site disorder because Cu^+^ and ${Zn}^{2+}$ can occupy closely related crystallographic sites with relatively low energetic penalty. As a result, even a dense, large-grained, and apparently phase-pure CZTSSe absorber may retain substantial band tailing and potential fluctuations. This is why the V_OC_ deficit cannot be treated as an afterthought to ceramic densification; it must be considered a central defect-thermodynamic limitation of the kesterite system.

The key distinction is therefore that CIGS processing is primarily a problem of compositional grading, alkali control, crystallization, and interface optimization, whereas CZTSSe processing is additionally constrained by intrinsic cation disorder, narrow phase stability, Sn volatility, secondary-phase formation, and severe V_OC_ loss. This difference is especially important for colloidal processing. The key differences are condensed in Table 1. The processing implications of these differences are mapped to device signatures in Table 2.

Critical summary: CIGS and CZTSSe share related thin-film device architectures, but they should not be optimized using the same assumptions. CIGS benefits from greater defect tolerance and mature alkali/interface control, whereas CZTSSe remains limited by a narrow phase window, cation disorder, Sn-related defects, and a larger VOC deficit; this difference is the main reason that microstructural improvement alone is insufficient for CZTSSe.

## 2. Precursor Synthesis and Ink Formulation

### 2.1. Nanocrystal Synthesis Routes

**Figure 5:**
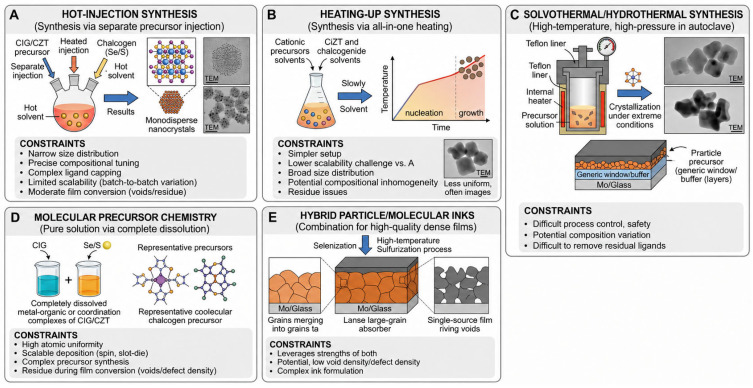
Representative precursor routes for colloidal and solution-processed CIGS/CZTSSe absorbers: hot-injection synthesis, heating-up synthesis, solvothermal/hydrothermal synthesis, molecular precursor chemistry, and hybrid particle/molecular inks. Each route imposes different constraints on particle size, composition, ligand chemistry, scalability, and film conversion. AI-assisted schematic; scientific basis supported by Refs. [10,11,12,13,14,15,40,41,42,43,44,45,46,47,48].

The synthesis route used to prepare CIGS, CZTS, CZTSe, or CZTSSe nanocrystals strongly determines the subsequent ink stability, green-body packing, chalcogenization behavior, and final absorber microstructure. Unlike vacuum-deposited absorbers, where composition and crystallization are controlled during film growth, colloidal processing separates the formation of the precursor particles from the formation of the film. The main precursor routes and their processing implications are compared in Figure 5.

Critical summary: Precursor synthesis is the first point at which particle size, composition, ligand shell, and phase purity can be controlled. The strength of nanocrystal routes is modularity, but their weakness is that a well-dispersed ink does not guarantee a chemically uniform, low-carbon, and electronically benign absorber after chalcogenization.

#### 2.1.1. Hot-Injection Synthesis

The hot-injection method is one of the most widely used approaches for preparing monodisperse semiconductor nanocrystals. In this route, a reactive precursor, often containing the chalcogen source, is rapidly injected into a hot coordinating solvent containing metal precursors. The sudden increase in supersaturation promotes burst nucleation, followed by a separated growth stage, which can yield relatively uniform particles with good crystallinity [40,41]. For binary and some ternary chalcogenides, this method provides excellent control over particle size, shape, and crystallinity.

However, the extension of hot-injection synthesis to multinary CIGS and CZTSSe systems is more difficult. The formation of quaternary or pentanary compounds requires simultaneous or sequential incorporation of several cations with different coordination environments, precursor reactivities, and reaction kinetics. For example, in CIGS nanocrystal synthesis, Cu-, In-, and Ga-containing complexes do not necessarily react at the same rate, which can lead to local compositional inhomogeneity, phase segregation, or incomplete Ga incorporation [10,40].

Critical summary: Hot injection offers strong control of nucleation and particle uniformity, making it attractive for mechanistic studies. Its weakness is limited scalability and sensitivity to injection, temperature, and ligand conditions; therefore, hot-injection success should be judged by absorber density, residual carbon, composition retention, and device statistics, not only by nanocrystal monodispersity.

#### 2.1.2. Heating-Up Synthesis

The heating-up, or non-injection, method was developed to improve scalability and compositional control in multinary chalcogenide nanocrystal synthesis. In this approach, all metal and chalcogen precursors are mixed at relatively low temperature and then heated gradually to the reaction temperature.

The heating-up method is especially attractive for colloidal processing because it is more compatible with larger-batch synthesis and can produce nanocrystals with controlled morphology for ink preparation. However, its success remains highly sensitive to ligand chemistry, solvent composition, heating rate, precursor concentration, and reaction time [10,11].

**Figure 6:**
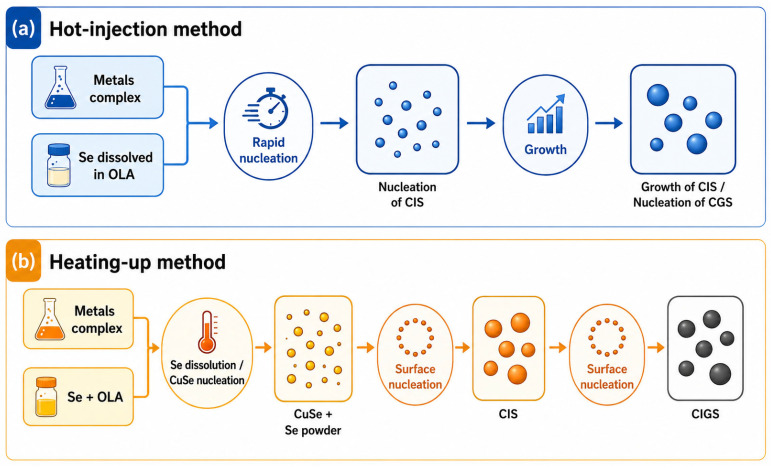
Schematic comparison of nanocrystal formation mechanisms in the hot-injection and heating-up methods. (**a**) In the hot-injection route, rapid mixing of a Se-containing precursor with metal–ligand complexes generates abrupt supersaturation, leading to CIS nucleation followed by particle growth and possible CGS/CIGS formation. (**b**) In the heating-up route, metal complexes, Se, and oleylamine are heated together, so Se dissolution and Cu–Se intermediate formation occur before surface-mediated nucleation and conversion toward CIS and CIGS. The figure highlights the contrast between rapid burst nucleation in hot injection and gradual intermediate-mediated growth in heating-up synthesis [10]. AI-assisted schematic; scientific basis supported by Refs. [10,11,14,40].

The formation mechanism of multinary chalcogenide nanocrystals is strongly governed by the way supersaturation is created during synthesis, as schematically illustrated in Figure 6 [10]. In the hot-injection route (Figure 6a), rapid injection of the Se precursor into a hot metal–ligand solution produces a sudden increase in supersaturation, which promotes fast nucleation followed by particle growth. Ideally, this temporal separation between nucleation and growth enables better control of particle size and crystallinity. However, for multication systems such as CIGS, the different reactivities and coordination strengths of Cu, In, Ga, and Se precursors can lead to nonuniform cation incorporation, delayed Ga participation, or the formation of compositionally heterogeneous particles. In contrast, the heating-up route (Figure 6b) begins with all precursors present in the reaction medium and relies on gradual temperature increase to drive Se dissolution, intermediate phase formation, and subsequent surface-mediated conversion toward CIS or CIGS. This pathway is generally more compatible with scalable synthesis and compositional tuning because nucleation and growth proceed under a continuously evolving chemical environment. Nevertheless, the gradual reaction sequence can also broaden the particle-size distribution and promote intermediate Cu–Se or other secondary phases if precursor reactivity, ligand chemistry, heating rate, and reaction time are not carefully optimized. Therefore, the comparison in Figure 6 highlights that hot injection mainly relies on rapid supersaturation and kinetically separated nucleation/growth, whereas heating-up synthesis proceeds through a more gradual, intermediate-mediated pathway. These differences are important because the resulting particle size, surface chemistry, composition homogeneity, and residual ligand content directly affect ink stability, green-body packing, and subsequent reactive chalcogenization.

Critical summary: Heating-up synthesis is simpler and more scalable than hot injection, but the broader nucleation window can broaden particle-size and composition distributions. Its practical value depends on whether the resulting particles pack, exchange ligands, and chalcogenize reproducibly over large areas.

#### 2.1.3. Solvothermal and Hydrothermal Synthesis

**Figure 7:**
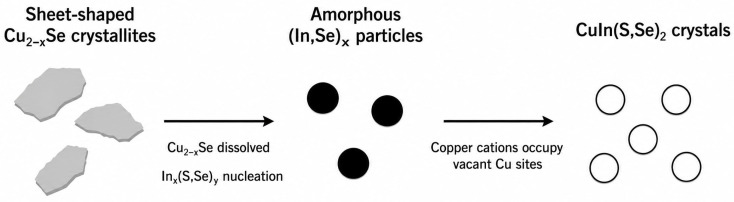
Schematic illustration of the proposed transformation pathway from Cu_2−x_Se and In–Se intermediates to CuIn(S,Se)_2_ crystals. Sheet-shaped Cu_2−x_Se crystallites dissolve during reaction, while amorphous In_x_(S,Se)ᵧ particles nucleate and provide an In–chalcogen framework. Subsequent incorporation of Cu cations into vacant Cu sites promotes the formation and crystallization of CuIn(S,Se)_2_ [12]. AI-assisted schematic; scientific basis supported by Refs. [12,14].

Solvothermal and hydrothermal methods provide relatively simple and low-cost routes for preparing chalcogenide powders or nanocrystals in a closed reaction environment. These methods use elevated temperature and autogenous pressure to promote crystallization from solution, often with less demanding equipment than high-temperature organometallic synthesis. In some cases, chelating agents or sulfur/selenium sources can mediate cation exchange or stepwise crystallization, enabling the formation of ternary or quaternary chalcogenide phases [12]. The schematic in Figure 7 illustrates a possible intermediate-mediated pathway for CuIn(S,Se)_2_ formation during chalcogenide nanocrystal synthesis. In the initial stage, sheet-shaped Cu_2−x_Se crystallites act as a Cu-rich transient phase and can partially dissolve under the reaction conditions. At the same time, amorphous In_x_(S,Se)ᵧ particles nucleate from the In–chalcogen precursor species. The transformation toward CuIn(S,Se)_2_ then proceeds through the incorporation and redistribution of Cu cations into the In–S/Se framework, filling vacant Cu sites and promoting crystallization of the chalcopyrite phase. This pathway emphasizes that the formation of CuIn(S,Se)_2_ is not necessarily a single-step nucleation event, but may involve dissolution of Cu_2−x_Se, formation of amorphous In–chalcogen intermediates, cation redistribution, and final crystal ordering. Such intermediate phases are important because they influence particle morphology, composition homogeneity, and the subsequent conversion behavior of the precursor film during selenization.

The main advantage of solvothermal synthesis is its simplicity and potential scalability. It can also reduce the use of expensive or highly air-sensitive organometallic precursors.

Critical summary: Solvothermal and hydrothermal routes can produce crystalline particles under relatively accessible conditions, but compositional homogeneity and ligand/solvent removal remain critical weaknesses. These routes are most convincing when particle chemistry is linked to film packing, phase evolution, and final device response.

#### 2.1.4. Molecular and Hybrid Precursor Routes

Molecular precursor routes differ from particle-based colloidal routes in that the absorber film is formed from dissolved metal–chalcogen complexes, salts, or molecular species rather than preformed crystalline nanoparticles. This approach can provide excellent compositional mixing at the molecular scale and has produced high-performance solution-processed chalcogenide devices, including hydrazine-derived CZTSSe and CIGSSe absorbers [6,15,36,42].

Hybrid precursor routes combine the advantages of nanoparticles and molecular precursors. In these systems, crystalline nanoparticles can provide structural or compositional building blocks, while molecular species improve interparticle contact, fill pores, promote densification, or compensate local composition during chalcogenization [15,43,44]. The advantages, limitations, and processing relevance of the major precursor routes are compared in Table 3.

Critical summary: Molecular and hybrid routes can improve atomic-level mixing and reduce some particle-packing limitations, but they can introduce different risks, including residual organics, complex decomposition pathways, and rapid phase segregation. Their advantage is strongest when dense film formation is paired with controlled chemistry and verified defect reduction.

### 2.2. Ligand Exchange and Carbon Control

**Figure 8:**
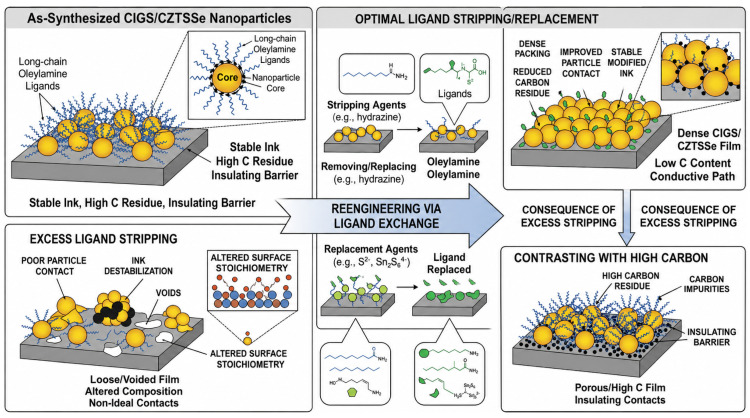
Ligand exchange and carbon-residue control in nanoparticle-derived CIGS/CZTSSe films. Replacing or removing long-chain oleylamine ligands can improve particle contact and reduce carbon residues, but excessive ligand stripping may destabilize inks or alter surface stoichiometry. AI-assisted schematic; scientific basis supported by Refs. [15,21,34,40,41].

Surface ligands are indispensable for colloidal nanocrystal synthesis, but they also represent one of the most serious barriers to the conversion of nanoparticle inks into dense and electronically continuous CIGS or CZTSSe absorber layers. Long-chain coordinating molecules, especially oleylamine (OLA), are commonly used during hot-injection or heating-up synthesis because they dissolve metal precursors, control nucleation and growth, prevent particle agglomeration, and stabilize the nanocrystals in nonpolar solvents [10,11,40,41]. The ligand-removal problem and its processing consequences are summarized in Figure 8.

For CIGS nanocrystals prepared by the heating-up route, Yang and Hsiang showed that OLA adsorbed on CuIn_0.7_Ga_0.3_Se_2_ particle surfaces is difficult to remove by simple heat treatment. They reported that ligand exchange or solvent treatment with 1-hexanethiol and m-xylene promotes removal of OLA-derived organic species, suppresses residual carbon formation during sintering, and improves densification and grain growth of the CIGS film after selenization [34]. This result is important from a ceramic-processing viewpoint because it demonstrates that the surface chemistry of the precursor particles controls not only the dispersion state of the ink, but also the mass transport and densification behavior during subsequent thermal treatment.

The role of 1-hexanethiol is particularly important because it can replace long-chain OLA with a shorter sulfur-containing surface species. Compared with OLA, 1-hexanethiol has a shorter hydrocarbon chain and can reduce the organic volume between adjacent nanocrystals. This promotes closer particle packing and decreases the amount of carbon that must be removed during heating.

Solvent washing is another important but conceptually distinct approach. Solvents such as m-xylene can assist in removing weakly bound or excess OLA from the particle surface and reduce the organic fraction in the deposited film [34].

Carbon control is especially critical because residual carbon affects both microstructure and device physics. From a processing perspective, carbonaceous residues at particle contacts suppress interdiffusion, reduce the effectiveness of transient liquid-assisted sintering, and inhibit the formation of large, continuous grains.

The effectiveness of ligand removal should therefore be verified by multiple complementary techniques rather than inferred only from improved film morphology. FTIR can monitor the reduction of C–H stretching bands from long hydrocarbon chains and N–H-related features associated with OLA.

Ligand exchange should therefore be discussed as part of the broader green-body engineering strategy. The objective is not simply to remove organics, but to design a precursor compact that can shrink, react, and coarsen without leaving insulating or recombination-active residues. The processing consequences of ligand chemistry and residual carbon are summarized in Table 4.

Critical summary: Ligand exchange is one of the most powerful levers for improving particle contact and lowering residual carbon. Its weakness is that excessive or incomplete exchange can destabilize the ink, induce aggregation, or leave carbon-rich grain boundaries; therefore, ligand chemistry must be evaluated by XPS/SIMS/Raman, transport, PL/TRPL, and device reproducibility rather than by ink stability alone.

### 2.3. Ink Stability and Green-Body Packing

Ink stability and green-body packing are critical links between nanocrystal synthesis and final absorber performance in colloidal CIGS and CZTSSe solar cells. Even when the precursor particles are phase-pure and compositionally well controlled, poor dispersion, inappropriate viscosity, weak substrate wetting, or nonuniform drying can generate defects before chalcogenization begins [18,21,40,41,45,46,48].

The solid loading of the ink directly affects the packing density and shrinkage behavior of the precursor layer. A low-solid-loading ink may spread easily and produce smooth coatings, but it often forms a highly porous green film after solvent evaporation [18,20,21].

Viscosity is another key parameter because it controls film thickness, leveling behavior, and coating uniformity. For spin coating, relatively low-viscosity inks are often preferred to produce smooth and thin layers, whereas doctor blading, slot-die coating, screen printing, and inkjet printing require different viscosity windows and rheological behavior [18,21,22,45,46].

**Figure 9:**
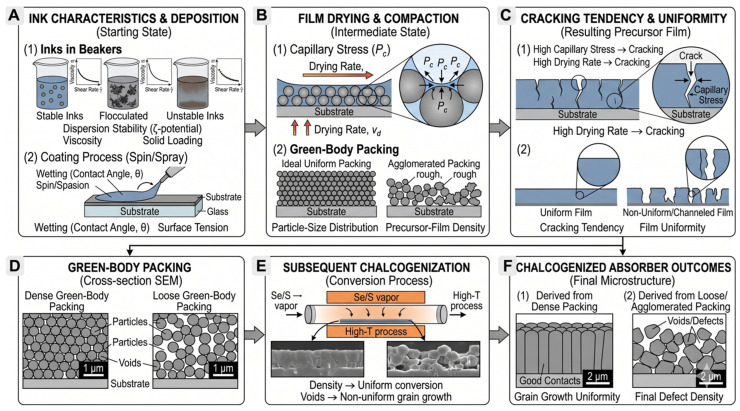
Ink stability and green-body packing factors affecting colloidal absorber formation. Solid loading, viscosity, dispersion stability, wetting, drying rate, and particle-size distribution determine precursor-film density, capillary stress, cracking tendency, and the uniformity of subsequent chalcogenization [18,20,21]. (**A**) Ink characteristics and deposition are governed by dispersion stability, viscosity, solid loading, wetting behavior, contact angle, and surface tension. (**B**) During drying, solvent evaporation generates capillary stress, driving particle rearrangement and compaction into either uniform or agglomerated green-body packing. (**C**) Excessive capillary stress and rapid drying increase cracking tendency and film nonuniformity. (**D**) Dense green-body packing provides closer particle contacts and fewer voids, whereas loose packing introduces porosity and poor structural continuity. (**E**) During high-temperature Se/S chalcogenization, dense and uniform precursors promote more homogeneous conversion, while porous or agglomerated precursors lead to nonuniform reaction and void retention. (**F**) Consequently, dense precursor packing favors uniform grain growth and good contacts, whereas loose or agglomerated packing produces higher defect density and less uniform absorber microstructures. AI-assisted schematic; scientific basis supported by Refs. [18,20,21].

Wetting between the ink and the Mo-coated soda-lime glass substrate is equally important. Good wetting promotes continuous coverage, uniform film thickness, and intimate contact with the back electrode. These ink and green-body variables are grouped schematically in Figure 9 [18,21,34,40,41,45,46].

Drying stress is a major cause of defect formation in nanoparticle-derived absorber layers. During solvent evaporation, capillary pressure pulls particles together and causes the film to shrink [20].

Bimodal particle packing provides one possible strategy to improve green-body integrity. By mixing submicron particles with nanoparticles, the larger particles can form a relatively rigid structural skeleton, while smaller nanoparticles fill the interstitial spaces and improve packing density [18,20].

However, submicron/nanoparticle blending also introduces important risks. Because smaller particles and larger particles may react, dissolve, or recrystallize at different rates during chalcogenization, bimodal mixtures can produce local conversion inhomogeneity [21,32,48].

The quality of the green body can be assessed using both ceramic-processing and photovoltaic-characterization tools. Film thickness and roughness can be measured by profilometry or cross-sectional SEM [18,21,34,40,41].

Drying and constrained shrinkage should be analyzed using explicit ceramic-science variables. The capillary pressure during drying scales with solvent surface tension and inversely with pore radius; therefore, smaller nanoparticles and narrower pores can generate larger drying stresses. The key ink-formulation and green-body-packing variables are organized in Table 5 [18,20].

Critical summary: Ink stability and green-body packing are strengths only when they lead to uniform, crack-free, low-porosity precursor films. A visually smooth coating can still contain unfavorable particle contacts, pore networks, or compositional gradients, so rheology and coating quality must be connected to density, drying stress, chalcogen penetration, and spatially resolved device performance [18,20,21].

## 3. Constrained Drying, Burnout, and Pre-Sintering

The transformation of a colloidal CIGS or CZTSSe ink into a dense photovoltaic absorber begins well before high-temperature chalcogenization. After deposition on Mo-coated soda-lime glass, the precursor layer behaves as a thin constrained particulate compact.

### 3.1. Constrained Shrinkage and Cracking

Nanoparticle-derived CIGS and CZTSSe precursor films are particularly vulnerable to constrained shrinkage because they are deposited as thin particulate green bodies on rigid Mo-coated glass substrates. During solvent evaporation, capillary pressure pulls adjacent particles together and causes the film to shrink.

This constrained-shrinkage problem is more severe in nanoparticle-derived chalcogenide films than in many conventional powder films because of their high surface area, high ligand content, large solvent fraction, and large capillary pressure during drying. Smaller particles provide a high driving force for densification and reactive grain growth, but they also increase the magnitude of drying shrinkage and the amount of organic material that must be removed during burnout [15,18].

The formation of drying cracks is strongly influenced by film thickness, particle packing density, solvent evaporation rate, adhesion to the Mo substrate, and the mechanical strength of the green network. Thicker coatings accumulate larger elastic energy during constrained drying and are therefore more prone to channel cracking.

Constrained shrinkage also affects the later chalcogenization process. Cracks and pores produced during drying act as nonuniform diffusion pathways for Se or S vapor, causing spatially uneven chalcogenization.

From a ceramic-processing perspective, the key strategy is to reduce the mismatch between the shrinkage demanded by the particle compact and the shrinkage allowed by the rigid substrate. This can be achieved by increasing the initial green density, controlling the particle-size distribution, reducing the organic content, slowing the drying rate, improving film adhesion, and using multilayer coating with intermediate drying or mild preheating. The typical constrained-shrinkage defects and mitigation routes are summarized in Table 6.

Critical summary: Constrained-shrinkage analysis clarifies why cracks, pinholes, and delamination arise even when the ink appears well formulated. The limitation is that crack suppression alone does not prove electronic quality; the same processing changes must also be checked for residual porosity, secondary phases, back-contact reactions, and shunt statistics.

### 3.2. Organic Burnout

Organic burnout is a critical intermediate step between green-body formation and reactive chalcogenization in nanoparticle-derived CIGS and CZTSSe films. In colloidal routes, organic species originate from coordinating ligands, solvents, dispersants, residual precursor molecules, and possible polymeric additives used to stabilize the ink and control coating behavior [10,11,15,34,40,41].

**Figure 10:**
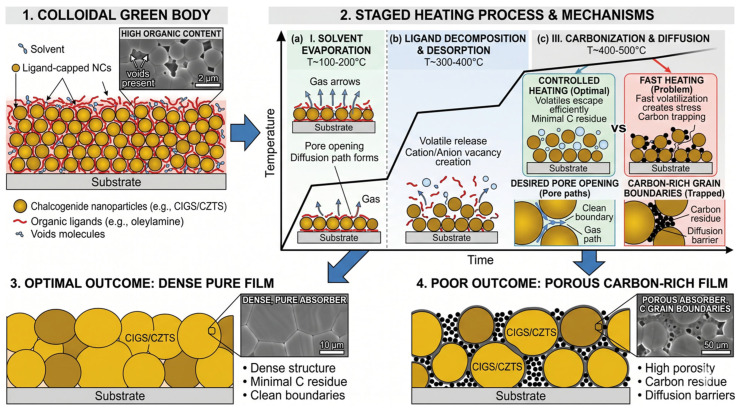
Organic burnout in colloidal chalcogenide green bodies. Solvent evaporation, ligand decomposition, desorption, carbonization, and volatile release must be controlled by staged heating to avoid diffusion barriers, pore opening, and carbon-rich grain boundaries. AI-assisted schematic; scientific basis supported by Refs. [15,21,34,40,41].

The removal of organics from nanoparticle films is more complex than simple solvent evaporation. Long-chain ligands such as oleylamine can be strongly coordinated to nanocrystal surfaces and may decompose over a broad temperature range rather than volatilize cleanly [10,11,34]. The main burnout pathways and associated risks are summarized in Figure 10.

A staged heating schedule is generally preferred because different organic species are removed at different temperatures. The initial low-temperature stage removes residual solvent and weakly adsorbed molecules.

Incomplete burnout strongly affects microstructural evolution during reactive chalcogenization. Carbon-rich residues at particle contacts act as diffusion barriers that suppress interparticle necking, delay liquid-assisted coarsening, and prevent the formation of a dense large-grained absorber [15,34].

The effectiveness of organic burnout should be verified by complementary characterization techniques. TGA and DSC are useful for identifying solvent evaporation, ligand decomposition, oxidation, and possible exothermic carbonization events.

FTIR spectroscopy is useful for monitoring the removal of organic functional groups before and after ligand exchange or burnout. For OLA-capped nanocrystals, the decrease in C–H stretching vibrations from long alkyl chains and N–H-related features provides evidence for ligand removal [34].

Raman spectroscopy is particularly important after thermal treatment because it can detect residual amorphous or graphitic carbon through the characteristic D and G bands. This is essential because a film may show reduced ligand peaks in FTIR but still retain carbonized residues after heating.

XPS provides surface-sensitive information about carbon, nitrogen, sulfur, selenium, and metal chemical states. It can help determine whether residual carbon is present as aliphatic carbon, oxidized carbon, carbon–nitrogen species, or carbon–sulfur-related species.

IMS is valuable for depth profiling residual carbon and other light elements through absorber thickness. This is particularly important for nanoparticle-derived CZTSSe films, where carbon may be nonuniformly distributed and concentrated near the bottom fine-grained layer or back contact. SIMS can also detect Na, K, S, Se, and other elements that influence crystallization and defect chemistry. When combined with cross-sectional SEM, TEM/EELS, Raman mapping, and device characterization, SIMS can help determine whether poor photovoltaic performance arises from bulk carbon residues, interface contamination, or nonuniform chalcogenization.

Evidence for liquid-assisted growth should combine temperature-dependent phase identification with cross-sectional microstructure and composition mapping rather than relying only on grain-size changes. The corresponding characterization methods for burnout and residual carbon are summarized in Table 7.

Critical summary: Burnout control is essential because residual carbon can block coalescence and introduce recombination-active boundaries. The strength of thermal removal is simplicity, but its weakness is that gas evolution and incomplete decomposition can create pores or local composition changes; burnout protocols must therefore be validated by chemical depth profiles and device-level transport metrics.

### 3.3. Pressure-Assisted Pre-Sintering

**Figure 11:**
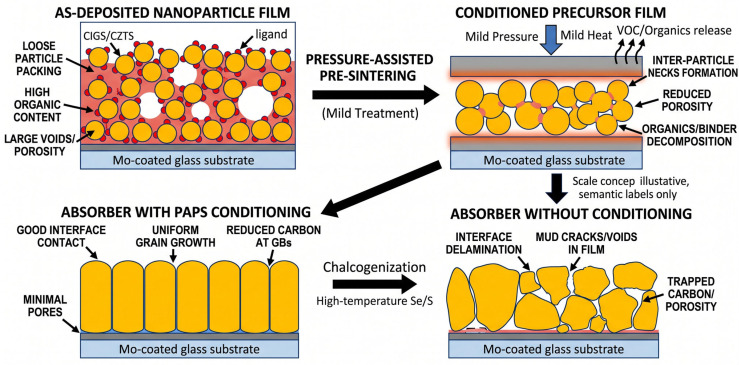
Possible roles of pressure-assisted pre-sintering as a precursor-conditioning step. Mild pressure/thermal treatment may improve particle contact, suppress cracking, reduce porosity, and remove organics. AI-assisted schematic; scientific basis supported by Refs. [15,16,17,18,19,20,21,34].

Pressure-assisted pre-sintering has been reported as a precursor-conditioning strategy for nanoparticle-derived CIGS and CZTSSe thin films, particularly when the deposited precursor layer contains a high organic content, large interparticle porosity, and strong drying-induced constraint from the Mo-coated glass substrate. In this review, this term is used narrowly for a treatment applied before full sulfurization or selenization, in which moderate heat and/or pressure are used to improve the precursor film by increasing particle contact, reducing cracking, partially consolidating the layer, or decreasing residual organic species [15,16,17,18,19,20,21,34]. It is not used here to imply conventional hot pressing, pressureless oxide sintering, or full absorber formation. The proposed roles and limitations of pressure-assisted pre-sintering are summarized in Figure 11.

The first potential benefit of pressure-assisted pre-sintering is the improvement of particle contact. In nanoparticle-derived films, adjacent particles are often separated by residual ligands, adsorbed solvent, weakly bound organic species, or nanoscale voids.

The second potential benefit is crack suppression. During drying and organic decomposition, constrained shrinkage generates tensile stress in the film because the Mo-coated glass substrate restricts in-plane contraction [16,17,20].

The third possible benefit is partial compaction, but this term should be used only when supported by direct evidence such as thickness reduction, porosity decrease, mass-normalized density increase, profilometry, cross-sectional SEM/TEM, or image-based pore analysis. Without such evidence, the safer description is improved particle contact or improved film continuity rather than green-body compaction.

The fourth potential benefit is the reduction of organic residues. Pressure-assisted pre-sintering can be integrated with staged heating to remove weakly bound solvent and decompose or desorb surface ligands before the main chalcogenization step.

A key caution is that pressure-assisted pre-sintering should not be confused with pressure-assisted selenization or gas-pressure sintering in dense ceramics. Pre-sintering is a precursor-conditioning step before full chalcogenization; pressure-assisted selenization is a reactive chalcogenization step in which pressure may primarily control Se chemical potential, volatile Sn-containing species, vapor transport, transient liquid stability, and film integrity. The possible roles and cautions of pressure-assisted pre-sintering are summarized in Table 8.

Pressure-assisted pre-sintering can be considered a precursor-conditioning step that improves the quality of the nanoparticle-derived green film before full chalcogenization. Its possible benefits include improved particle contact, reduced cracking, partial consolidation of the precursor layer, and decreased organic residues. However, the role of pressure should be described carefully. Unless direct load transfer to the film is demonstrated by thickness reduction, porosity decrease, density increase, stress analysis, or cross-sectional compaction evidence, pressure-assisted pre-sintering should not be interpreted as conventional hot pressing.

The applied gas pressure may influence volatile release, organic-removal behavior, film integrity, and subsequent chalcogenization uniformity. Its role should be distinguished from direct mechanical pressure used in hot pressing or conventional gas-pressure sintering of bulk ceramics.

Critical summary: Pressure-assisted pre-sintering can improve particle contact and reduce pore volume before chalcogenization, but it is not automatically beneficial. Excessive compaction may trap organics, restrict chalcogen transport, or enhance nonuniform reactions; the method is useful only if densification is accompanied by clean interfaces, phase uniformity, and improved VOC/FF/JSC statistics.

## 4. Reactive Chalcogenization and Microstructural Evolution

After drying, burnout, and any precursor-conditioning step, the precursor compact must be converted into a crystalline chalcogenide absorber by sulfurization, selenization, or combined sulfo-selenization. This step is often described simply as “annealing,” but for nanoparticle-derived CIGS and CZTSSe films it is more accurately understood as reactive chalcogenization. The term “reactive chalcogenization” is preferred because it names the actual chemistry: chalcogen vapor transport, S/Se exchange, intermediate-phase conversion, cation redistribution, volatile-species control, grain coarsening, secondary-phase redistribution, and Mo back-contact reaction.

**Figure 12:**
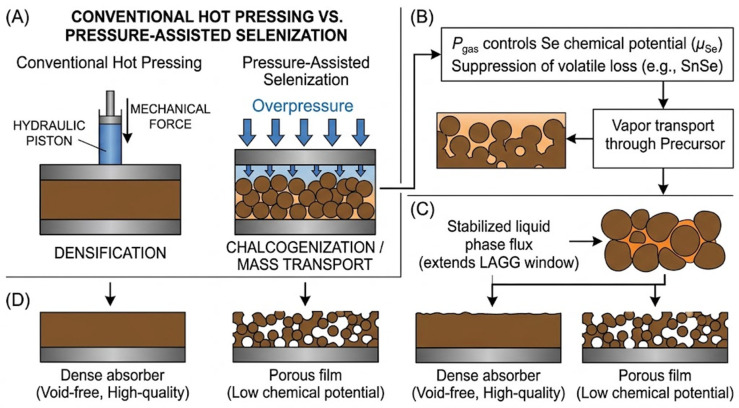
Schematic comparison between conventional hot pressing and pressure-assisted selenization. (**A**) Conventional hot pressing densifies a compact mainly through direct mechanical loading, whereas pressure-assisted selenization applies gas overpressure that primarily modifies the chalcogenization atmosphere and mass-transport conditions rather than directly compacting the film. (**B**) Elevated gas pressure increases the Se chemical potential, suppresses volatile-species loss such as Sn–Se evaporation, and promotes Se vapor transport through the porous precursor. (**C**) A sufficiently high Se chemical potential can stabilize transient liquid or liquid-like chalcogenide phases, extending the liquid-assisted grain-growth window and enhancing particle coalescence. (**D**) When vapor transport, volatile-species retention, and transient liquid formation are well balanced, a dense, high-quality absorber can be obtained; insufficient chemical potential or poorly controlled transport instead leaves a porous film with incomplete conversion and limited densification. AI-assisted schematic; scientific basis supported by Refs. [16,17,18,20,23,24,32,35].

As schematically shown in Figure 12, conventional hot pressing promotes densification mainly through direct mechanical force. In contrast, pressure-assisted selenization or related pressure-assisted chalcogenization primarily modifies the gas-phase environment. The applied gas pressure increases the Se chemical potential, suppresses volatile Sn–Se species loss, promotes vapor transport through the porous precursor, and may stabilize transient liquid or liquid-like chalcogenide phases. These effects can enhance mass transport, particle coalescence, and liquid-assisted grain growth, thereby improving absorber continuity. Therefore, the improved film density or morphology observed after pressure-assisted processing may arise from chemically enhanced chalcogenization and liquid-assisted coarsening rather than from direct mechanical compaction. This distinction should be clearly stated to avoid overinterpreting pressure-assisted processing as true hot pressing.

### 4.1. Chalcogenization as Reactive Sintering

**Figure 13:**
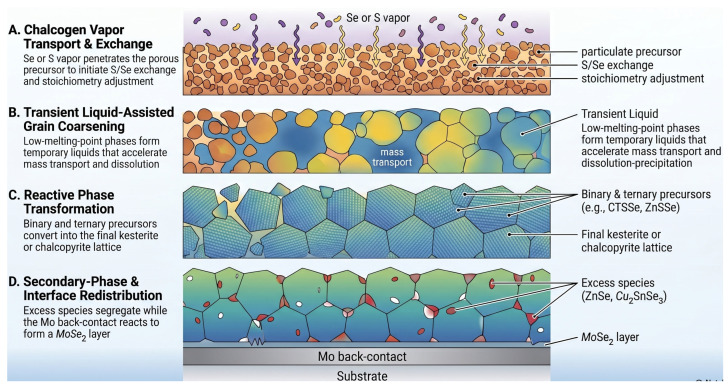
Reactive chalcogenization as a coupled conversion process. Selenization or sulfurization combines chalcogen vapor transport, S/Se exchange, phase transformation, transient liquid formation, grain coarsening, secondary-phase redistribution, and Mo back-contact reaction. AI-assisted schematic; scientific basis supported by Refs. [21,23,24,28,32,35].

Selenization or sulfurization of colloidal CIGS and CZTSSe precursor films can be viewed as analogous to reactive sintering only in the limited sense that chemical conversion and microstructural coarsening occur during the same thermal step. It is not directly equivalent to conventional oxide-ceramic sintering, where densification and grain growth are usually driven primarily by reduction of surface and interfacial energy in a comparatively stable solid framework. In chalcogenide absorbers, the dominant variables also include Se/S chemical potential, volatile Sn-containing species, liquid or vapor intermediates, narrow kesterite-phase stability, and reactive interfaces. Therefore, this review uses “reactive chalcogenization” for the actual absorber-forming process and reserves “reactive sintering” as a ceramic analogy that must be supported by mechanism-specific evidence. This coupled conversion process is summarized in Figure 13.

For CIGS absorbers, chalcogenization involves the conversion of Cu–In–Ga-containing precursors into the chalcopyrite Cu(In,Ga)Se_2_ phase. The reaction pathway may include Cu–Se, In–Se, Ga–Se, or mixed ternary intermediates, followed by interdiffusion and crystallization of the final chalcopyrite structure [10,14,22].

For CZTSSe absorbers, reactive chalcogenization is even more complex because the kesterite phase has a narrow thermodynamic stability window and contains four cations and two possible chalcogen species. When CZTS or mixed sulfide/selenide precursors are selenized, the film undergoes S/Se exchange, lattice expansion, cation redistribution, and transformation toward Cu_2_ZnSn(S,Se)_4_ [7,8,9,23,24].

A central feature of reactive chalcogenization is the role of vapor transport. The chalcogen source, such as elemental Se or S, is transported through the furnace atmosphere and reacts with the precursor film. This is a chalcogenide-specific chemical boundary that has no direct analog in ordinary oxide-ceramic sintering, because vapor-phase Se/S activity can determine phase stability, S/Se exchange, Sn retention, and MoSe_2_ formation.

Transient liquid or liquid-like phases can further accelerate grain growth and densification during chalcogenization, but this statement is mechanism-specific. In CZTSSe, liquid Se, Cu-Se, Sn-Se, or Cu-Sn-Se-related phases may promote liquid-assisted phase evolution, abnormal grain growth, and coarsening of the kesterite absorber [23,24]. However, the presence of large grains alone does not demonstrate liquid-assisted coarsening; the mechanism requires phase evidence from in situ or quenched experiments, Raman/XPS/TEM/EDS mapping, composition-depth profiles, or thermodynamic/kinetic analysis.

Reactive chalcogenization also includes important interface reactions. The Mo back contact is not inert under high-temperature Se- or S-containing atmospheres.

From this perspective, the success of reactive chalcogenization depends on balancing several coupled factors: sufficient chalcogen activity for complete conversion, controlled volatile-species loss, clean particle contacts after organic burnout, suitable pore structure for vapor transport, transient liquid formation for grain growth, and suppression of detrimental secondary phases and excessive back-contact reactions. For colloidal CIGS and CZTSSe absorbers, the objective is not simply to increase annealing temperature or time, but to design a reaction pathway that converts a constrained nanoparticle compact into a dense, phase-pure, compositionally uniform, and electronically benign absorber. The coupled processes involved in reactive chalcogenization are summarized in Table 9.

Critical summary: Describing chalcogenization as reactive sintering is helpful because it emphasizes simultaneous reaction, mass transport, coarsening, and densification. The weakness of the analogy is that chalcogenide absorbers are governed by volatile species, liquid-like intermediates, and narrow phase stability; therefore, the term should be used as an organizing framework, not as direct equivalence to oxide-ceramic sintering.

### 4.2. Pressure-Assisted Selenization

**Figure 14:**
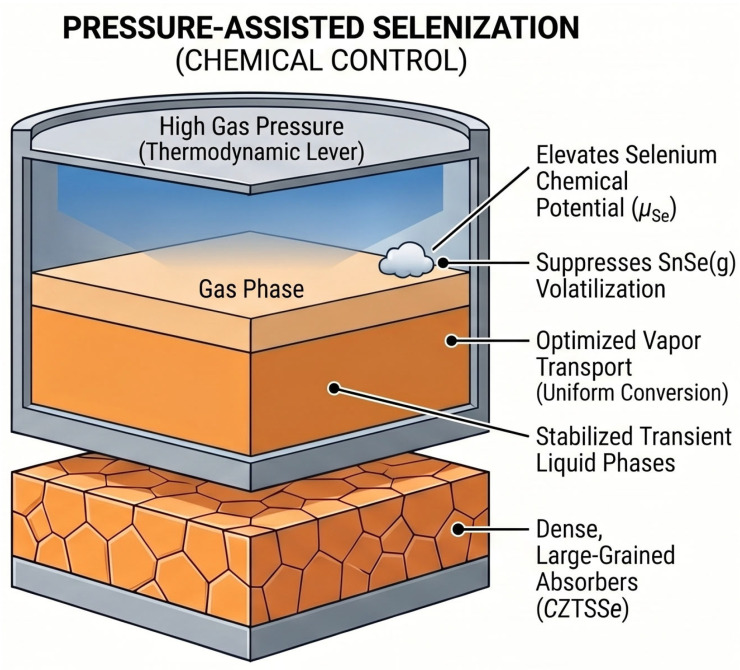
Distinct effects of pressure during pressure-assisted selenization. Gas pressure primarily modifies Se chemical potential, volatile-species loss, transport through a porous precursor, transient liquid stability, and film integrity rather than acting as conventional hot pressing. AI-assisted schematic; scientific basis supported by Refs. [23,24,32,35].

Pressure-assisted selenization has been proposed as a useful strategy for improving the conversion of nanoparticle-derived CIGS and CZTSSe precursor films into dense, crystalline absorber layers. However, the role of pressure should be described carefully and separated into chemical, transport, liquid-phase, volatile-species, mechanical, and interface contributions. These distinct pressure effects are separated schematically in Figure 14.

From a chemical perspective, the most important function of pressure-assisted selenization is to maintain a high Se chemical potential around the precursor film. During high-temperature selenization, Se vapor must continuously react with the precursor compact to complete S/Se exchange, stabilize the desired chalcopyrite or kesterite phase, and suppress chalcogen-deficient regions.

From a transport perspective, pressure-assisted selenization can influence how Se-containing species penetrate the porous precursor compact and how the reaction front moves through the film thickness. In nanoparticle-derived films, Se transport is governed by pore connectivity, film thickness, residual carbon, crack density, particle contacts, and the formation of transient liquid phases.

From a mechanical perspective, pressure may help reduce pore growth, crack opening, or volatile-induced film disruption during selenization. For example, a pressurized atmosphere may suppress rapid outgassing, limit the expansion of internal voids, and help maintain film continuity during phase transformation. The role of pressure during selenization is separated into chemical, transport, liquid-phase, volatile-species, mechanical, and interface effects in Table 10.

Pressure-assisted selenization is therefore best described as a process that modifies the chemical potential, vapor transport, volatile-species balance, transient liquid stability, and film integrity during chalcogenization. In CIGS systems, pressure may help maintain Se-rich conditions, promote chalcopyrite crystallization, and reduce porosity during nanoparticle-film conversion [22].

A rigorous description should therefore avoid statements such as “pressure directly forces Se into the film” or “pressure mechanically densifies the absorber” unless supported by direct evidence. A more accurate description is that pressure-assisted selenization can maintain Se activity, suppress volatile loss, modify reaction-front movement, stabilize or shift transient liquid-assisted coarsening, and reduce pore/crack evolution. Direct mechanical compaction should be claimed only when thickness, porosity, density, stress, or load-transfer measurements demonstrate it.

Pressure-assisted selenization can enhance absorber formation by maintaining Se activity, suppressing volatile loss, modifying Se transport, and stabilizing transient liquid-assisted grain growth. However, the Se chemical potential must be optimized rather than simply maximized. A high Se activity can promote S/Se exchange, kesterite crystallization, and transient-liquid-assisted grain growth, but it can also accelerate the reaction between Se and the Mo back contact to form MoSe_2_. Therefore, pressure-assisted selenization involves a kinetic competition between absorber coarsening and back-contact selenization. If MoSe_2_ grows too rapidly or becomes too thick, the associated interfacial volume expansion and stress development can disrupt bottom-layer coalescence, weaken adhesion, obstruct through-thickness mass transport, and contribute to the persistence of a fine-grained bottom layer. Its possible mechanical contribution to pore or crack suppression should be distinguished from direct hot pressing and should only be claimed when effective load transfer or measurable compaction is demonstrated.

Gas pressure may improve film continuity by reducing volatile-induced pore growth and by maintaining a more favorable chalcogenization environment, but it should not be treated as equivalent to mechanical compaction unless supported by thickness, density, porosity, or stress measurements.

Critical summary: Pressure-assisted selenization can stabilize volatile components and improve reaction uniformity, but it also changes vapor transport, liquid formation, and back-contact reactions. Its effectiveness should therefore be judged from Sn retention, S/Se gradients, secondary-phase mapping, MoSe_2_ thickness, and device metrics rather than from grain size alone.

### 4.3. Transient Liquid-Assisted Grain Growth

Transient liquid-assisted grain growth is one possible mechanism controlling the microstructural evolution of selenized CIGS and CZTSSe absorber films. In nanoparticle-derived precursor layers, the initial particles are usually much smaller than the final grains required for efficient carrier transport. However, this term should be used only when a liquid or liquid-like intermediate is identified or strongly supported; otherwise, the more general term “grain coarsening during reactive chalcogenization” is more appropriate.

**Figure 15:**
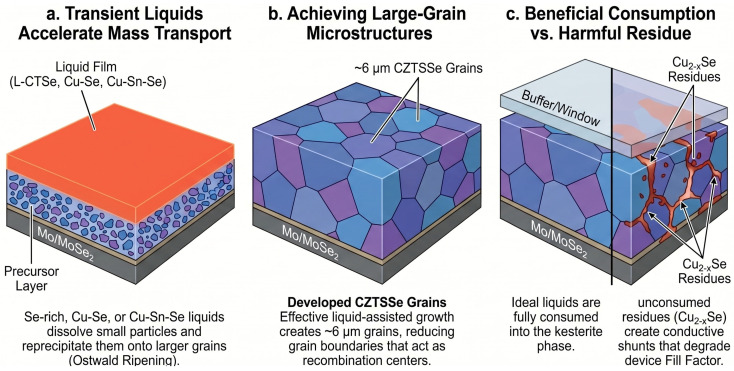
Transient liquid-assisted grain growth during CZTSSe selenization. Se-rich, Cu-Se-related, Cu-Sn-Se, Ag-Se-containing, or multicomponent chalcogenide liquids can accelerate dissolution-reprecipitation and grain coarsening, but residual conductive liquid-derived phases can degrade device performance. AI-assisted schematic; scientific basis supported by Refs. [23,24,32,35].

For CZTSSe, several possible liquid or liquid-like phases may participate during selenization. These include Se-rich liquids, Cu–Se phases, Sn–Se phases, Cu–Sn–Se liquids, Ag–Se-containing liquids in Ag-substituted systems, or more complex multicomponent chalcogenide liquids containing Cu, Zn, Sn, S, Se, and dopant elements [23,24,37]. These liquid or liquid-like species should not be treated as a single mechanism. At least five different pathways must be distinguished. First, a beneficial transient liquid can promote dissolution–reprecipitation, particle coalescence, pore elimination, and grain coarsening when it is short-lived, spatially well distributed, and consumed during kesterite formation. Second, a harmful residual liquid-derived phase can remain after selenization as Cu_2−x_Se, Cu–Sn–Se, or other conductive residues, producing shunting, nonselective recombination, and poor fill factor. Third, Sn–Se or Se-containing species may contribute through volatile-assisted transport rather than true liquid-phase sintering; in this case, the important issue is Sn retention and vapor-phase redistribution, not liquid-assisted densification. Fourth, surface-localized liquid formation may generate large grains near the top surface but fail to assist the bottom region, thereby producing a bilayer absorber with a large-grained upper layer and a fine-grained bottom layer. Fifth, back-interface liquid or highly reactive Se-rich chemistry near Mo can promote MoSe_2_ growth, interfacial voiding, or secondary-phase segregation instead of beneficial absorber coarsening. Therefore, the phrase “transient liquid-assisted grain growth” should be used only after specifying the composition, location, lifetime, and final consumption of the liquid-derived phase. The possible liquid-assisted growth pathways are summarized in Figure 15.

The fundamental role of a transient liquid phase is to increase the rate of mass transport. This description is valid only for a beneficial transient liquid. A liquid phase improves the absorber only when it enhances dissolution of small particles, reprecipitation on larger grains, interparticle neck growth, and pore elimination without leaving conductive or insulating residues. If the liquid is compositionally unbalanced, spatially localized, or incompletely consumed, it may increase grain size while simultaneously creating Cu_2−x_Se shunts, Cu–Sn–Se residues, ZnSe-rich blocking regions, Sn loss, or back-contact reaction products. Thus, grain coarsening alone should not be used as proof that liquid-assisted growth was beneficial. In a nanoparticle compact, small particles have high surface energy and therefore tend to dissolve more readily than larger particles.

In CZTSSe absorbers, Cu-containing liquid phases are often considered important because Cu–Se and Cu–Sn–Se compounds can form low-melting or liquid-like phases under selenization conditions [23]. These liquids can assist rapid grain growth and improve film continuity. However, Cu-containing liquid chemistry is a double-edged pathway. If the Cu–Se or Cu–Sn–Se liquid is transient and is fully incorporated into the growing kesterite phase, it can accelerate grain coarsening and improve film continuity. If it persists after chalcogenization, it may leave Cu_2−x_Se or Cu–Sn–Se residues at the surface, grain boundaries, or back contact. These residual liquid-derived phases are especially harmful because they can be conductive and may form shunting paths even when the absorber appears dense in cross-sectional SEM. Therefore, Cu-containing liquid formation should be evaluated by Raman spectroscopy, selective etching response where appropriate, cross-sectional TEM/EDS, XPS, and J–V shunt analysis, rather than by grain size alone.

Sn–Se-related species also require careful consideration. Sn-containing compounds can participate in transient liquid or vapor-mediated reactions, but Sn volatility is one of the major challenges in CZTSSe processing [7,8,9,24]. If the Se activity, temperature, or annealing time is not properly controlled, Sn loss can shift the absorber away from the desired kesterite stability window and promote formation of ZnSe, Cu_2_SnSe_3_, SnSe_2_, or other secondary phases. Therefore, any discussion of Sn–Se liquid-assisted growth should be accompanied by a discussion of Sn-retention control, Se chemical potential, and phase stability.

Sn–Se chemistry should also be separated into liquid-mediated and volatile-mediated pathways. Some Sn–Se-rich local compositions may participate in transient liquid or liquid-like reactions, but Sn-containing vapor species can also redistribute or remove Sn during selenization. In the latter case, the process is better described as volatile-assisted transport rather than liquid-assisted grain growth. This distinction matters because vapor-mediated Sn transport can help homogenize local composition under controlled conditions, but it can also drive Sn loss, shift the absorber out of the kesterite stability window, and promote ZnSe, SnSe_2_, or Cu_2_SnSe_3_ formation. Evidence for Sn–Se-mediated growth should therefore include Sn depth profiles, quenched intermediate phases, vapor/temperature dependence, and cross-sectional composition mapping.

Ag substitution can further modify liquid-assisted grain growth. Because Ag can interact with Se and may alter the melting behavior, liquidus temperature, and transport properties of the transient chalcogenide phase, moderate Ag incorporation has been reported to enhance grain growth and reduce fine-grained regions in some CZTSSe absorbers [23,24,37].

The dissolution–reprecipitation model also helps explain why precursor structure strongly affects the final microstructure. A well-packed green body with clean particle contacts can support continuous liquid redistribution and uniform grain coarsening.

Evidence for liquid-assisted growth should combine temperature-dependent or quenched-phase identification with cross-sectional microstructure, composition mapping, and device correlation rather than relying only on grain-size changes. The key question is not simply whether a liquid-like phase forms, but whether it acts as a beneficial transient transport medium, a harmful residual conductive phase, a volatile-assisted redistribution pathway, a surface-localized coarsening agent, or a back-interface reaction accelerator. These pathways are distinguished in Table 11, and mechanistic claims should be worded according to the level of evidence available.

More rigorously, the community should move beyond post-mortem identification of Cu-Se, Sn-Se, Cu-Sn-Se, or other liquid-derived residues and instead test the kinetics of phase dissolution during the actual annealing window. A transient liquid mechanism is convincingly demonstrated only when time- and temperature-resolved evidence shows when the liquid-like phase forms, which precursor or secondary phases dissolve into it, how rapidly the dissolved species are transported and reprecipitated into kesterite or chalcopyrite grains, and when the liquid is consumed. Therefore, the phase matrix in Table 11 should be interpreted as a mechanistic checklist rather than a final proof: thermodynamic or chemical-potential modeling must be coupled with time-resolved structural data from in situ or operando XRD, Raman spectroscopy, synchrotron scattering, or interrupted-quench experiments before liquid-assisted grain growth is claimed as the dominant mechanism.

Transient chalcogenide liquids can enhance mass transport and promote dissolution–reprecipitation during selenization, but this statement applies only to liquid phases that are transient, spatially connected, compositionally balanced, and consumed during absorber formation. A liquid-derived residue is not equivalent to a beneficial transient liquid. Cu_2−x_Se, Cu–Sn–Se, or Cu_2_SnSe_3_ residues may remain after selenization and degrade the device by shunting, nonselective recombination, or local band inhomogeneity. Similarly, Sn–Se species may contribute through volatile-assisted transport rather than true liquid-phase sintering; therefore, Sn retention, vapor-phase redistribution, and Sn depth profiles must be considered. Surface-localized liquid formation can explain why large grains develop near the top surface while a fine-grained bottom layer persists near Mo. Back-interface Se-rich or Cu–Sn–Se reaction zones can accelerate MoSe_2_ formation, interfacial voiding, and secondary-phase segregation. Therefore, any claim of beneficial transient-liquid-assisted grain growth should be supported by quenched or in situ phase analysis, cross-sectional Raman/TEM/EDS, depth-resolved composition, and device-level correlation.

Ag substitution may modify transient liquid chemistry and enhance grain coarsening, but the actual liquid phase in CZTSSe is likely multicomponent. Therefore, Ag-assisted grain growth should be discussed as a coupled effect of altered liquid or liquid-like reaction pathways, cation redistribution, defect chemistry, and microstructural evolution unless specific Ag–Se liquid formation is experimentally confirmed.

Critical summary: Transient liquid-assisted coarsening is a plausible route to larger grains and improved mass transport, but it is also one of the easiest mechanisms to overstate. A beneficial liquid must be transient, spatially connected, compositionally controlled, and consumed; more importantly, it must be demonstrated kinetically by tracking phase formation, dissolution, transport, reprecipitation, and disappearance rather than inferred only from post-mortem grain size or residual phases. Thermodynamic modeling coupled with time-resolved structural data is therefore required to distinguish a true transient liquid pathway from volatile-assisted transport, secondary-phase segregation, or surface-localized coarsening.

### 4.4. Bilayer Microstructure

**Figure 16:**
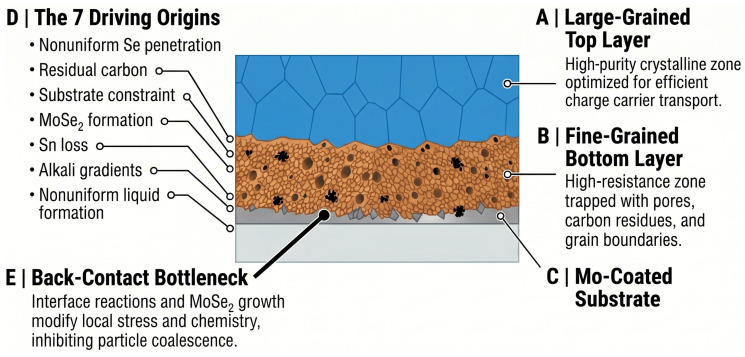
Origin of bilayer microstructure in nanoparticle-derived CZTSSe absorbers. A large-grained top layer and fine-grained bottom layer may result from nonuniform Se penetration, residual carbon, substrate constraint, MoSe_2_ formation, Sn loss, alkali gradients, and spatially nonuniform transient liquid formation. AI-assisted schematic; scientific basis supported by Refs. [21,29,30,31,32,34,35,48].

A characteristic microstructural feature frequently observed in nanoparticle-derived CZTSSe absorbers is the formation of a bilayer structure, consisting of a large-grained upper layer and a fine-grained bottom layer near the Mo back contact. The large-grained top layer is generally favorable for carrier transport because it contains fewer grain boundaries and provides more continuous pathways for photogenerated carriers. In contrast, the fine-grained bottom layer is usually detrimental because it contains a high density of grain boundaries, residual pores, secondary phases, carbonaceous residues, and back-contact reaction products. The coupled origins of the bilayer microstructure are summarized in Figure 16. In particular, surface-localized transient liquid formation can accelerate top-layer coarsening while failing to supply the bottom region with sufficient liquid-mediated mass transport, thereby producing the common large-grained-top/fine-grained-bottom bilayer morphology.

One major cause of bilayer formation is nonuniform Se penetration. During selenization, Se vapor reaches the film from the external atmosphere and reacts first with the surface region of the precursor compact.

A second important factor is residual carbon. Long-chain ligands such as oleylamine, if not completely removed before chalcogenization, can leave carbonaceous residues at particle contacts and grain boundaries [34].

Substrate constraint also contributes to bilayer formation. The precursor film is attached to a rigid Mo-coated soda-lime glass substrate; therefore, shrinkage, particle rearrangement, and grain growth near the back contact are mechanically constrained [16,17].

Another important factor is MoSe_2_ formation at the back contact. During selenization, Se reacts with Mo to form MoSe_2_, and this reaction proceeds simultaneously with kesterite phase formation and grain coarsening in the adjacent absorber layer [29,30,31,32,35]. The growth of MoSe_2_ should therefore be viewed as a competing ceramic interface reaction. A thin and continuous MoSe_2_ layer may assist back-contact formation, but excessive MoSe_2_ growth consumes Se near the interface, introduces interfacial volume expansion, generates local stress, and can mechanically disrupt the bottom region of the absorber [29,30,31,35]. This disruption suppresses bottom-up grain coarsening, promotes interfacial voiding or delamination, and contributes to the persistence of a fine-grained bottom layer near the Mo contact [21,32,35]. Therefore, MoSe_2_ control is not only an electrical back-contact issue; it is also a kinetic and mechanical constraint on microstructural evolution during reactive chalcogenization. This reaction should be treated as a kinetic competitor to kesterite grain coarsening rather than only as a passive back-contact reaction [29,30,31,32,35]. During reactive chalcogenization, Se supplied from the vapor phase or transient liquid phase must simultaneously participate in S/Se exchange, CZTSSe phase formation, liquid-assisted grain growth, and Mo selenization. Near the back contact, the Mo + Se → MoSe_2_ reaction can consume Se locally and generate a growing interfacial MoSe_2_ layer while the bottom CZTSSe grains are still attempting to coarsen [29,30,31,32,35]. If MoSe_2_ growth is rapid or excessive, the associated volume expansion and interfacial stress can physically disturb the bottom absorber region, interrupt particle coalescence, weaken adhesion, open interfacial voids, and hinder bottom-up grain growth. Thus, the fine-grained bottom layer is not only a mass-transport or residual-carbon problem; it is also a ceramic interface-reaction problem governed by competition between absorber crystallization and back-contact selenization [21,29,30,31,32,35].

Sn loss can further promote through-thickness microstructural inhomogeneity. CZTSSe has a narrow phase-stability window, and Sn-containing species can volatilize during high-temperature selenization [7,8,9,24].

Finally, alkali diffusion gradients may influence bilayer evolution. In many chalcogenide solar cells, Na and other alkali elements diffuse from soda-lime glass through the Mo back contact into the absorber during annealing.

The bilayer structure should therefore be treated as a multifactorial processing–microstructure–interface problem. Nonuniform Se penetration, residual carbon, substrate constraint, MoSe_2_ formation, Sn loss, and alkali diffusion gradients may act simultaneously and may reinforce one another.

A rigorous discussion of bilayer microstructure should therefore combine cross-sectional microstructure, depth-resolved composition, phase mapping, and device analysis. Cross-sectional SEM can reveal the thickness and morphology of the fine-grained bottom layer. The likely causes of bilayer formation and their device consequences are summarized in Table 12.

The bilayer structure is likely produced by several coupled factors, including nonuniform Se penetration, residual carbon, substrate constraint, MoSe_2_ formation, Sn loss, alkali diffusion gradients, and spatially nonuniform transient liquid formation.

Pressure-assisted selenization may reduce the fine-grained bottom layer by improving Se activity and reaction-front movement, but complete suppression also requires control of residual carbon, precursor packing, Sn retention, MoSe_2_ growth, and back-interface chemistry [21,32,35]. In particular, MoSe_2_ thickness should be interpreted together with the kinetics of bottom-layer grain growth. A thin MoSe_2_ layer may provide a useful transition between Mo and the absorber, but a thick or nonuniform MoSe_2_ layer indicates that Se consumption and volume expansion at the back contact have become competitive with absorber coarsening [29,30,31,35]. In this case, the bottom-layer grains may remain small not because the system lacks a grain-growth driving force, but because the growing MoSe_2_ interface mechanically and chemically interrupts the coarsening pathway. Therefore, bilayer suppression requires simultaneous control of Se chemical potential, MoSe_2_ reaction rate, precursor packing, residual carbon, Sn retention, and transient liquid distribution [21,29,30,31,32,35].

Critical summary: Bilayer microstructure is a particularly important weakness of nanoparticle-derived absorbers because a dense top layer can mask a fine-grained, recombination-active bottom layer. The critical point is that cross-sectional morphology must be interpreted together with back-contact chemistry, long-wavelength EQE, TRPL/PL, SIMS, and TEM/EELS evidence [21,29,30,31,32,35].

## 5. Phase Evolution, Secondary Phases, and Defect Chemistry

Phase evolution, secondary-phase formation, and defect chemistry are tightly coupled during the conversion of colloidal CIGS and CZTSSe precursor films into functional photovoltaic absorbers. Although both systems belong to the family of multinary chalcogenide semiconductors, their reaction pathways and defect sensitivities are very different.

### 5.1. Phase Evolution

#### 5.1.1. Phase Evolution in CIGS Absorbers

**Figure 17:**
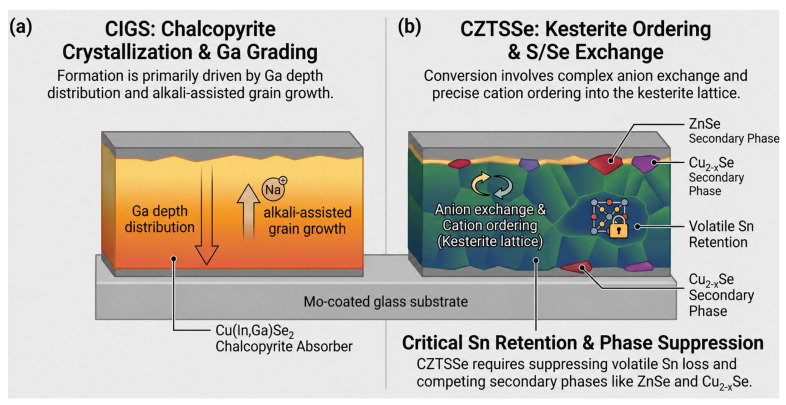
Comparison of phase evolution in CIGS and CZTSSe absorbers. CIGS formation is governed by chalcopyrite crystallization, Ga distribution, and alkali-assisted grain growth, whereas CZTSSe formation additionally involves S/Se exchange, Sn retention, cation ordering, and suppression of competing selenides. AI-assisted schematic; scientific basis supported by Refs. [5,10,11,12,13,14,23,24,25,28,32,35].

The formation of chalcopyrite Cu(In,Ga)Se_2_ from colloidal or nanoparticle-derived precursors usually proceeds through a sequence of binary and ternary intermediate phases before the final quaternary chalcopyrite phase is established. Depending on the precursor chemistry, heating profile, Se activity, and particle-size distribution, Cu–Se, In–Se, Ga–Se, Cu–In–Se, and Cu–Ga–Se intermediates may form transiently during selenization [10,14,22]. The phase-evolution pathways of the two absorber families are compared in Figure 17.

The formation of single-phase CIGS requires sufficient interdiffusion among Cu, In, Ga, and Se. In colloidal routes, the precursor particles may already contain some degree of chalcopyrite or related crystallinity, but additional high-temperature selenization is usually necessary to improve grain size, remove residual porosity, and complete compositional homogenization [10,14,22].

In high-performance CIGS absorbers, controlled Ga grading is often beneficial. A higher Ga concentration near the back region can increase the local band gap and help reduce back-surface recombination, whereas excessive Ga near the front interface may create a barrier to carrier collection if not properly controlled [5,9].

Alkali elements, especially Na and K, also play a central role in CIGS phase evolution and device performance. Na can diffuse from soda-lime glass through the Mo back contact during high-temperature processing, while K may be introduced through post-deposition treatment or controlled alkali incorporation [9].

Critical summary: CIGS phase evolution is comparatively forgiving because the chalcopyrite system tolerates some compositional variation and benefits from established Ga and alkali engineering. Its remaining weakness in colloidal routes is maintaining controlled gradients, low impurity levels, and interface quality during non-vacuum conversion.

#### 5.1.2. Phase Evolution in CZTSSe Absorbers

The phase evolution of CZTSSe is more complex than that of CIGS because the kesterite structure contains four cations and two exchangeable chalcogen species. In many colloidal or nanoparticle-derived routes, the precursor may initially be CZTS, CZTSe, or a mixed sulfide/selenide compound.

Unlike CIGS, CZTSSe has a narrow thermodynamic stability window. Local deviations from the desired Cu-poor/Zn-rich composition can generate secondary phases such as conductive Cu_2−x_Se, insulating ZnSe, SnSe_2_-related phases, SnSe, or Cu_2_SnSe_3_ [7,8,9,23,24,38].

Sn volatility is another major challenge in CZTSSe phase evolution. At high selenization temperatures, Sn-containing species may evaporate or redistribute, especially under conditions of low Se activity or prolonged annealing [7,8,9,24].

Kesterite ordering further distinguishes CZTSSe from CIGS. The similar ionic sizes and charges of Cu^+^ and Zn^2+^ promote Cu/Zn antisite disorder, especially between the 2c and 2d cation sites in the kesterite lattice [7,8].

Another complication is that X-ray diffraction alone cannot reliably distinguish kesterite CZTSSe from several secondary phases because of peak overlap. This limitation should be stated more strongly: a claim of “phase-pure” CZTSSe based only on standard laboratory XRD is not reliable and should be regarded as a fundamental characterization weakness. In particular, ZnSe and Cu_2_SnSe_3_ can produce diffraction features that overlap with or closely resemble kesterite CZTSSe reflections, while minor Cu_2−x_Se, Sn–Se, or interface-localized phases may remain below the detection limit of XRD. Therefore, XRD should be used only as an initial phase-screening method, not as definitive proof of phase purity. ZnSe, Cu_2_SnSe_3_, and related phases may show diffraction peaks close to those of CZTSSe [7,8,9,38]. The phase-evolution issues in CIGS and CZTSSe are compared in Table 13.

Critical summary: CZTSSe phase evolution is less forgiving because the stability window is narrow and Sn volatility, Cu/Zn disorder, and secondary phases are strongly coupled. The main strength of processing control is the ability to suppress extrinsic phase errors, but the weakness is that intrinsic disorder and deep defects can remain even in apparently single-phase films.

### 5.2. Secondary Phases

Secondary-phase control is one of the most critical issues in CIGS and especially CZTSSe absorber processing. In multinary chalcogenide systems, the desired absorber phase forms within a limited compositional and thermodynamic window.

**Figure 18:**
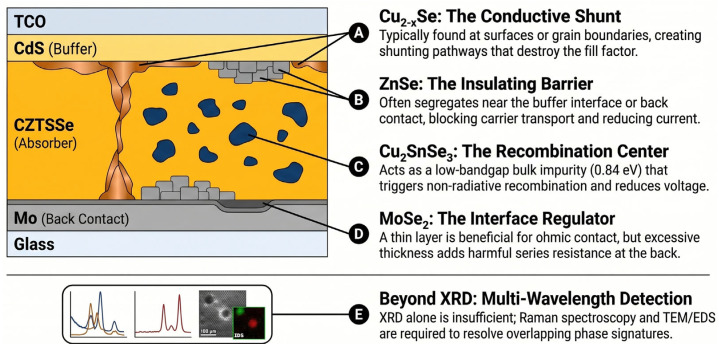
Secondary phases in CZTSSe absorbers and their detection. Cu_2−x_Se, ZnSe, Sn-Se phases, Cu_2_SnSe_3_, and excessive MoSe_2_ differ in location, electrical effect, and detectability. AI-assisted schematic; scientific basis supported by Refs. [25,26,27,28,30,31,32,38].

For CZTSSe absorbers, secondary-phase identification is particularly challenging because several phases have diffraction peaks that overlap with those of the kesterite structure. ZnSe, Cu_2_SnSe_3_, and other related selenides may be difficult to distinguish from CZTSSe by X-ray diffraction alone [7,8,9,38]. The secondary-phase detection problem is summarized in Figure 18. The origin, electrical effect, and detection limits of representative secondary phases are summarized in Table 14.

Cu_2−x_Se is among the most detrimental secondary phases in CZTSSe because it is highly conductive and can create shunting pathways through the absorber or along grain boundaries. It is often associated with Cu-rich local regions or incomplete consumption of Cu–Se-related transient liquids during selenization [7,8,9,23].

ZnSe is another important secondary phase, but its effect is different from that of Cu_2−x_Se. ZnSe is generally more insulating and may act as a carrier-blocking barrier when located at grain boundaries, the absorber surface, or the absorber/buffer interface [38].

SnSe_2_ and related Sn–Se phases may form when local Sn and Se activities are not properly controlled. These phases can appear near the surface, within the bulk, or as intermediate reaction products during selenization.

Cu_2_SnSe_3_ is commonly discussed as an intermediate or competing ternary phase during CZTSSe formation. It can form when Zn incorporation is incomplete or when local Cu–Sn–Se chemistry dominates the reaction pathway [7,8,9,23].

MoSe_2_ formation at the back contact must also be treated carefully. During selenization, Se reacts with the Mo back electrode to form MoSe_2_. The major secondary phases, typical locations, and device effects are summarized in Table 15.

The location of a secondary phase is often more important than its total amount. A small quantity of Cu_2−x_Se at the surface or along connected grain boundaries can be much more harmful than isolated inclusions buried within the absorber.

For CIGS, secondary-phase issues are generally less severe than in CZTSSe but remain important. Cu–Se phases may appear under Cu-rich conditions, and Ga-related compositional gradients can influence phase evolution and band-gap distribution. MoSe_2_ formation also occurs at the CIGS/Mo interface and must be controlled. However, because CIGS is more defect-tolerant and has a broader processing window, secondary phases in CIGS are often more manageable than in CZTSSe. In contrast, CZTSSe requires simultaneous control of Cu-poor/Zn-rich composition, Sn retention, S/Se exchange, kesterite ordering, and secondary-phase suppression.

Because the diffraction peaks of kesterite CZTSSe overlap with those of several secondary phases, standard XRD alone cannot confirm phase purity. This point should be emphasized explicitly because an XRD-only claim of “single-phase” or “phase-pure” CZTSSe is a fundamental flaw in many reports on kesterite absorbers. ZnSe and Cu_2_SnSe_3_ are particularly problematic because their diffraction peaks can overlap with the major kesterite reflections, while Cu_2−x_Se, SnSe_2_/Sn–Se phases, and MoSe_2_ may be present as minor, surface-segregated, grain-boundary, or interface-localized phases that are not detected reliably by conventional θ–2θ XRD. Therefore, XRD should be regarded as a preliminary structural screening tool rather than a definitive phase-purity test. Reliable verification of CZTSSe phase purity requires Raman spectroscopy using multiple excitation wavelengths, preferably combined with surface and cross-sectional Raman mapping, together with cross-sectional TEM/EDS to locate secondary phases at the absorber surface, grain boundaries, absorber/buffer interface, and Mo back contact. XPS, SIMS, GDOES, or other depth-resolved methods should be used when surface segregation, Sn imbalance, alkali gradients, or buried interface phases are suspected.

Cu-poor/Zn-rich composition can suppress conductive Cu-rich phases, but excessive Zn may promote ZnSe segregation, which can act as an insulating barrier at grain boundaries or interfaces.

Critical summary: Secondary-phase control is essential because minor Cu-, Zn-, Sn-, or Mo-containing phases can dominate shunting, blocking, or recombination despite being difficult to detect by XRD. The strength of Raman/XPS/SIMS/TEM approaches is local sensitivity, but each method has sampling or surface-depth limitations; robust conclusions require correlated, spatially resolved characterization and device response.

### 5.3. Defect Chemistry

Defect chemistry is the central reason CZTSSe remains less efficient than CIGS despite its attractive earth-abundant composition and similar thin-film device architecture. In CIGS, many intrinsic defects are relatively benign, and Cu-poor growth can produce electronically favorable defect complexes.

The most important acceptor defect in CZTSSe is the copper vacancy, V_Cu_. Under Cu-poor growth conditions, V_Cu_ is readily formed and acts as a shallow acceptor, contributing to p-type conductivity [7,8].

**Figure 19:**
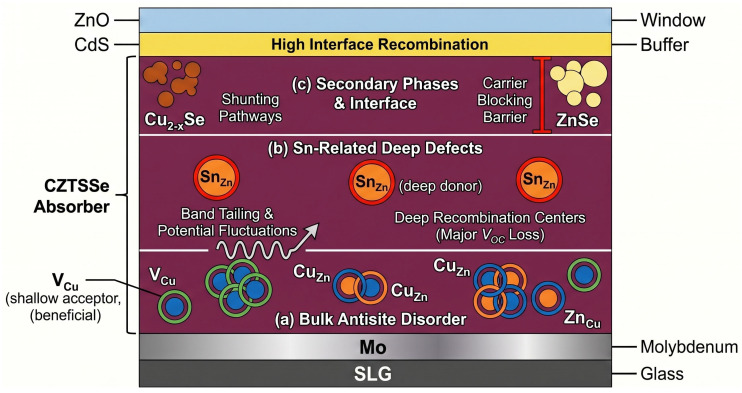
Defect chemistry and loss pathways in CZTSSe. Beneficial shallow acceptors such as V_Cu_ coexist with Cu/Zn antisite disorder, Zn_Cu_ compensation, Sn-related deep defects, band tailing, and interface recombination, producing a large open-circuit-voltage deficit. AI-assisted schematic; scientific basis supported by Refs. [7,8,9,36,37,38,39].

The dominant intrinsic disorder problem in CZTSSe is associated with Cu_Zn_ and Zn_Cu_ antisite defects. Because Cu^+^ and Zn^2+^ have similar ionic sizes and can occupy related crystallographic sites in the kesterite lattice, Cu/Zn antisite disorder has a low formation energy [7,8]. The main defect-related loss pathways are summarized in Figure 19.

Sn-related defects are also highly detrimental. Among them, Sn_Zn_ is commonly regarded as a deep donor-type defect that can introduce recombination-active states within the band gap [7,8].

Cu/Zn disorder is particularly harmful because it affects the electronic structure even when no obvious secondary phases are detected. Disorder broadens the band edges, produces spatial potential fluctuations, and increases the Urbach tail energy.

The V_OC_ deficit in CZTSSe is therefore a combined consequence of bulk defect chemistry, band tailing, secondary phases, and interface recombination. V_Cu_ acceptors are useful for p-type conductivity, but excessive compensation by donor-like defects can reduce carrier quality.

A common oversimplification is to state that a Cu-poor/Zn-rich composition suppresses defects. This statement should be revised because Cu-poor/Zn-rich processing solves only part of the defect problem. Cu-poor conditions are useful for suppressing conductive ${Cu}_{2-x}$ Se secondary phases and for promoting $V_{Cu}$-related p-type conductivity, while Zn-rich conditions can help avoid Cu-rich compositions. However, these compositional choices do not thermodynamically eliminate Cu/Zn antisite disorder. The formation energy of $Cu_{Zn}$, $Zn_{Cu}$, and associated antisite pairs remains low because the kesterite lattice permits facile Cu–Zn site exchange. Therefore, the absorber may still exhibit band-edge fluctuations, increased Urbach tail energy, electrostatic potential disorder, and a large V_OC_ deficit even when the microstructure is dense and the average composition is Cu-poor/Zn-rich. Excessive Zn-rich processing may also promote ZnSe segregation, creating carrier-blocking barriers at grain boundaries or interfaces. Thus, Cu-poor/Zn-rich composition should be described as a necessary phase-control strategy, not as a sufficient defect-control strategy. The key point defects and defect-related loss mechanisms are summarized in Table 16.

Defect chemistry should therefore be analyzed using methods beyond conventional structural characterization. XRD can provide information about lattice parameters and phase formation, but it cannot reliably quantify Cu/Zn disorder or identify all electronically active defects.

In addition to these optical and electrical diagnostics, element-specific X-ray absorption spectroscopy should be included in the defect-chemistry toolkit. X-ray Absorption Near Edge Structure (XANES) and Extended X-ray Absorption Fine Structure (EXAFS) probe the local electronic and coordination environment of a selected absorbing element rather than the long-range average crystal structure measured by XRD [49,50]. For CZTSSe, Cu-, Zn-, and Sn-edge XANES/EXAFS can therefore test whether apparently phase-pure absorbers contain local Cu/Zn site disorder, coordination distortion, or Sn-valence changes that are not resolved by laboratory XRD. In particular, Sn-edge XANES compared with Sn(II) and Sn(IV) standards can help identify changes in Sn oxidation state associated with Sn volatility, Sn off-stoichiometry, and Sn-related deep-defect formation, whereas EXAFS can provide nearest-neighbor distances, coordination numbers, and disorder parameters around Cu, Zn, and Sn sites. These measurements should not replace Raman, PL/TRPL, admittance, DLTS/TAS, EQE, or temperature-dependent J-V analysis; rather, they provide the missing local structural and valence evidence needed to connect cation disorder and Sn chemistry with the electronic loss mechanisms.

Cu-poor/Zn-rich composition suppresses conductive Cu-rich secondary phases and favors $V_{Cu}$-related p-type conductivity, but it does not automatically eliminate Cu/Zn antisite disorder. This distinction is critical because the dominant $V_{OC}$ deficit in CZTSSe is not simply a consequence of poor morphology. Even after effective ligand burnout, improved particle coalescence, reduced porosity, and controlled selenization, the kesterite lattice can retain low-energy $Cu_{Zn}$, $Zn_{Cu}$, and $\left[Cu_{Zn}+Zn_{Cu}\right]$ antisite disorder. These defects generate band tailing and potential fluctuations that reduce the effective absorber band gap and increase nonradiative recombination. Sn-related deep defects, especially under nonideal Sn and Se chemical potentials, further aggravate voltage loss. Therefore, a dense large-grained microstructure can reduce extrinsic recombination and transport loss, but it does not by itself confirm reduced antisite disorder, lower Sn-related deep-defect density, weaker band tailing, or a smaller *V*_OC_ deficit. Defect-sensitive measurements such as temperature-dependent PL, Urbach-energy analysis, Raman order–disorder assessment, admittance spectroscopy, SIMS, TRPL, and temperature-dependent J–V analysis are required to distinguish morphological improvement from true defect-chemical improvement.

Critical summary: Defect chemistry is the core limitation that separates CZTSSe from more defect-tolerant CIGS. Processing can reduce extrinsic defects and secondary phases, but it cannot be considered successful unless it also reduces local cation disorder, Sn-valence instability, band tailing, deep recombination, compensation, and VOC deficit as measured by correlated XANES/EXAFS, PL, TRPL, temperature-dependent J-V, admittance, DLTS/TAS, EQE, and Raman disorder analysis.

## 6. Alloying, Surface Cleaning, and Interface Engineering

Alloying, surface cleaning, and interface engineering are essential strategies for improving colloidal CZTSSe solar cells because device performance is not determined by microstructure alone. This point is particularly important for CZTSSe because the *V*_OC_ deficit is not removed simply by producing a dense absorber. Alloying, cation-ordering control, surface cleaning, and interface passivation are required to address the intrinsic defect-chemical and band-tail limitations that remain after ceramic-processing defects have been reduced. Even when a dense and large-grained absorber is obtained, the efficiency can still be limited by Cu/Zn disorder, Sn-related deep defects, secondary phases, band tailing, unfavorable band alignment, and recombination at the absorber/buffer or absorber/back-contact interfaces [7,8,9,24,38].

### 6.1. Alloying Strategies

**Figure 20:**
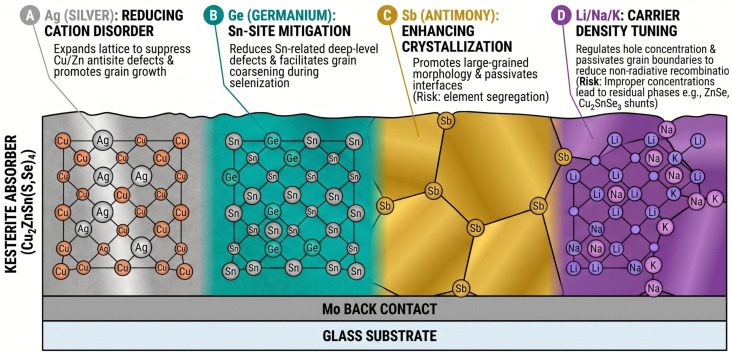
Alloying strategies for kesterite absorbers. Ag, Ge, Sb, Li/Na/K, and related additives can modify cation disorder, defect levels, grain growth, liquid-phase behavior, and interface recombination, but their benefits depend strongly on concentration, location, and phase stability. AI-assisted schematic; scientific basis supported by Refs. [37,47,51,52,53,54,55].

Cation alloying is widely used to modify the defect landscape and microstructural evolution of kesterite absorbers. The main objective is to reduce the electronic penalty associated with Cu/Zn disorder, suppress deep recombination centers, enhance grain growth, and improve carrier transport. However, alloying should not be treated as universally beneficial. Each dopant or alloying element introduces both advantages and risks, and the final effect depends strongly on concentration, spatial distribution, incorporation site, processing atmosphere, and interaction with secondary phases. The major alloying strategies and their trade-offs are summarized in Figure 20.

Critical summary: Alloying is attractive because small compositional changes can modify disorder, band alignment, grain growth, and defect formation. Its weakness is the risk of trading one loss mechanism for another; therefore, alloying should be evaluated through both microstructural/chemical analysis and device diagnostics rather than by efficiency improvement alone.

#### 6.1.1. Ag Substitution

Ag substitution is one of the most studied cation-alloying strategies for CZTSSe. Because Ag^+^ is larger than Cu^+^, partial substitution of Ag for Cu can expand the kesterite lattice and may reduce the tendency for Cu/Zn antisite disorder [51].

However, Ag substitution also introduces important risks. Excessive Ag incorporation can widen the absorber band gap, which may improve V_OC_ but reduce short-circuit current density, J_SC_, if absorption or carrier collection is compromised. Ag may also suppress hole concentration, depending on how it modifies intrinsic defects and compensation. If Ag is not uniformly incorporated, Ag-rich regions or Ag-containing secondary phases may form, causing local compositional inhomogeneity and possible recombination. Therefore, Ag substitution should be described as a concentration-sensitive strategy rather than a simple defect-removal solution.

A balanced statement is therefore necessary: Moderate Ag substitution may reduce Cu/Zn disorder, expand the lattice, promote grain growth, and improve carrier transport; however, excessive or nonuniform Ag incorporation may widen the band gap, reduce J_SC_, alter carrier concentration, or generate Ag-containing secondary phases.

Critical summary: Ag substitution can reduce Cu/Zn disorder and modify liquid-assisted growth, but excessive Ag may introduce phase segregation or band-gap/current trade-offs. The strategy is strongest when Ag distribution is homogeneous and the resulting improvement is confirmed by lower tailing, better PL yield, and improved VOC without sacrificing JSC.

#### 6.1.2. Ge Alloying

Ge alloying is another promising strategy because Ge can partially substitute for Sn and may reduce Sn-related defect problems. Since Sn-related defects such as Sn_Zn_ are considered deep recombination centers, Ge incorporation has been explored to improve defect chemistry, promote crystallization, and reduce nonradiative recombination [37,52]. Surface Ge treatments and Ge-containing precursor designs may also influence grain growth and the back-contact region. In some reports, Ge incorporation promotes larger grains and improves device parameters, indicating that Ge may modify both microstructure and electronic quality [37,52].

Nevertheless, Ge alloying must be controlled carefully. Excessive Ge can change the band gap, alter band alignment, or introduce Ge-rich secondary phases. Because Ge and Sn have different chemical behavior and volatility, the Ge incorporation pathway may also affect local phase evolution during selenization. Therefore, Ge should be discussed as a defect- and crystallization-modifying additive, not merely as a grain-growth promoter.

Critical summary: Ge can assist crystallization, defect control, and Sn-related chemistry, but its beneficial role is highly process-dependent. The weakness is that Ge redistribution or excessive incorporation can complicate phase stability; atom-scale composition mapping and recombination analysis are needed before assigning a universal mechanism.

#### 6.1.3. Sb Treatment

Sb has been investigated as a dopant or surface-treatment element for promoting grain growth and suppressing defects in CZTSSe. Sb-containing species may influence crystallization, reduce defect density, and passivate bulk or interfacial recombination centers [53]. Some studies suggest that Sb incorporation can promote bottom grain growth, reduce surface roughness, and improve heterojunction transport properties. This is particularly relevant to nanoparticle-derived films, where bottom fine grains and interface recombination are major limitations.

However, Sb addition also requires caution. Sb may not be uniformly incorporated into the kesterite lattice and may instead segregate at surfaces, grain boundaries, or interfaces. Excessive Sb can form secondary phases or modify local band alignment unfavorably. Therefore, Sb treatment should be evaluated by combining Raman, XPS, SIMS, TEM/EDS, PL, and device analysis to determine whether it truly passivates defects or simply changes morphology.

Critical summary: Sb treatment is promising because it can passivate traps and influence grain growth, but it must be controlled carefully to avoid new secondary phases or surface-segregated residues. Its success should be judged from defect-level spectroscopy, PL/TRPL, chemical mapping, and reproducible device gains.

#### 6.1.4. Li Doping and Alkali Treatments

Li doping and broader alkali treatments are also important for CZTSSe because alkali elements can influence carrier concentration, defect passivation, grain growth, and interface chemistry. Li may occupy specific lattice or interstitial sites and influence acceptor formation, carrier density, and compensation.

The risks of alkali treatments should also be stated. Excessive alkali concentration can cause secondary phases, nonuniform depth distribution, interface instability, or overcompensation. In nanoparticle-derived films, alkali diffusion may be affected by residual carbon, porosity, MoSe_2_ thickness, and precursor packing. Therefore, alkali treatments must be optimized with respect to depth profile, grain-boundary distribution, and absorber/buffer interface chemistry. The benefits and risks of common alloying strategies are summarized in Table 17.

Moderate Ag substitution may reduce Cu/Zn disorder, expand the lattice, promote grain growth, and improve carrier transport, but excessive or nonuniform Ag incorporation can widen the band gap, reduce J_SC_, alter carrier concentration, or generate Ag-containing secondary phases.

Ge, Sb, and Li modify CZTSSe through different mechanisms. Ge mainly affects Sn-related defect chemistry and crystallization, Sb can promote grain growth and passivate bulk/interface defects, while Li and other alkali elements can regulate carrier concentration and grain-boundary chemistry. Their effects depend strongly on concentration, incorporation site, and depth distribution.

Representative recent reports support that Ag substitution is used to suppress Cu/Zn disorder and regulate defects, Ge incorporation can manipulate defects and morphology, Sb doping can promote grain growth and passivate defects, and Li/alkali treatments can tune carrier concentration and passivation behavior.

Critical summary: Alkali treatments are among the most powerful empirical routes for improving chalcogenide solar cells, but they are also difficult to interpret because they affect grain growth, interfaces, carrier density, and defect passivation simultaneously. The critical requirement is to separate beneficial passivation from changes in morphology, compensation, and junction quality.

### 6.2. Surface Cleaning

Surface cleaning is a critical step in CZTSSe device fabrication because the absorber surface after selenization often contains secondary phases, off-stoichiometric surface layers, oxides, residual chalcogen species, or segregated Cu-, Zn-, and Sn-rich compounds. These surface phases can strongly affect CdS buffer-layer nucleation, absorber/buffer band alignment, interface recombination, shunt resistance, and fill factor [7,8,9,25,26,27,38]. Therefore, surface cleaning should not be treated as a cosmetic post-treatment; it is an interface-engineering step that determines whether the bulk absorber quality can be translated into device performance. More importantly, surface cleaning should be judged by device physics rather than by chemical removal alone. A cleaning treatment is beneficial only if it improves the electronic quality of the absorber/buffer interface without damaging absorber stoichiometry, roughening the surface excessively, exposing a defective subsurface layer, or creating unfavorable band alignment. Removing conductive Cu_2−x_Se is useful because it can suppress shunting and improve fill factor, but over-etching may deplete Cu near the surface, enrich Zn- or Sn-related species, change CdS nucleation, increase interface defect density, and worsen $V_{OC}$. Therefore, the success of a surface-cleaning step should be verified by coupled chemical, structural, and electronic diagnostics, including Raman/XPS/TEM/EDS for phase and composition, UPS or XPS/UPS for band alignment, PL/TRPL or temperature-dependent J–V for interface recombination, and statistically meaningful J–V/EQE analysis.

**Figure 21:**
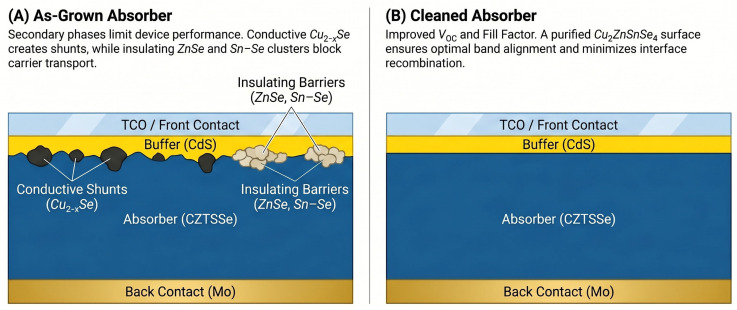
Surface-cleaning and secondary-phase-removal strategies for CZTSSe. KCN, bromine-methanol, ammonium sulfide, and emerging cyanide-free treatments differ in selectivity, safety, damage risk, and compatibility with subsequent buffer-layer deposition. AI-assisted schematic; scientific basis supported by Refs. [25,26,27,28,38,39].

Common surface-cleaning approaches include KCN etching, bromine–methanol etching, ammonium sulfide treatment, and emerging electrochemical or cyclic-voltammetry (CV)-based etching. Each method has different selectivity, safety, and scalability concerns. KCN etching has been widely used to remove conductive Cu_2−x_Se phases from Cu-rich CIGS and CZTSe/CZTSSe surfaces [25]. The main surface-cleaning and secondary-phase-removal approaches are summarized in Figure 21.

KCN etching has historically been one of the most common surface treatments in chalcopyrite and kesterite thin-film solar cells. It is particularly effective for removing Cu-rich conductive phases such as Cu_2−x_Se, which can create shunting pathways and reduce device fill factor [25]. However, KCN treatment should not be described as universally beneficial. Its value depends on selective removal of conductive Cu-rich phases without excessive attack of the kesterite surface. Over-etching can alter the near-surface Cu/(Zn+Sn) ratio, increase surface roughness, expose defective subsurface regions, modify CdS nucleation, and change absorber/buffer band alignment. Therefore, KCN-treated absorbers should be evaluated not only by disappearance of Cu_2−x_Se signatures, but also by $V_{OC}$, FF, shunt resistance, EQE, PL/TRPL, surface composition, and interface band alignment.

Bromine–methanol etching is another widely discussed chemical treatment. Compared with KCN, Br_2_–methanol is more aggressive and can remove a broader range of surface phases or surface oxides. This broader etching ability can be useful when the surface contains mixed Cu-, Zn-, Sn-, or oxide-rich residues, but it also creates a higher risk of nonselective surface dissolution, roughening, thickness loss, and stoichiometric imbalance. Therefore, Br_2_–methanol treatment should be justified only when the improvement in interface electronic quality outweighs the risk of surface damage. Its effect should be verified by surface composition analysis, roughness/thickness measurements, CdS nucleation behavior, band-alignment measurements, and device statistics.

Ammonium sulfide treatment offers a milder route for surface cleaning and passivation. Buffière et al. investigated liquid and vapor-based ammonium sulfide treatments for Cu_2_ZnSnSe_4_ thin films and showed that the treatment modifies surface properties and device behavior [27]. From a device-physics perspective, the intended benefit is not only chemical cleaning but also reduction of interface states, improved CdS nucleation, and more favorable absorber/buffer band alignment. However, ammonium sulfide may also introduce sulfur-rich surface chemistry, unstable residues, or surface composition changes if poorly controlled. Therefore, its effectiveness should be judged by interface recombination, $V_{OC}$, FF, band alignment, PL/TRPL, and surface chemical stability rather than by surface cleaning alone.

Electrochemical or CV etching is a promising cyanide-free concept because it may provide potential-controlled selectivity. In principle, conductive Cu_2−x_Se or other electrochemically active surface phases can be oxidized and dissolved within a selected potential window while the bulk CZTSSe absorber remains relatively protected. Thiourea-based, sulfide-based, thiosulfate-based, or other cyanide-free chemical treatments may also be attractive for safer processing. However, replacing KCN is meaningful only if the alternative treatment preserves or improves device-relevant interface quality. Cyanide-free cleaning must therefore be evaluated for phase selectivity, surface stoichiometry retention, roughness control, absorber/buffer band alignment, interface recombination, waste compatibility, and large-area reproducibility. A safer etchant that damages the interface or worsens *V*_OC_ is not a successful replacement. The selectivity, benefits, and safety limits of surface-cleaning strategies are compared in Table 18.

The choice of surface-cleaning method should depend on the dominant surface phase and the intended interface design. If conductive Cu_2−x_Se is the main problem, KCN or carefully designed electrochemical etching may be effective.

Electrochemical or CV etching is a promising cyanide-free approach for selectively removing electrochemically active surface secondary phases such as Cu_2−x_Se. However, its selectivity, reproducibility, surface-damage control, and scalability require systematic validation before it can be considered a mature alternative to KCN.

Surface etching can remove specific secondary phases, but its effect on device performance depends on selectivity, surface stoichiometry, roughness, residual defects, and compatibility with subsequent buffer-layer deposition.

Critical summary: Surface cleaning can remove harmful secondary phases and improve junction formation, but it can also damage the absorber, alter stoichiometry, or create nonrepresentative surface chemistry. Cleaning protocols should therefore be evaluated by before/after Raman, XPS/SIMS, PL/TRPL, band alignment, and full device metrics rather than by etching selectivity alone.

### 6.3. Interface and Back-Contact Control

**Figure 22:**
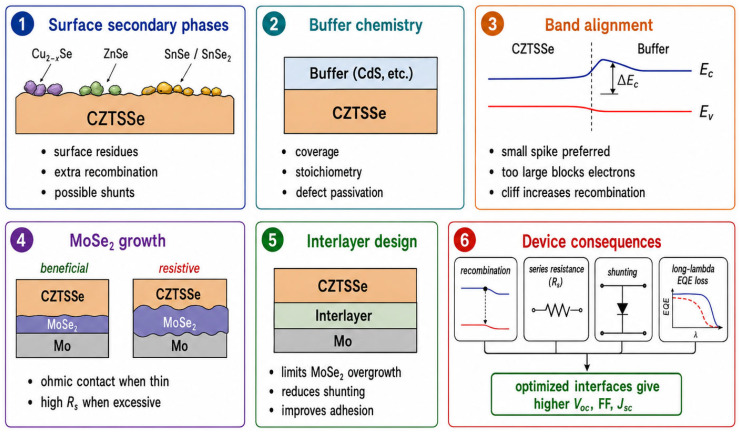
Front-interface and back-contact engineering in CZTSSe solar cells. Surface secondary phases, CdS/buffer chemistry, conduction-band offset, MoSe_2_ growth, and interlayer design collectively determine junction recombination, series resistance, shunting, and long-wavelength carrier collection. AI-assisted schematic; scientific basis supported by Refs. [29,30,31,37,38,39].

Interface and back-contact engineering are essential for translating the improved microstructure of CZTSSe absorbers into high photovoltaic performance. Even when the absorber is dense, large-grained, and apparently phase-pure, device efficiency can still be limited by recombination at the CdS/CZTSSe heterojunction, unfavorable conduction-band alignment, residual surface secondary phases, excessive MoSe_2_ growth, and poor back-contact selectivity [7,8,9,25,26,27,28,38]. The front-interface and back-contact issues are summarized in Figure 22.

The front heterojunction is commonly formed by chemical bath deposition of CdS on CZTSSe. Although CdS is widely used because it forms conformal coverage and has suitable chemical compatibility with many chalcogenide absorbers, the CdS/CZTSSe interface is also a major recombination site.

Conduction-band alignment is another critical issue. An ideal buffer/absorber interface should provide a small positive conduction-band offset, often described as a mild “spike,” which can suppress interface recombination without severely blocking electron transport. A large positive spike can hinder carrier collection, whereas a negative “cliff” can increase interface recombination and reduce VOC [9,39].

Residual surface secondary phases strongly influence interface quality. Conductive Cu_2−x_Se residues can form shunting pathways or locally short the junction, reducing shunt resistance and fill factor.

Back-contact control is equally important. During selenization, Mo reacts with Se to form MoSe_2_ at the absorber/Mo interface.

Several back-contact barrier or intermediate layers have been explored to regulate MoSe_2_ formation and improve rear-interface quality. Thin MoO_x_ layers, TiN, carbon-based interlayers, graphene oxide/reduced graphene oxide, MoS_2_/MoSe_2_-type transition-metal dichalcogenide layers, and other high-work-function or diffusion-control layers have been proposed to reduce excessive MoSe_2_ growth, improve wettability, passivate defects, or tune back-contact band alignment [29,30,31].

Alternative buffer layers are also important for reducing front-interface recombination and replacing Cd-containing layers. Zn(O,S), In_2_S_3_, Zn_1−x_Sn_x_Oᵧ, TiO_2_, and hybrid buffer structures have been investigated to tune conduction-band alignment, improve transparency, and reduce toxicity [39]. The front-interface and back-contact issues are summarized in Table 19.

Surface cleaning can improve CdS/CZTSSe junction formation by removing conductive or blocking secondary phases, but the final interface quality also depends on band alignment, surface stoichiometry, CdS deposition chemistry, and interface defect density.

MoSe_2_ formation is thickness- and morphology-dependent. A thin MoSe_2_ layer may improve contact formation and adhesion, whereas excessive MoSe_2_ growth can increase series resistance, weaken adhesion, and enhance rear-interface recombination.

Alternative buffer layers such as Zn(O,S), In_2_S_3_, Zn_1−x_Sn_x_Oᵧ, and TiO_2_ may improve band alignment or reduce Cd use, but their effectiveness depends on coverage, interface chemistry, conduction-band offset, defect density, and processing compatibility.

Critical summary: Interface and back-contact engineering are decisive because even a high-quality bulk absorber can fail through CdS/CZTSSe recombination or excessive MoSe_2_/back-contact resistance. The strength of interface modification is direct impact on VOC and FF, but the weakness is that local chemistry is difficult to decouple from bulk changes; temperature-dependent J-V, Suns-VOC, EQE, PL/TRPL, and cross-sectional analysis are required.

## 7. Carrier Transport and Device Performance

The ultimate purpose of microstructural, compositional, and interface control in colloidal CIGS and CZTSSe absorbers is to improve carrier transport and photovoltaic-device performance. However, transport in polycrystalline chalcogenide thin films is governed by several coupled factors, including grain size, grain-boundary chemistry, residual porosity, carbon contamination, secondary phases, point defects, band tailing, carrier concentration, and interface recombination [7,8,9,15,24,38].

### 7.1. Transport Mechanisms

Carrier transport in colloidal CIGS and CZTSSe absorbers is commonly evaluated using Hall-effect measurements, resistivity, carrier concentration, mobility, current–voltage behavior, and external quantum efficiency. Among these, Hall mobility is often used as an indicator of the absorber’s electrical quality because it reflects how easily majority carriers move through the polycrystalline film.

**Figure 23:**
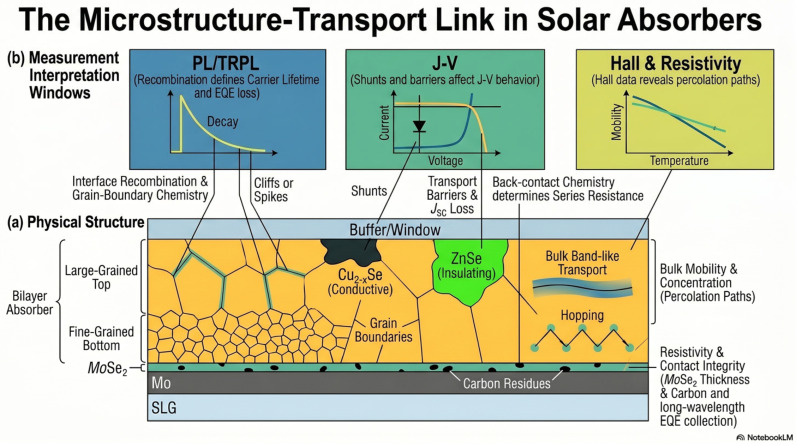
Transport interpretation in colloidal CIGS/CZTSSe absorbers. Hall mobility, resistivity, carrier concentration, J-V behavior, EQE, PL/TRPL, and temperature-dependent measurements must be correlated with grain-boundary chemistry, secondary phases, and interface losses. AI-assisted schematic; scientific basis supported by Refs. [21,36,45,46,47,48].

However, Hall mobility must be interpreted carefully. An increase in mobility does not automatically prove that the transport mechanism has changed to true band-like transport. The relationship between transport parameters and device interpretation is summarized in Figure 23.

Resistivity is another important parameter, but it must be analyzed together with carrier concentration and mobility. A lower resistivity may result from improved mobility, increased carrier concentration, conductive secondary phases, or shunting pathways.

Grain-boundary scattering is particularly important in nanoparticle-derived CZTSSe because the precursor film initially consists of many small particles separated by ligands and pores. If chalcogenization does not fully coalesce the particles into large grains, the final absorber retains a high density of grain boundaries.

Carrier collection depends not only on bulk transport but also on the spatial distribution of recombination and blocking regions through the film thickness. In CZTSSe absorbers with a bilayer microstructure, the large-grained upper layer may transport carriers more effectively, whereas the fine-grained bottom layer near the Mo contact may limit long-wavelength carrier collection.

The absorber/buffer and absorber/back-contact interfaces also strongly affect transport measurements. At the front interface, unfavorable conduction-band alignment, surface defects, ZnSe residues, or Cu_2−x_Se shunts can dominate V_OC_, fill factor, and leakage behavior [7,8,9,38,39]. Therefore, transport claims should be tested against device-physics signatures rather than morphology alone. A processing change that truly reduces recombination should lower the band-gap-normalized voltage loss, Eg/q-V_OC_, increase the implied V_OC_ or quasi-Fermi-level splitting measured by Suns-VOC or absolute photoluminescence, reduce the diode ideality factor toward values consistent with reduced trap-assisted recombination, and improve the temperature-dependent J-V activation energy toward the optical band gap. The interpretation of transport parameters is summarized in Table 20.

A rigorous discussion of carrier transport should therefore distinguish between microstructural transport improvement and fundamental transport-mechanism change. Processing improvements such as ligand exchange, pressure-assisted selenization, Ag alloying, and surface cleaning can increase Hall mobility, reduce resistivity, and improve carrier collection.

For colloidal CZTSSe absorbers, the most important transport challenge is to connect microstructure with device performance. Hall mobility and resistivity provide useful absorber-level information, but they must be correlated with grain-boundary chemistry, secondary-phase distribution, defect density, and interface quality.

Pressure-assisted selenization and Ag substitution can improve Hall mobility and reduce resistivity by enhancing grain growth, reducing grain-boundary scattering, and improving carrier percolation. However, band-like transport should only be claimed if temperature-dependent mobility measurements support delocalized carrier transport.

The increase in Hall mobility suggests reduced grain-boundary scattering or improved carrier percolation, but it does not prove that grain-boundary barriers are eliminated. Grain-boundary chemistry, residual secondary phases, and temperature-dependent transport should be analyzed to clarify the mechanism.

Lower resistivity may indicate improved transport, but it may also arise from conductive secondary phases such as Cu_2−x_Se. Therefore, resistivity should be interpreted together with Raman, XPS, TEM/EDS, Hall, and device data.

Critical summary: Transport analysis provides a bridge between processing defects and device losses, but single parameters such as mobility, resistivity, or grain size are not diagnostic by themselves. Reliable interpretation requires temperature-dependent measurements, secondary-phase checks, carrier-density analysis, and correlation with recombination-sensitive optical/electrical probes.

### 7.2. Device Metrics

Device metrics provide the most direct test of whether colloidal processing, green-body engineering, reactive chalcogenization, defect control, and interface optimization have successfully produced a functional photovoltaic absorber. However, in a review manuscript, device-performance comparison must be handled carefully.

For colloidal and solution-processed CIGS/CIGSSe devices, published results show that non-vacuum processing can reach efficiencies above 12–16%, depending on precursor chemistry, alkali incorporation, selenization, and post-treatment. For example, sulfide nanocrystal inks with low-temperature Na incorporation produced approximately 12% efficient Cu(In,Ga)(S,Se)_2_ devices, demonstrating the importance of alkali-assisted microstructural and electronic optimization [45]. Later nanoparticle-ink CIGSSe devices reached 15% total-area efficiency, among the highest reported for solution-processed CIGSSe absorbers [46].

**Figure 24:**
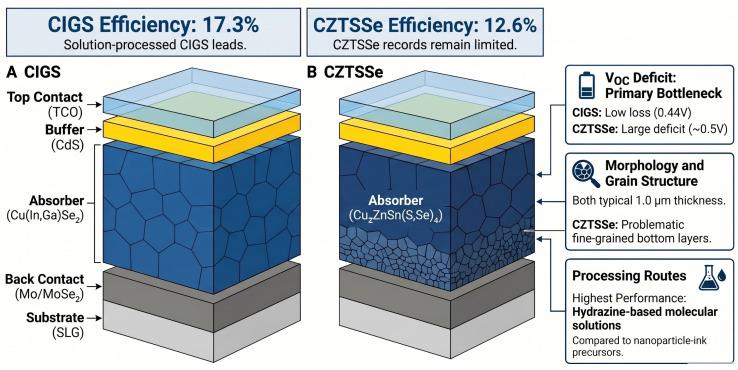
Representative device-performance benchmarking for colloidal and solution-processed CIGS/CZTSSe solar cells. Efficiency, VOC deficit, JSC, FF, absorber thickness, grain size, processing route, phase diagnostics, and depth-profile information should be compared together rather than as isolated device metrics. AI-assisted schematic; scientific basis supported by Refs. [1,2,5,6,36,42,45,46,47,48,56].

For CZTSSe, the performance comparison should distinguish between nanoparticle ink routes, molecular precursor routes, and more recent defect- or selenization-controlled kesterite devices. Nanoparticle-derived CZTSSe devices fabricated from selenized CZTS nanocrystal inks have reached approximately 7.2–9.0% efficiency, whereas hydrazine-based molecular precursor processing produced the 12.6% CZTSSe benchmark and later alloying or selenization-control studies have pushed kesterite efficiencies beyond 13–14% [6,36,37,47,48,53]. These comparisons show that the central problem is not simply whether the absorber can be densified. Rather, the key question is whether the processing route simultaneously controls residual carbon, grain-size distribution, secondary phases, S/Se and Sn depth profiles, MoSe_2_ thickness, band tailing, and front/back-interface recombination. Representative performance benchmarks are summarized in Figure 24, Table 21 and Table 22, while the diagnostic evidence that should accompany such comparisons is summarized in Table 23. Table 23 converts the green-body framework into an evidence-based comparison matrix. Rather than treating schematic processing steps as sufficient explanation, the table identifies the experimental quantities required to verify whether a reported improvement arises from better precursor packing, cleaner burnout, more uniform chalcogen transport, suppressed secondary phases, improved back-contact reaction, or reduced electronic recombination. This approach also highlights an important limitation of the present literature: many high-efficiency reports provide final J–V parameters and SEM morphology, but do not report the complete diagnostic set needed to separate green-body, chalcogenization, interface, and intrinsic-defect contributions.

The comparison highlights several important points. First, solution-processed and nanoparticle-derived CIGSSe have reached higher efficiencies than nanoparticle-derived CZTSSe, reflecting the greater defect tolerance of the chalcopyrite lattice and the more mature control of alkali incorporation, crystallization, and interfaces [45,46,55,56]. Second, the 12.6% hydrazine-processed CZTSSe benchmark shows that high current density and reasonable fill factor are possible in kesterite devices, but the remaining VOC deficit demonstrates that dense morphology does not remove Cu/Zn disorder, band tailing, Sn-related defects, or interface recombination [6,36]. Third, nanoparticle-derived CZTSSe remains especially sensitive to precursor-state variables because residual carbon, incomplete coalescence, fine-grained bottom layers, and buried secondary phases can survive chalcogenization and degrade FF, JSC, EQE, and device reproducibility [15,38,48].

Therefore, device metrics should be interpreted together with diagnostic evidence. A high JSC may indicate good absorption and carrier collection, but it does not guarantee low recombination. A higher FF may indicate improved transport, but it can also be limited by shunts, series resistance, or a thick MoSe_2_ layer. Likewise, large grains should be considered beneficial only when Raman mapping, XPS or SIMS depth profiles, TEM/EELS, PL yield, TRPL lifetime, EQE, admittance spectroscopy, DLTS or TAS, Suns-VOC, and temperature-dependent J-V measurements show that secondary phases, residual carbon, band tailing, interface recombination, and carrier-collection losses have also been reduced.

Benchmarking should be restricted to published, verifiable device metrics and should clearly identify missing or non-comparable quantities. In the literature, photovoltaic parameters are usually reported more consistently than carbon concentration, SIMS depth profiles, grain-size distributions, or MoSe_2_ thickness. This reporting imbalance is itself an important conclusion: future colloidal CIGS/CZTSSe studies should report process windows, precursor solid loading, ligand-removal chemistry, residual carbon levels, grain-size statistics, Raman phase maps, SIMS/GDOES depth profiles, MoSe_2_ thickness, and full J-V/EQE statistics in the same study.

An improved microstructure can enhance carrier transport and collection, but high device efficiency also requires reduced VOC deficit, suppressed secondary phases, controlled band alignment, low interface recombination, and optimized series/shunt resistance. The purpose of the green-body framework is therefore not to replace device physics with processing schematics, but to define which precursor and microstructural variables should be measured quantitatively and correlated with final photovoltaic losses.

Critical summary: Device metrics are necessary because they quantify whether processing changes improve VOC, JSC, FF, and efficiency, but they are insufficient without mechanistic diagnostics. Higher efficiency can arise from many competing changes, so benchmark tables must be interpreted together with EQE, Suns-VOC, temperature-dependent J-V, PL/TRPL, and chemical/microstructural evidence.

### 7.3. Remaining Bottlenecks

Despite substantial progress in precursor chemistry, chalcogenization control, alloying, surface cleaning, and interface engineering, CZTSSe solar cells still suffer from several persistent bottlenecks that limit their performance relative to CIGS. The most important limitations are the large open-circuit-voltage deficit, interface recombination, band tailing, secondary-phase formation, fine-grained bottom-layer formation, and poor process reproducibility [7,8,9,24,36,38,48].

**Figure 25:**
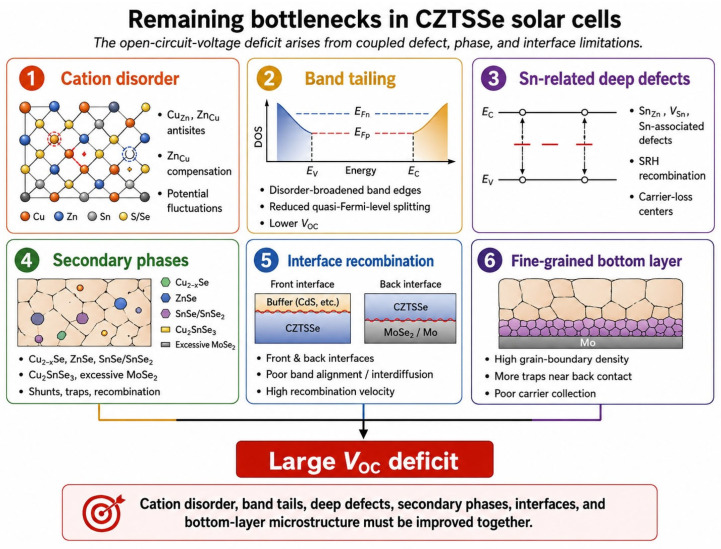
Remaining bottlenecks in CZTSSe solar cells. The open-circuit-voltage deficit is linked to cation disorder, band tailing, Sn-related deep defects, secondary phases, interface recombination, and fine-grained bottom-layer formation. AI-assisted schematic; scientific basis supported by Refs. [7,8,9,25,28,36,37,38,39].

The most widely recognized bottleneck in CZTSSe is the V_OC_ deficit. Although CZTSSe has a suitable band gap and strong optical absorption, the measured V_OC_ remains much lower than expected from its band gap. This loss should be discussed quantitatively as Eg/q-V_OC_, or more rigorously by comparing the measured V_OC_ with the quasi-Fermi-level splitting inferred from calibrated PL or Suns-V_OC_. If the activation energy extracted from temperature-dependent J-V is substantially below the optical band gap, interface or dominant junction recombination is implicated; if the PL spectrum shows a large Stokes shift, broad low-energy emission, low absolute PL yield, or shortened TRPL lifetime, band tailing and nonradiative bulk recombination are likely contributors. The remaining CZTSSe bottlenecks are summarized in Figure 25.

Interface recombination is another major limitation. The front CdS/CZTSSe interface is sensitive to absorber surface composition, surface secondary phases, band alignment, Cd diffusion, and chemical-bath deposition conditions.

Band tailing remains one of the most difficult intrinsic problems in CZTSSe. Because Cu^+^ and Zn^2+^ have similar ionic radii and can exchange crystallographic sites, Cu/Zn disorder is energetically favorable.

Secondary phases also continue to limit performance and reproducibility. Cu_2−x_Se can create conductive shunts, ZnSe can form blocking layers at grain boundaries or interfaces, SnSe_2_ and Cu_2_SnSe_3_ can act as recombination-active phases, and excessive MoSe_2_ can increase back-contact resistance [25,38].

The fine-grained bottom layer is a particularly important bottleneck for nanoparticle-derived CZTSSe absorbers. This layer near the Mo back contact typically contains small grains, high grain-boundary density, residual porosity, possible carbon residues, secondary phases, and MoSe_2_-related interface reactions.

**Figure 26:**
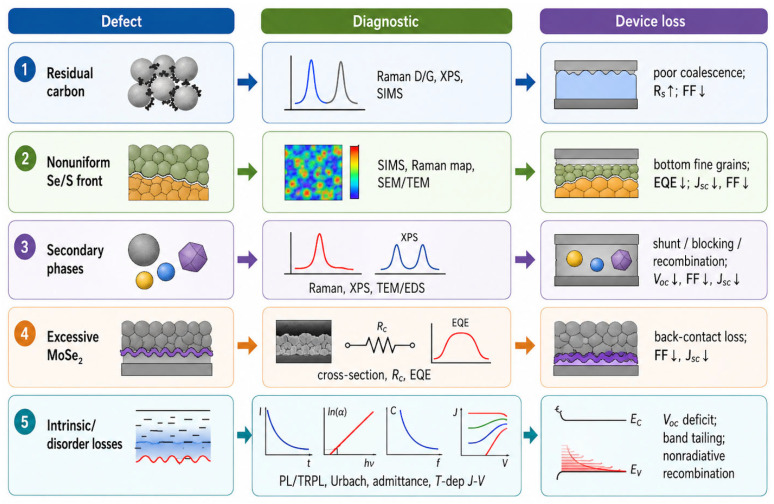
Processing-defect-to-device-loss flowchart for colloidal CIGS/CZTSSe absorbers. The figure visually summarizes the core relationships distilled from Table 24. Representative processing or absorber defects are linked to their most relevant diagnostic signatures and final device consequences. Examples include: ① Residual carbon → Raman D/G bands, XPS C 1s, SIMS carbon depth profile, TEM/EELS → suppressed particle coalescence, increased series resistance, lower FF, and enhanced recombination. ② Nonuniform Se/S reaction-front propagation → SIMS S/Se depth profiles, Raman mapping, cross-sectional SEM/TEM → fine-grained bottom layer, incomplete conversion, lower long-wavelength EQE, reduced Jsc and FF. ③ Secondary phases (e.g., Cu_2−x_Se, ZnSe, Sn-related phases) → Raman, XPS, TEM/EDS, selective etching, depth profiling → shunting, carrier blocking, recombination, reduced Voc/FF/Jsc. ④ Excessive or nonuniform MoSe_2_/back-contact reaction → cross-sectional Raman, SEM/TEM, contact-resistance analysis, EQE → increased back-contact resistance, adhesion loss, poor rear collection, reduced FF and Jsc. ⑤ Intrinsic or disorder-related losses remaining after microstructural optimization → PL/TRPL, Urbach energy, admittance spectroscopy, temperature-dependent J–V → Voc deficit, band tailing, and nonradiative recombination. AI-assisted schematic; scientific basis supported by Refs. [20,21,25,26,27,28,29,30,31,32,34,35,36,37,38,39,49,50].

Finally, reproducibility remains a serious challenge for CZTSSe. Because the kesterite phase has a narrow stability window, small variations in precursor composition, ligand residue, film thickness, Se amount, heating rate, furnace geometry, gas flow, pressure, and substrate condition can strongly affect phase evolution and device performance [7,8,9,24]. To avoid a purely qualitative discussion, the processing origin of each loss mechanism should be tied to measurable electrical, optical, chemical, and microstructural signatures. The remaining bottlenecks and required diagnostic approaches are summarized in Table 24. The core relationships in this matrix are also converted into the visual pathway map in Figure 26 to improve rapid interpretation of the processing-defect-diagnostic-device-loss chain.

The fine-grained bottom layer is an important limitation in nanoparticle-derived CZTSSe absorbers, but it is part of a broader set of coupled bottlenecks, including VOC deficit, Cu/Zn disorder, band tailing, secondary phases, interface recombination, MoSe_2_ control, and reproducibility. Its device relevance should be judged from long-wavelength EQE, bias-dependent EQE, series-resistance analysis, and cross-sectional chemical mapping rather than from SEM contrast alone.

Improved grain growth can enhance carrier transport, but it does not by itself eliminate Cu/Zn disorder, Sn-related deep defects, band tailing, secondary phases, or interface recombination. Device improvement requires simultaneous control of microstructure, defect chemistry, and interfaces, verified by a consistent set of electrical and spectroscopic signatures: reduced Eg/q-VOC, lower ideality factor, higher Suns-VOC or quasi-Fermi-level splitting, higher PL yield, longer TRPL lifetime, weaker sub-band-gap EQE tailing, and fewer phase or composition gradients in Raman, XPS/SIMS, and TEM/EELS analysis.

Process reproducibility should be demonstrated statistically through multiple devices and batches, with full reporting of precursor composition, ink formulation, film thickness, selenization conditions, and device-area definition.

Critical summary: The remaining bottlenecks in CZTSSe are coupled rather than isolated: VOC deficit, band tailing, secondary phases, fine-grained bottom layers, interface recombination, and reproducibility often reinforce one another. The most promising path is therefore not a single additive or annealing change, but an evidence chain linking precursor state, reaction pathway, defect spectrum, and complete device response.

## 8. Outlook and Future Directions

Future progress in colloidal CIGS and CZTSSe photovoltaics requires moving beyond empirical optimization toward mechanism-guided processing. For CZTSSe in particular, the remaining barriers—V_OC_ deficit, Cu/Zn disorder, band tailing, secondary phases, fine-grained bottom layers, and poor reproducibility—are too strongly coupled to be solved by a single processing change. Therefore, the next stage of development should focus on four high-impact directions: operando characterization, interface passivation, alloying and defect control, and scalable manufacturing.

### 8.1. Operando Characterization of Reactive Chalcogenization

**Figure 27:**
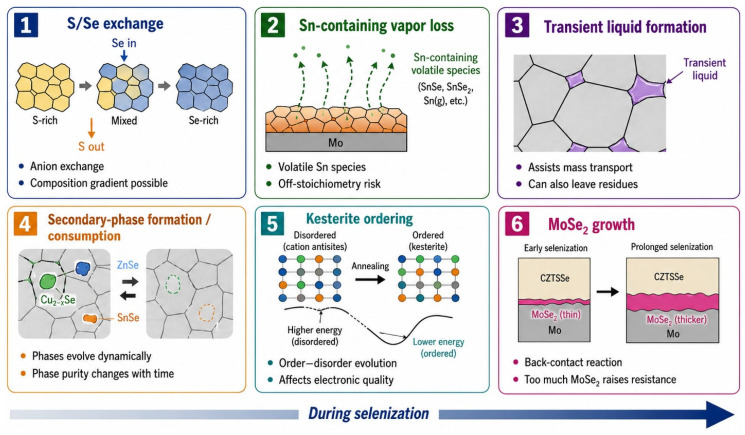
Operando characterization of reactive chalcogenization in nanoparticle-derived CZTSSe absorbers. During selenization, the precursor undergoes coupled transient processes including S/Se exchange, Sn-containing vapor loss, transient liquid formation, secondary-phase evolution, kesterite ordering, grain coarsening, and MoSe_2_ growth. Because many intermediates disappear or redistribute during cooling, operando probes such as in situ XRD, Raman spectroscopy, synchrotron scattering, vapor-phase analysis, and interrupted-quench experiments are needed to link reaction pathways with final absorber quality and device losses. AI-assisted schematic; scientific basis supported by Refs. [23,24,32,35,49,50].

A major limitation in current colloidal CZTSSe processing is that the most critical reactions occur transiently during selenization and are therefore difficult to reconstruct from post-mortem characterization alone. During heating, the nanoparticle-derived precursor is not converted into the final absorber through a single equilibrium reaction. Instead, it undergoes a coupled sequence of S/Se exchange, Sn-containing vapor formation and loss, transient liquid or liquid-like phase formation, secondary-phase formation and consumption, cation redistribution, kesterite ordering, grain coarsening, and MoSe_2_ growth at the back contact (Figure 27). Many of these intermediate states may disappear during cooling or be redistributed during the final stage of annealing; therefore, ex situ XRD, Raman spectroscopy, SEM, or TEM performed only after selenization can identify the final microstructure but cannot reliably reveal the reaction pathway.

This limitation is particularly important for CZTSSe because device performance is governed not only by the final grain size and apparent phase purity, but also by the thermal history of defect formation, volatile-species loss, secondary-phase evolution, and interface reactions. For example, temporary Cu–Se or Cu–Sn–Se liquid phases may promote grain growth, but residual Cu_2−x_Se or Cu_2_SnSe_3_ can introduce shunting paths or nonselective recombination. Similarly, Sn loss during high-temperature selenization can shift the absorber away from the narrow kesterite phase-stability window and promote deep defects, band tailing, and *V*_OC_ loss. Excessive MoSe_2_ growth may improve local contact formation at early stages but later increase series resistance or weaken back-contact adhesion. Therefore, operando or time-resolved characterization is essential for distinguishing beneficial transient reaction pathways from detrimental residual phases.

Future studies should combine in situ or operando XRD, Raman spectroscopy, optical/thermal monitoring, synchrotron-based scattering, and vapor-phase analysis with interrupted-quench experiments and depth-resolved post-mortem characterization. Such integrated analysis would clarify when S/Se exchange begins, when Sn-containing species volatilize, when transient liquids appear and disappear, how secondary phases are consumed, how kesterite ordering develops, and when MoSe_2_ becomes excessive. This information is needed to design selenization profiles that control chalcogen chemical potential, suppress Sn loss, manage transient-liquid-assisted coarsening, and minimize interface degradation.

Critical summary: Operando characterization is powerful because it can reveal transient phases, vapor loss, and reaction sequences that disappear after cooling. Its weakness is experimental complexity and limited spatial statistics; the best studies will combine in situ probes with interrupted-quench, depth profiling, and final device correlation.

### 8.2. Interface Passivation and Contact Selectivity

The second priority is the development of interface-passivation and contact-selective strategies. Even if colloidal CZTSSe processing successfully improves grain growth, film density, and phase uniformity, device efficiency will remain limited if recombination at the front CdS/CZTSSe junction and the rear CZTSSe/Mo contact is not suppressed. Therefore, future interface engineering should be evaluated not only by chemical compatibility or improved morphology, but also by its ability to reduce Eg/q-V_OC_, lower the ideality factor, raise Suns-V_OC_ and calibrated PL yield, improve EQE response, suppress interface recombination in temperature-dependent J-V, and maintain stable carrier extraction.

At the front interface, CdS remains the most widely used buffer layer, but its band alignment with CZTSSe is not always ideal. Depending on the absorber composition, surface termination, S/(S + Se) ratio, and near-surface defect chemistry, the CdS/CZTSSe junction may form unfavorable band offsets that enhance interface recombination or impede carrier collection. Alternative buffer layers, such as Zn(O,S), ZnSnO, In_2_S_3_, TiO_2_, or other cadmium-free materials, should therefore be explored more systematically. However, these layers should not be judged only by environmental benefit or simple replacement of CdS. Their conduction-band alignment, interface defect density, chemical stability, processing temperature, and compatibility with CZTSSe surface chemistry must be carefully optimized.

At the rear interface, the CZTSSe/Mo contact also requires more deliberate control. A thin MoSe_2_ layer may improve adhesion and provide a useful transition layer, but excessive or nonuniform MoSe_2_ growth can increase series resistance, weaken adhesion, and promote back-interface recombination. Rear-interface passivation layers or contact modifiers should therefore be designed to suppress excessive MoSe_2_ formation, reduce recombination at the back contact, and preserve hole selectivity. Possible strategies include ultrathin diffusion-control layers, alkali-containing interlayers, carbon-based interlayers, MoO_x_/MoSe_2_ control layers, or alternative back-contact architectures.

Overall, interface engineering in CZTSSe should move from empirical buffer replacement toward device-physics-guided contact design. The most effective passivation strategy will be one that simultaneously controls band alignment, suppresses interface defects and secondary phases, improves carrier selectivity, and maintains low-resistance charge extraction at both the front and rear contacts.

Critical summary: Interface passivation and selective contacts offer a direct route to reducing recombination and improving VOC, but they can introduce barriers, parasitic absorption, or chemical incompatibility. Their value must be judged by activation energy, ideality factor, Suns-VOC, EQE, PL/TRPL, and durability, not only by initial efficiency gain.

### 8.3. Alloying and Defect Control Linked to V_OC_ Deficit

Alloying strategies must be evaluated by how effectively they reduce V_OC_ deficit, band tailing, and recombination, rather than only by whether they increase grain size. Future studies should therefore avoid using grain size or film density as indirect evidence for reduced Eg/q-V_OC_. In CZTSSe, improved ceramic processing can remove extrinsic barriers to carrier collection, but the dominant voltage loss is also governed by intrinsic kesterite defect thermodynamics. Effective alloying or post-treatment strategies must be evaluated by direct evidence of reduced Cu/Zn disorder, weaker band tailing, suppressed Sn-related deep defects, improved quasi-Fermi-level splitting, and reduced nonradiative recombination. This requires correlating ceramic-processing variables with PL yield, TRPL lifetime, Suns-V_OC_, temperature-dependent J-V, ideality-factor analysis, admittance spectroscopy, DLTS or TAS, Raman order-disorder analysis, SIMS depth profiling, and interface-resolved recombination measurements. Ag, Ge, Sb, Li, and alkali elements such as Na, K, Rb, and Cs can modify crystallization, defect chemistry, carrier concentration, grain-boundary passivation, and interface behavior [37,47,51,52,53,54]. However, these additives should be treated as defect-control tools with trade-offs.

Ag substitution may reduce Cu/Zn disorder, expand the lattice, improve grain growth, and enhance Hall mobility, but excessive Ag can widen the band gap, lower J_SC_, alter carrier concentration, or form Ag-rich secondary phases [47,51]. Ge can modify Sn-related defect chemistry and promote crystallization, but it may also alter the band gap or segregate if poorly controlled [37,52].

Future alloying studies should therefore include defect-sensitive diagnostics: temperature-dependent PL, absolute PL or calibrated PL yield, TRPL, Suns-V_OC_, Urbach-energy analysis from EQE or absorption, admittance spectroscopy, DLTS or TAS, temperature-dependent J-V, SIMS dopant profiling, and atomically resolved STEM/EDS, TEM/EELS, or APT where possible. This is essential because a dopant that improves morphology may still worsen electronic quality if it produces compensation, secondary phases, or unfavorable band alignment.

Critical summary: Alloying strategies are most meaningful when they are explicitly linked to the VOC deficit rather than treated as empirical recipes. The key weakness is mechanistic ambiguity; Ag, Ge, Sb, Li, and alkali elements can alter structure, chemistry, and interfaces at the same time, so each claim requires defect-sensitive and depth-resolved evidence.

### 8.4. Scalable Manufacturing and Environmentally Safer Processing

The final challenge is scalable manufacturing. Many high-efficiency colloidal devices are still fabricated by spin coating, small-area doctor blading, or laboratory-scale selenization.

**Figure 28:**
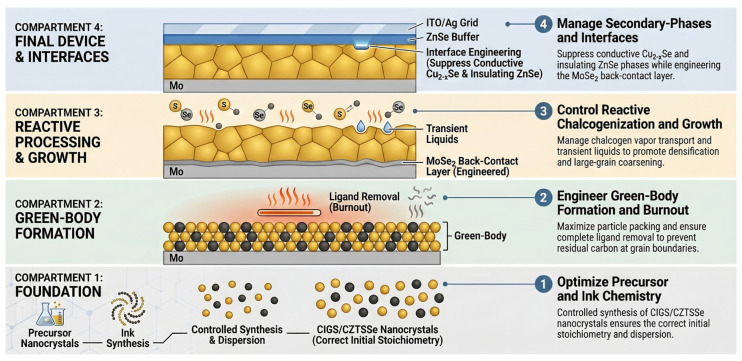
Integrated processing map for colloidal CIGS/CZTSSe solar cells. Device improvement requires simultaneous optimization of precursor chemistry, green-body formation, binder burnout, reactive chalcogenization, secondary-phase control, defect chemistry, and interface engineering. AI-assisted schematic; scientific basis supported by Refs. [15,18,20,21,23,24,28,34,36,37,38,39,40,41,42,43,44,45,46,47,48].

However, scalable manufacturing introduces new ceramic-processing challenges. Drying stress, coffee-ring effects, nonuniform solvent evaporation, thickness gradients, and coating defects become more difficult to control over large areas. Roll-to-roll drying also requires careful management of solvent evaporation rate, substrate temperature, web speed, and intermediate consolidation. Pressure-assisted selenization may be useful at the laboratory scale, but its scalability must be assessed in terms of reactor design, Se utilization, pressure uniformity, throughput, safety, and compatibility with large-area substrates. The integrated processing map is summarized in Figure 28.

Environmental and safety issues must also be addressed. Hydrazine-based molecular routes have demonstrated high performance, but hydrazine toxicity limits industrial attractiveness [6,42]. Future research priorities are summarized in Table 25.

Future work should focus on mechanism-guided control of the entire processing pathway, including transient phase evolution, Se/Sn transport, defect chemistry, interface passivation, and scalable coating.

Ag, Ge, Sb, Li, and alkali treatments should be evaluated by their ability to reduce V_OC_ deficit, band tailing, recombination, and secondary-phase formation, rather than only by their effect on grain size.

CV etching is a promising cyanide-free surface-cleaning approach, but its selectivity, reproducibility, electrolyte compatibility, waste management, and large-area scalability require systematic validation. In addition, cyanide-free cleaning should be judged by device physics: it must reduce shunting and interface recombination, preserve near-surface stoichiometry, maintain favorable absorber/buffer band alignment, and improve $V_{OC}$, FF, and EQE reproducibly.

Selenization and sulfurization also impose manufacturing constraints. Se vapor, H_2_S/H_2_Se-containing atmospheres, volatile Sn species, and CdS buffer layers require engineering controls, gas scrubbing, and waste-management protocols. Large-area coating methods such as slot-die coating, doctor blading, spray coating, screen printing, and inkjet printing must therefore be coupled with in-line thickness mapping, drying control, defect inspection, and statistically meaningful device testing. These considerations are essential if ceramic-style green-body engineering is to translate from small-area champion cells to reproducible modules.

**Figure 29:**
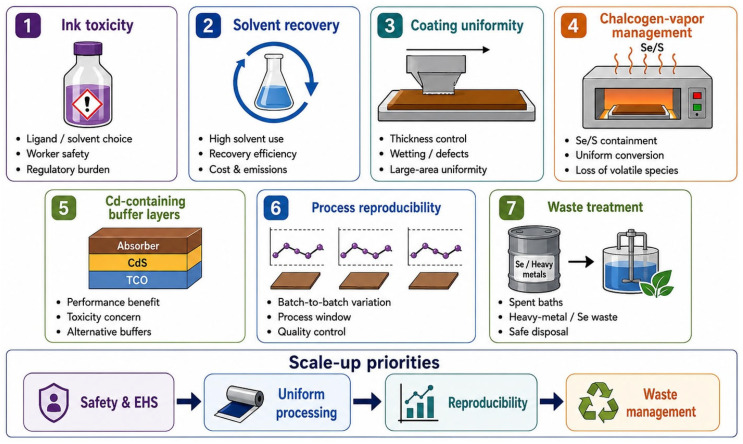
Scalability and manufacturability considerations for colloidal chalcogenide absorbers. Ink toxicity, solvent recovery, coating uniformity, chalcogen-vapor management, Cd-containing buffer layers, process reproducibility, and waste treatment determine the practical viability of non-vacuum processing. AI-assisted schematic; scientific basis supported by Refs. [15,21,22,42,43,44,45,46,47,48,57,58,59,60,61,62].

For practical manufacturing, colloidal chalcogenide absorbers must be evaluated not only by champion efficiency but also by solvent safety, waste handling, large-area coating uniformity, and process reproducibility. Hydrazine-based molecular inks have demonstrated the power of solution chemistry, but hydrazine toxicity and handling requirements are severe barriers. The manufacturability and safety issues are summarized in Figure 29.

Critical summary: Scalable and safer processing is essential for the practical relevance of colloidal CIGS/CZTSSe absorbers. The strength of non-vacuum inks is manufacturability, but the weakness is process sensitivity across large areas; industrially useful routes must demonstrate coating uniformity, low-toxicity chemistry, reproducible chalcogenization, waste control, and statistically stable module-scale performance.

### 8.5. Cost Structure and Energy Payback Time

Although this review focuses mainly on precursor processing, green-body evolution, reactive chalcogenization, and defect-limited photovoltaic performance, cost and energy payback time (EPBT) are essential criteria for evaluating the practical relevance of CIGS and CZTSSe technologies. The cost advantage of a photovoltaic absorber cannot be judged only from elemental abundance. In a complete module, the absorber layer usually represents only one part of the total cost. Substrate or glass, encapsulation, transparent conductive oxide, Mo back contact, buffer/window layers, metallization, scribing and interconnection, module integration, yield loss, capital depreciation, process energy, and operational lifetime can all make major contributions to the final cost per watt. Therefore, the earth-abundant Cu–Zn–Sn–S/Se chemistry of CZTSSe reduces exposure to In and Ga supply constraints, but it does not automatically guarantee lower module cost unless comparable efficiency, manufacturing yield, throughput, and stability are achieved [57,58,59].

A useful comparison is to separate material availability from area-related manufacturing cost, as summarized in Table 26. A useful comparison is to separate material availability from area-related manufacturing cost. CIGS benefits from relatively high thin-film efficiency, established monolithic integration, and more mature module processing, although the use of In and Ga introduces absorber-material supply and price considerations. CZTSSe replaces In and Ga with more abundant Zn and Sn, which is attractive for large-scale deployment. However, the present efficiency gap, large VOC deficit, secondary-phase sensitivity, Sn volatility, MoSe_2_/contact control, and interface recombination increase the required module area per watt and may offset the lower absorber-material cost. Thus, cost should be considered as a coupled materials–processing–performance metric rather than as an absorber-composition metric alone [57,58,59].

Energy payback time provides a complementary metric because it combines materials selection, process temperature, module efficiency, lifetime, manufacturing energy, and local solar irradiation into a single energy-balance parameter. EPBT is commonly defined as the cumulative primary energy demand required to manufacture, deploy, and, where relevant, replace the PV system divided by the annual primary-energy-equivalent electricity generated by that system [59,60,61]. Lower-temperature solution processing, thin absorbers, and high-throughput coating can reduce the cumulative energy demand. However, low device efficiency, poor manufacturing yield, short lifetime, or frequent replacement increases EPBT because more module area and more embodied energy are required per delivered watt.

The ceramic-processing framework discussed in this review is therefore directly relevant to cost and EPBT, even though it is not itself a cost model. Improved green-body packing, cleaner ligand removal, controlled drying, uniform chalcogenization, optimized transient-liquid-assisted coarsening, suppressed secondary phases, controlled MoSe_2_ thickness, and stable front/back interfaces can increase the usable watt output per unit area and reduce yield loss. Conversely, residual carbon, cracking, fine-grained bottom layers, nonuniform S/Se reaction fronts, conductive Cu_2−x_Se, blocking ZnSe or Sn-related phases, excessive MoSe_2_, and unstable interfaces reduce VOC, FF, JSC, device reproducibility, and lifetime. These losses increase the module area and process input required per watt, thereby weakening both cost competitiveness and EPBT.

From this perspective, CZTSSe remains economically and energetically promising because it avoids the In/Ga resource constraint and is compatible in principle with solution or nanoparticle-based processing. However, its present advantage is potential rather than fully demonstrated at commercial scale. CZTSSe should therefore not be presented simply as a cheaper version of CIGS. It should be evaluated as a distinct earth-abundant thin-film platform whose cost and EPBT competitiveness require simultaneous progress in absorber crystallization, defect thermodynamics, interface passivation, manufacturing yield, module efficiency, and long-term operational stability [57,58,59].

Critical summary: Cost and EPBT analyses are useful because they force CIGS and CZTSSe processing to be evaluated at the module and energy-balance levels rather than only by absorber chemistry. The strength of CZTSSe is its earth-abundant elemental basis and potential compatibility with scalable solutions or nanoparticle processing, but its weakness is that lower efficiency, V_OC_ deficit, yield loss, uncertain lifetime, and interface instability can erase the apparent raw-material advantage. Therefore, future cost claims should be tied to demonstrated module efficiency, process yield, stability, embodied energy, and EPBT rather than to precursor cost alone.

## 9. Conclusions

Colloidal CIGS and CZTSSe absorbers are best interpreted as constrained ceramic-like precursor films only within a defined analogy. Ceramic concepts such as green-body formation, constrained shrinkage, burnout, and consolidation help identify measurable precursor-state variables, whereas absorber formation itself is governed by chalcogenide-specific reactive chalcogenization: Se/S transport, S/Se exchange, volatile Sn chemistry, transient liquid or vapor intermediates, secondary-phase redistribution, and Mo-interface reaction. This separation clarifies why particle packing, residual organics, capillary stress, pore structure, substrate constraint, and chalcogen chemical potential all strongly affect final device quality.

CIGS and CZTSSe should not be treated as interchangeable absorber systems. CIGS benefits from greater defect tolerance but still requires controlled Ga grading, alkali incorporation, MoSe_2_ thickness, and absorber/buffer alignment. CZTSSe is more severely constrained by Cu/Zn disorder, Sn volatility, secondary phases, band tailing, and interface recombination.

For nanoparticle-derived absorbers, microstructural improvement alone is not sufficient. Large grains and apparent densification must be correlated quantitatively with device metrics and diagnostics, including Eg/q-V_OC_, ideality factor, temperature-dependent J-V activation energy, Suns-V_OC_ or quasi-Fermi-level splitting, FF, J_SC_, EQE and EQE tailing, Raman phase maps, XPS/SIMS/TEM/EELS evidence for secondary phases and residual carbon, MoSe_2_ thickness/continuity, PL yield, TRPL lifetime, admittance/DLTS/TAS evidence for recombination-active defects, Hall evidence for transport, and statistically meaningful device metrics.

The key processing challenge is to control the coupled pathway from precursor chemistry to device interfaces via clean ligand removal, stable ink rheology, crack-free drying, uniform Se/S transport, suppression of Cu_2−x_Se/ZnSe/Sn-Se phases, passivated grain boundaries, and controlled front/back contacts.

Future relevant research should combine ceramic-processing analysis with photovoltaic diagnostics. Operando reaction studies, quantitative green-body metrics, safer hydrazine-free and cyanide-free chemistries, scalable coating trials, and mechanism-guided alloying/passivation will be essential for turning colloidal CIGS/CZTSSe processing into reproducible, high-performance manufacturing. Surface-cleaning strategies should be accepted as beneficial only when they improve interface electronic quality, as evidenced by lower Eg/q-V_OC_, lower ideality factor, better band alignment, higher Suns-V_OC_ or PL yield, improved FF, and stable EQE, without damaging absorber stoichiometry or introducing new surface defects.

## Figures and Tables

**Table 1:** Key differences between CIGS and CZTSSe thin-film absorbers.

Item	CIGS	CZTSSe	Refs.
Crystal structure	Chalcopyrite	Kesterite	[1,2,5,6,7,8,9]
Representative composition	Cu(In,Ga)Se_2_	Cu_2_ZnSn(S,Se)_4_	[1,2,5,6,7,8,9]
Main advantage	High efficiency and relatively high defect tolerance	Earth-abundant and lower-toxicity constituent elements	[1,2,5,6,9]
Band-gap control	Mainly through Ga/(In + Ga) ratio	Mainly through S/(S + Se) ratio and partial cation substitution	[5,6,7,8,9,36]
Key performance limitation	In/Ga scarcity, Ga grading, alkali control, interface recombination	Large Voc deficit, Cu/Zn disorder, band tailing, secondary phases	[5,7,8,9,36]
Major processing challenge	Composition grading, Na/K diffusion, crystallization, MoSe_2_ control	Narrow phase-stability window, Sn loss, Cu/Zn disorder, secondary-phase suppression	[5,7,8,9,24,36]
Typical secondary/interface concerns	MoSe_2_ thickness, Ga gradients, absorber/buffer interface	Cu_2−x_Se, ZnSe, SnSe_2_, Cu_2_SnSe_3_, excessive MoSe_2_	[25,26,27,28,29,37,38]
Main characterization needs	Composition depth profiling, alkali analysis, interface characterization	Raman, XPS, SIMS, TEM/EDS, defect spectroscopy, recombination analysis	[7,8,9,28,37,38]
Colloidal-processing concern	Achieving dense films with controlled Ga and alkali distribution	Achieving dense films while controlling disorder, Sn volatility, and secondary phases	[21,22,34,36,40,41,42,43,44,45,46,47,48]

**Table 2:** Processing-to-device loss map for colloidal CIGS/CZTSSe absorbers.

Processing Defect	Microstructural Consequence	Electronic Consequence	Typical Device Signature	Refs.
Residual carbon	Poor particle coalescence; carbon-rich boundaries	Recombination sites and high series resistance	Low FF, low J_SC_, weak long-wavelength EQE	[21,22,34]
Mud cracking or pinholes	Discontinuous absorber and shunting paths	Local junction failure and leakage	Low shunt resistance, scattered J-V statistics	[16,17,20,21,34]
Cu_2−x_Se secondary phase	Conductive surface/GB inclusions	Shunting and nonselective recombination	Low V_OC_ and low FF	[25,26,28]
ZnSe or Sn-related phases	Insulating/blocking regions or off-stoichiometry	Transport barrier, deep defects, and band tails	Reduced J_SC_, FF, and high V_OC_ deficit	[28,37,38]
Excessive MoSe_2_/back reaction	Thick or nonuniform back-contact layer	High series resistance and back recombination	Low FF; poor long-wavelength response	[29,30,31]
Fine-grained bottom layer	High GB density near Mo; incomplete coarsening	Back-interface recombination and carrier trapping	Poor EQE near band edge; low V_OC_	[21,32,48]

**Table 3:** Comparison of major precursor synthesis routes for colloidal and solution-processed CIGS/CZTSSe absorbers.

Route	Advantages	Limitations	Processing Relevance	Refs.
Hot injection	Produces monodisperse and crystalline particles; good control of nucleation and growth	Poor scalability; difficult heat and mass transfer during rapid injection; reactivity mismatch among multication precursors	Useful for fundamental nanocrystal studies and controlled particle synthesis	[10,13,40,41]
Heating-up	Better scalability; gradual phase evolution; improved compositional control in multinary systems	Sensitive to ligand/solvent chemistry, heating rate, and precursor reactivity; may yield broader size distributions	Promising for scalable nanoparticle ink preparation	[10,11]
Solvothermal/hydrothermal	Simple, relatively low cost, closed-system synthesis; possible use of less expensive precursors	Larger particles, broader size distribution, agglomeration, less controllable surface chemistry	Useful for powder-based or hybrid approaches, but often requires post-treatment	[12,43]
Molecular precursor	Excellent compositional mixing at molecular scale; can form dense films	Toxic solvents in some systems; impurity and residue control required	High-performance solution processing, especially if clean conversion is achieved	[15,36,42,44]
Hybrid particle/molecular route	Combines particle control with molecular-scale pore filling and compositional adjustment	More complex chemistry; possible impurity residues and nonuniform conversion	Attractive for improving green density and reactive sintering behavior	[22,36,40,41,42,43,44]

**Table 4:** Effects of ligand chemistry and carbon residues on colloidal CIGS/CZTSSe processing.

Processing Factor	Beneficial Role	Potential Problem	Characterization Method	Refs.
Oleylamine	Stabilizes nanocrystals; controls nucleation and growth	Difficult to remove; leaves carbonaceous residues	FTIR, TGA/DSC, XPS	[15,34,40,41]
1-Hexanethiol exchange	Replaces long-chain OLA; reduces organic spacing; improves particle contact	May induce aggregation, sulfur-rich surface chemistry, or incomplete exchange	FTIR, TGA, zeta potential, dispersion stability	[21,22,34]
m-Xylene washing	Removes free or weakly bound OLA; reduces organic loading	Does not necessarily replace strongly bound ligands	FTIR, elemental analysis	[21,22,34]
Residual carbon	None for device operation; may temporarily preserve film shape during burnout	Blocks diffusion, inhibits grain growth, increases resistance and recombination	Raman, SIMS, XPS, TEM/EELS	[21,22,34]
Optimized ligand removal	Promotes densification, grain growth, and carrier transport	Requires balance with ink stability and coating quality	SEM, Hall measurement, J–V, EQE	[21,34,45,46]

**Table 5:** Ink-formulation and green-body-packing factors affecting colloidal CIGS/CZTSSe absorbers [18,20,21,34,40,41,45,46,48].

Factor	Beneficial Effect When Optimized	Problem When Poorly Controlled	Recommended Characterization	Refs.
Solid loading	Improves green density and reduces shrinkage	Too low: high porosity; too high: aggregation and poor coating	Mass fraction, viscosity, SEM cross-section	[18,21,22,34]
Viscosity	Controls film thickness and leveling	Too low: dewetting/coffee-ring effect; too high: roughness and trapped pores	Rheometry, coating-thickness mapping	[18,20,21]
Dispersion stability	Prevents sedimentation and agglomeration	Agglomerates create local pores and secondary phases	DLS, zeta potential, sedimentation test	[18,21,22,34]
Wetting on Mo substrate	Produces continuous and pinhole-free coating	Poor wetting causes pinholes, islands, and shunting	Contact angle, optical microscopy, SEM	[21,22,34]
Drying rate	Controls capillary stress and shrinkage uniformity	Rapid drying causes cracking and thickness gradients	In situ drying observation, optical microscopy	[18,20,21]
Bimodal particle packing	Increases packing density and reduces constrained shrinkage	May cause local compositional or conversion inhomogeneity	SEM, EDS mapping, Raman mapping	[21,34,45,46]
Multilayer coating	Builds required absorber thickness and fills defects	Stress accumulation and interlayer delamination	Cross-sectional SEM, profilometry	[21,22,34]
Green-body homogeneity	Promotes uniform chalcogenization and grain growth	Nonuniform reaction front and residual porosity	SEM, Raman, SIMS, EQE correlation	[21,22,34]

**Table 6:** Origins and consequences of constrained-shrinkage defects in colloidal CIGS/CZTSSe films.

Defect	Primary Origin	Consequence During Chalcogenization	Device-Level Effect	Possible Mitigation	Refs.
Mud cracks	Tensile stress from constrained drying	Nonuniform Se/S transport; local secondary phases	Shunting, poor junction uniformity	Higher green density, slower drying, thinner coating layers	[16,17,20,21]
Residual porosity	Low packing density, trapped solvent, incomplete particle rearrangement	Incomplete densification and poor grain coalescence	Lower mobility, increased recombination	Optimized solid loading, bimodal packing, staged drying	[16,17,20,34]
Delamination	Poor adhesion to Mo; high shrinkage stress	Poor back-contact formation; uneven MoSe_2_ growth	High series resistance, device failure	Substrate cleaning, wetting control, gradual drying	[16,17,20]
Thickness nonuniformity	Poor leveling, high viscosity, rapid evaporation	Nonuniform reaction front and grain size	Spatially variable current collection	Rheology control, coating optimization	[21,22,34]
Stress-induced pore opening	Organic burnout and constrained shrinkage	Persistent voids and interrupted grain growth	Reduced fill factor and carrier collection	Ligand reduction, staged burnout, pre-sintering	[21,34]

**Table 7:** Characterization methods for organic burnout and residual carbon in colloidal CIGS/CZTSSe films.

Method	Information Obtained	Strength	Limitation	Refs.
TGA	Mass loss from solvent, ligand, and additive removal	Quantifies organic content and decomposition temperature	Powder data may not represent thin-film behavior	[15,21,34]
DSC	Endothermic/exothermic events during drying, decomposition, or reaction	Identifies decomposition and reaction events	Requires careful assignment of overlapping processes	[21,22,34]
FTIR	Organic functional groups such as C–H, N–H, S–H, C–N	Tracks ligand exchange and ligand removal	Less sensitive to buried or carbonized residues	[21,34]
Raman	Amorphous/graphitic carbon D and G bands; chalcogenide phase information	Links residual carbon with phase formation and secondary phases	Surface sensitivity and peak overlap must be considered	[21,34]
XPS	Surface carbon, nitrogen, sulfur, selenium, and metal chemical states	Identifies surface residues and chemical bonding	Limited probing depth; not sufficient for buried carbon	[21,22,34]
SIMS	Depth distribution of carbon, Na, K, S, Se, and other elements	Reveals buried carbon and interface contamination	Quantification requires standards and careful calibration	[21,34]
TEM/EELS	Local carbon at grain boundaries or interfaces	Direct nanoscale evidence	Limited sampling area and complex preparation	[21,22,34]

**Table 8:** Possible roles and cautions for pressure-assisted pre-sintering of nanoparticle-derived CIGS/CZTSSe precursor films.

Proposed Effect	Possible Mechanism	Expected Benefit	Required Evidence	Caution	Refs.
Improved particle contact	Ligand removal, particle rearrangement, partial necking	More uniform reaction and grain growth during chalcogenization	SEM/TEM, reduced pore volume, improved conductivity	Do not assume direct pressure bonding without evidence	[16,17,18,21,34]
Reduced cracking	Stronger green network, slower volatile release, reduced shrinkage gradient	Fewer mud cracks and delamination defects	Optical microscopy, SEM crack-density analysis	Crack reduction may arise from thermal history, not pressure alone	[16,17,18,34]
Partial compaction, when directly measured	Pore reduction, local particle rearrangement, mild consolidation	Higher green density and improved continuity	Thickness change, porosity analysis, density increase, profilometry, cross-sectional SEM/TEM	Use improved contact or continuity if direct compaction evidence is absent	[16,17,21,34]
Reduced organic residues	Staged removal of solvent and ligands before selenization	Less carbon at particle contacts and grain boundaries	TGA/DSC, FTIR, Raman, XPS, SIMS	Improved morphology alone is insufficient proof	[18,21,34]
Improved chalcogenization uniformity	Cleaner particle contacts and more connected precursor network	More uniform Se/S transport and grain coarsening	Raman mapping, EDS/SIMS depth profiles	Must distinguish from effects of later selenization	[16,17,21,34]
Better device performance	Reduced cracks, porosity, carbon residues, and interfacial defects	Higher carrier mobility, FF, and current collection	Hall, J–V, EQE, PL/TRPL	Device improvement may involve multiple coupled factors	[21,34]

**Table 9:** Coupled processes during reactive chalcogenization of colloidal CIGS and CZTSSe films.

Process	Role During Chalcogenization	Possible Benefit	Possible Problem	Refs.
Chalcogen vapor transport	Supplies Se or S to the precursor film	Enables conversion to CIGS or CZTSSe	Nonuniform penetration can cause bilayer microstructure	[14,23,24,32]
S/Se exchange	Replaces S with Se or adjusts S/(S + Se) ratio	Tunes band gap and lattice parameter	Incomplete exchange causes compositional gradients	[23,24,32]
Phase transformation	Converts binary/ternary precursors into chalcopyrite or kesterite phase	Forms functional absorber	Intermediate or secondary phases may remain	[23,24]
Transient liquid formation, if demonstrated	Enhances mass transport only when a liquid/liquid-like intermediate is present and consumed	Promotes large-grained dense films	Residual Cu-rich or Cu-Sn-Se phases may cause shunting; large grains alone are insufficient evidence	[24,32]
Organic-residue removal	Cleans particle contacts during heating	Improves coalescence and transport	Carbon residues inhibit grain growth and increase recombination	[23,24,32]
Interface reaction	Forms MoSe_2_ or other back-contact phases	Thin MoSe_2_ may assist contact formation	Excessive MoSe_2_ increases series resistance	[21,23,24]
Secondary-phase redistribution	Segregates excess Cu, Zn, Sn, or chalcogen species	May relieve local off-stoichiometry	Cu_2−x_Se, ZnSe, SnSe_2_, or Cu_2_SnSe_3_ degrade devices	[24,32]

**Table 10:** Distinct roles of pressure during pressure-assisted selenization.

Effect	Role During Selenization	Possible Benefit	Caution	Refs.
Chemical effect	Maintains high Se activity and suppresses volatile loss	Promotes complete chalcogenization, reduces Sn loss, stabilizes CIGS/CZTSSe formation	Should be linked to Se chemical potential and phase stability, not simply density	[24,32]
Transport effect	Modifies Se penetration, vapor transport, and reaction-front movement	Improves through-thickness conversion and may suppress fine-grained bottom layers	Strongly depends on pore structure, carbon residue, and precursor packing	[24,32]
Liquid-phase effect	Stabilizes or modifies Se-rich, Cu–Se, Cu–Sn–Se, or multicomponent transient liquids	Enhances mass transport, grain coarsening, and densification	Residual Cu-rich liquid may generate shunting secondary phases	[24,32]
Volatile-species control	Suppresses evaporation of Sn- or Se-containing species	Maintains stoichiometry and reduces decomposition	Excess pressure/Se activity may enhance MoSe_2_ growth or surface segregation	[24]
Possible mechanical effect, not assumed compaction	May reduce pore expansion, crack opening, or film disruption	Improves film continuity and reduces defect formation	Not equivalent to hot pressing unless load transfer, thickness change, density increase, or porosity reduction is demonstrated	[24,32]
Interface effect	Affects MoSe_2_ formation and back-contact reaction	Thin MoSe_2_ may improve contact; controlled reaction may improve adhesion	Excessive MoSe_2_ increases series resistance and may weaken adhesion	[24,32]

**Table 11:** Possible transient liquid phases and their effects during selenization.

Possible Liquid Phase	Possible Origin	Beneficial Role	Potential Risk	Evidence required Before Assigning Mechanism	Refs.
Se-rich liquid	Excess Se supply or local Se condensation	Enhances chalcogen transport and S/Se exchange	May promote excessive MoSe_2_ or surface segregation	Se activity control, in situ analysis	[23,24]
Cu–Se-related liquid	Cu-rich local regions during selenization	Promotes rapid grain growth and dissolution–reprecipitation	Residual Cu_2−x_Se can cause shunting	Raman, XPS, TEM/EDS, phase analysis	[23,24]
Sn-Se-related liquid or vapor-mediated species	Sn-containing intermediates under Se atmosphere	May assist phase transformation, redistribution, or volatile-mediated transport	Sn loss and off-stoichiometry	SIMS, EDS depth profiles, vapor analysis, quenched phases	[23,24,32]
Cu–Sn–Se liquid	Reaction between Cu-, Sn-, and Se-rich regions	Strong liquid-assisted grain coarsening	Cu_2_SnSe_3_ or Cu-rich residues may remain	Raman, TEM, quenched microstructure	[23,24,35]
Ag–Se-containing liquid	Ag substitution or Ag-containing precursors	May enhance liquid mobility and grain growth	Excess Ag phases, band-gap/current trade-off	Ag mapping, XRD/Raman, device metrics	[23,24,32]
Multicomponent chalcogenide liquid	Realistic mixture of Cu–Zn–Sn–S–Se ± dopants	Most likely reaction medium in CZTSSe	Composition difficult to control; secondary phases possible	Thermodynamic modeling, in situ XRD/Raman, TEM/EDS	[23,24,32]

**Table 12:** Possible causes of bilayer microstructure in nanoparticle-derived CZTSSe films.

Possible Cause	Mechanism	Expected Evidence	Device-Level Consequence	Refs.
Nonuniform Se penetration	Surface region reacts first and densifies, slowing Se transport to the bottom region	Se/S depth profile, cross-sectional Raman mapping, reaction-front morphology	Incomplete conversion and weak carrier collection near back contact	[21,32,48]
Residual carbon	Carbon residues block particle coalescence and liquid redistribution near bottom layer	Raman D/G bands, SIMS carbon profile, TEM/EELS at grain boundaries	High resistance, recombination-active boundaries, fine-grained morphology	[21,48]
Substrate constraint	Mo-coated glass restricts shrinkage and grain-boundary migration near interface	Cross-sectional SEM, residual pores, delamination/crack analysis	Poor densification and nonuniform microstructure	[23,32,48]
MoSe_2_ formation	Se reacts with Mo, modifying interface chemistry and local stress	Raman/TEM identification of MoSe_2_ thickness and morphology	Excessive series resistance, back-contact recombination, adhesion loss	[21,32,48]
Sn loss	Volatile Sn species shift local composition away from kesterite stability	Sn depth profile, XPS/EDS/SIMS, secondary-phase detection	ZnSe, Cu_2_SnSe_3_, SnSe_2_ formation and increased recombination	[29,30,31,48]
Alkali diffusion gradient	Na/K diffusion from soda-lime glass modifies crystallization and defect chemistry near back contact	SIMS Na/K depth profile	Nonuniform carrier concentration, grain growth, and passivation	[21,32,48]
Nonuniform transient liquid formation	Liquid-assisted grain growth occurs preferentially near Se-rich surface	Quenched microstructure, TEM/EDS, phase mapping	Large-grained top layer but persistent fine-grained bottom layer	[21,32,48]

**Table 13:** Comparison of phase-evolution issues in CIGS and CZTSSe absorbers.

Aspect	CIGS	CZTSSe	Refs.
Main structural phase	Chalcopyrite Cu(In,Ga)Se_2_	Kesterite Cu_2_ZnSn(S,Se)_4_	[5,7,8,9,36,42]
Main reaction pathway	Cu–Se/In–Se/Ga–Se intermediates → chalcopyrite CIGS	CZTS or mixed precursor → CZTSSe through S/Se exchange	[5,42]
Major compositional variable	Ga/(In + Ga) ratio	S/(S + Se), Cu/(Zn + Sn), Zn/Sn	[7,8,9,36]
Key phase-evolution issue	Ga incorporation and Ga depth grading	Sn volatility, secondary phases, and kesterite ordering	[5,24,42]
Alkali effect	Na/K strongly influence grain growth, passivation, and carrier density	Na/K may influence crystallization and defect chemistry, but effects are more complex	[7,8,9,24,36]
Main secondary/interface concern	MoSe_2_ thickness, Ga gradients, back-contact recombination	Cu_2−x_Se, ZnSe, SnSe_2_, Cu_2_SnSe_3_, excessive MoSe_2_	[5,36,42]
Characterization priority	Ga depth profile, alkali profile, chalcopyrite phase formation	Raman phase mapping, Sn retention, S/Se depth profile, defect/order analysis	[7,8,9,28,36]
Device implication	Proper Ga/alkali control improves Voc and carrier collection	Disorder and secondary phases cause Voc deficit and recombination	[1,2,5,36]

**Table 14:** Secondary phases in CZTSSe: origin, device effect, and detection limitations.

Phase	Likely Origin/Location	Electrical Effect	Useful Methods	Key Limitation	Refs.
Cu_2−x_Se	Cu-rich local regions; surface or grain boundaries	Conductive shunt; nonselective recombination	Raman, EDS/TEM, KCN response, XPS	XRD overlap and poor sensitivity to minor phases	[25,26,28]
ZnSe	Zn-rich regions; surface/back-contact vicinity	Insulating barrier; transport blocking	Raman, SIMS, TEM/EDS	Raman penetration depth and excitation wavelength affect detection	[28,37,38]
SnSe/SnSe_2_-related phases	Sn-rich or Se-rich local chemistry; incomplete reaction	Deep defects; off-stoichiometry; recombination	Raman, XPS, TEM/EDS	Surface sensitivity of XPS; local statistics in TEM	[28]
Cu_2_SnSe_3_	Cu-Sn-Se intermediates or decomposition products	Potential recombination/shunting depending on distribution	Raman, XRD with caution, TEM	Diffraction/Raman overlap with kesterite features	[28,33]
Excess MoSe_2_	Mo/Se reaction at back contact	Series resistance or beneficial ohmic contact depending on thickness	Cross-sectional SEM/TEM, XRD, Raman	Must correlate thickness/continuity with J-V/EQE	[29,30,31]

**Table 15:** Major secondary phases in CZTSSe absorbers, their typical locations, and device effects.

Phase	Typical Location	Main Effect	Characterization Requirement	Refs.
Cu_2−x_Se	Surface, grain boundary	Conductive shunt; reduced shunt resistance and fill factor	Raman, XPS, TEM/EDS, selective etching response	[25,26,28]
ZnSe	Absorber/buffer interface, grain boundary, bulk	Carrier-blocking barrier; reduced current collection	Raman, TEM/EDS, XPS, depth profiling	[28,37,38]
SnSe_2_/Sn–Se phases	Surface, bulk, intermediate reaction regions	Recombination, phase instability, indication of Sn imbalance	Raman, XPS, SIMS, EDS mapping	[28]
Cu_2_SnSe_3_	Bulk, intermediate reaction region	Recombination center; incomplete kesterite formation	Raman, TEM/EDS, XRD with caution	[28,33]
MoSe_2_	Back contact	Beneficial if thin; resistive and mechanically harmful if excessive	Cross-sectional Raman, TEM, SIMS/GDOES	[29,30,31]

**Table 16:** Major point defects and defect-related loss mechanisms in CZTSSe.

Defect/Mechanism	Defect Type or Origin	Main Effect	Processing Relevance	Refs.
V_Cu_	Shallow acceptor	Provides p-type conductivity	Favored under Cu-poor conditions	[7,8,9]
Cu_Zn_	Antisite acceptor	Contributes to Cu/Zn disorder and band tailing	Low formation energy due to Cu/Zn similarity	[7,8,9]
Zn_Cu_	Antisite donor	Causes compensation and potential fluctuation	Coupled with Cu_Zn_ disorder	[7,8,9]
[Cu_Zn_ + Zn_Cu_] disorder	Antisite defect cluster	Band-edge fluctuation and Urbach tailing	Not eliminated simply by Cu-poor/Zn-rich composition	[7,8,9,36]
Sn_Zn_	Deep donor	Recombination-active defect, Voc loss	Related to Sn activity, Sn volatility, and local stoichiometry	[7,8,9,36]
Cu_2−x_Se	Conductive secondary phase	Shunting and reduced fill factor	Suppressed by Cu-poor processing but requires phase control	[25,26,28]
ZnSe	Insulating secondary phase	Blocking barrier and reduced carrier collection	Can form under excessive Zn-rich conditions	[28,37,38]
Band tailing	Disorder-induced electronic effect	Reduces effective band gap and Voc	Requires cation-order and defect-control strategies	[7,8,9,36]
Voc deficit	Combined device-level loss	Lower open-circuit voltage than expected from band gap	Controlled by bulk defects, interfaces, and secondary phases	[7,8,9,24,36]

**Table 17:** Alloying and dopant strategies for CZTSSe absorbers.

Strategy	Possible Benefit	Major Risk	Required Characterization	Refs.
Ag substitution	Reduces Cu/Zn disorder; expands lattice; enhances grain growth; may improve Hall mobility	Excessive band-gap widening; reduced Jsc; Ag-rich secondary phases; nonuniform Ag distribution	XRD lattice shift, Raman, SIMS/EDS Ag mapping, Hall, PL, J–V/EQE	[47,51]
Ge alloying	Reduces Sn-related defect effects; promotes grain growth; may improve band alignment	Ge-rich phases; excessive band-gap change; nonuniform Ge incorporation	SIMS, TEM/EDS, PL, Raman, band-gap analysis	[52]
Sb treatment	Promotes crystallization; passivates bulk/interface defects; may improve bottom grain growth	Sb segregation; secondary phases; interface instability	XPS, SIMS, TEM/EDS, PL/TRPL, device analysis	[53]
Li doping	Tunes carrier concentration; may assist shallow acceptor formation; interacts with Ag alloying	Overcompensation; nonuniform Li distribution; possible secondary phases	SIMS Li profiling, Hall, C–V, PL, J–V	[47,54]
Na/K/Rb/Cs alkali treatment	Grain-boundary passivation; improved carrier density; reduced recombination	Excess alkali segregation; interface instability; nonuniform depth profile	SIMS, XPS, PL/TRPL, admittance spectroscopy, J–V/EQE	[47,51,52,53,54]

**Table 18:** Comparison of surface-cleaning strategies for CZTSSe absorbers.

Treatment	Main Target	Advantages	Risks/Limitations	Required Validation	Refs.
KCN etching	Cu_2−x_Se and Cu-rich conductive phases	Effective removal of conductive shunts; widely used in laboratory devices	Highly toxic; may alter Cu surface composition; limited industrial attractiveness	Raman, XPS, J–V shunt analysis, safety/process control	[25,26]
Br_2_–methanol etching	Multiple surface secondary phases	Strong etching ability; can remove broader surface contamination	Harsh chemistry; possible over-etching, roughening, and nonselective removal	SEM/AFM, XPS, Raman, thickness and composition control	[37,38]
Ammonium sulfide treatment	Surface oxides, Sn-related clusters, surface passivation	Milder than KCN; possible passivation effect; liquid or vapor treatment possible	Composition change, excessive surface modification, uncertain selectivity	XPS, Raman, PL/TRPL, CdS interface analysis	[27]
Electrochemical/CV etching	Electrochemically active conductive surface phases	Potential-controlled selectivity; reduced reliance on toxic cyanide; promising for Cu_2−x_Se removal	Requires validation of selectivity, reproducibility, electrolyte compatibility, and scale-up	CV curves, Raman mapping, XPS, ICP, SEM/AFM, device statistics	[25,26,27,37,38]

**Table 19:** Interface and back-contact issues in CZTSSe solar cells.

Region	Main Issue	Device Consequence	Engineering Strategy	Required Characterization	Refs.
CdS/CZTSSe front interface	Interface defects, surface disorder, Cd diffusion	Increased recombination, lower Voc	Surface cleaning, controlled CdS deposition, passivation	XPS, UPS, PL/TRPL, temperature-dependent J–V	[39]
Buffer/absorber band alignment	Cliff-like or excessive spike offset	Interface recombination or carrier blocking	Alternative buffers, surface composition control	UPS/IPES, XPS valence-band analysis, device modeling	[39]
Surface Cu_2−x_Se	Conductive surface or grain-boundary residue	Shunting, low fill factor	KCN, CV etching, selective surface cleaning	Raman, XPS, SEM, shunt resistance	[29]
Surface/interfacial ZnSe	Insulating blocking phase	Poor carrier collection, reduced Jsc	Stoichiometry control, optimized etching	Raman, TEM/EDS, XPS, EQE	[29,30,31]
MoSe_2_ back contact	Too thin: poor contact; too thick: high resistance	Series resistance, delamination, rear recombination	Selenization control, barrier/intermediate layers	Cross-sectional TEM, Raman, SIMS, GDOES	[30,31]
Back-contact barrier layer	Chemical instability or high contact resistance	Poor rear carrier extraction	MoO_x_, TiN, carbon, graphene, TMD layers	Contact resistance, TEM, stability tests	[30,31]
Fine-grained bottom layer	High grain-boundary density near Mo	Rear recombination, weak long-wavelength EQE	Improved Se penetration, carbon control, back-contact engineering	Cross-sectional SEM/TEM, EQE, SIMS	[29,30,31,39]

**Table 20:** Transport parameters and their interpretation in colloidal CIGS/CZTSSe absorbers.

Parameter	Useful Information	Possible Interpretation	Caution	Refs.
Hall mobility	Majority-carrier transport through absorber	Higher mobility may indicate larger grains, cleaner grain boundaries, improved percolation	Does not prove band-like transport without temperature-dependent mobility	[21,34,36]
Carrier concentration	Doping level and compensation state	Reflects VCu acceptors, donor compensation, alkali/dopant effects	Excessive carrier density may arise from conductive secondary phases	[7,8,9,36]
Resistivity	Combined effect of carrier density and mobility	Lower resistivity may indicate improved transport	Could also indicate Cu_2−x_Se shunting	[21,25,34]
Grain size	Microstructural coarsening and reduced boundary density	Larger grains may reduce scattering	Grain-boundary chemistry may still dominate recombination	[21,34,48]
EQE response	Wavelength-dependent carrier collection	Long-wavelength loss suggests rear-region or back-contact problems	Must be correlated with thickness and optical absorption	[21,29,36]
J–V behavior	Device-level transport and recombination	FF, series resistance, shunt resistance, Voc loss	Does not identify microscopic origin alone	[36,45,46,47,48,55,56]
Temperature-dependent mobility	Transport mechanism	Can distinguish activated, hopping, or band-like trends	Required before claiming band-like transport	[21,36]

**Table 21:** Representative published performance benchmarks for colloidal/solution-processed CIGS and CZTSSe devices.

Absorber/Process	Representative Efficiency	V_OC_ Issue	Processing Feature	Main Caution	Ref.
Certified (Ag,Cu)(In,Ga)Se_2_ record	23.64%	V_OC_ loss relatively lower than kesterite	Ag alloying and steep Ga back grading	Vacuum/high-control benchmark, not colloidal	[5]
Hydrazine solution-processed CIGS	15.2%	Good voltage for solution route	Molecular solution chemistry	Hydrazine toxicity limits manufacturability	[42]
Nanoparticle-ink CIGSSe	15.0% total area	Still below vacuum CIGS	Sulfide nanoparticle ink and selenization	Requires rigorous ink/selenization control	[46]
Sulfide-nanocrystal CIGSSe with Na	12%	Na improves defects and grain growth	Low-temperature Na incorporation	Scalability and reproducibility must be shown	[45]
CZTSSe record-class hydrazine route	12.6%	Large V_OC_ deficit remains	Cu-poor/Zn-rich kesterite processing	High grains alone do not solve defect losses	[36]
CZTSSe nanoparticle inks	9.0% total area	Strong V_OC_ and FF limitations	Selenized nanoparticle precursor	Secondary phases, carbon, and bottom fine grains critical	[48]

**Table 22:** Representative published device metrics for colloidal and solution-processed CIGS/CZTSSe solar cells.

Absorber	Route	Treatment	Voc	Jsc	FF	Efficiency	Main Limitation
Cu(In,Ga)(S,Se)_2_	Sulfide nanocrystal ink	Ligand exchange, Na incorporation, selenization	-	-	-	12.0%	Need controlled Na incorporation, grain growth, and recombination reduction [45]
Cu(In,Ga)(S,Se)_2_	Nanoparticle ink	Optimized nanoparticle ink processing and selenization	-	-	-	15.0% total-area	Still below vacuum CIGS; requires precise alkali and interface control [46]
Cu(In,Ga)(S,Se)_2_	Solution/atmospheric processing	K incorporation, optimized absorber processing	656 mV	33.61 mA cm^−2^	72.65%	16.02%	Alkali amount and absorber uniformity must be carefully controlled [55]
Cu(In,Ga)Se_2_	All-non-vacuum CIGS processing	Non-vacuum absorber and device fabrication	0.58 V	34.82 mA cm^−2^	69.60%	14.05%	Non-vacuum interface and absorber uniformity remain critical [56]
Cu_2_ZnSn(S,Se)_4_	CZTS nanoparticle ink	Selenization of nanocrystal ink	-	-	Not fully accessible in summary; see original paper	9.0% total-area; 9.8% active-area	Fine-grained regions, recombination, Voc deficit, and secondary phases [48]
Cu_2_ZnSn(S,Se)_4_	Hydrazine-based molecular solution	Pure solution processing, optimized absorber/device architecture	≈0.51 V	≈35.2 mA cm^−2^	≈69.8%	12.6%	Voc deficit remains large despite improved bulk quality [36]

**Table 23:** Evidence-based synthesis matrix for representative device studies and required diagnostic quantities. NR or non-comparable entries should not be filled by inference; they identify reporting gaps that must be closed by future studies.

Evidence Item	Reference	Quantitative/Device Data Used in This Review	Processing or Diagnostic Variable	Critical Interpretation
CIGSSe nanoparticle and solution benchmarks	[45,46,55,56]	Representative non-vacuum CIGSSe devices show efficiencies of 12.0–16.02%. For the K-doped solution CIGSSe example, reported values are V_OC_ = 656 mV, JSC = 33.61 mA cm^−2^, FF = 72.65%, and efficiency = 16.02% [55]. For the all-non-vacuum CIGS example, reported values are V_OC_ = 0.58 V, J_SC_ = 34.82 mA cm^−2^, FF = 69.60%, and efficiency = 14.05% [56].	Alkali incorporation, selenization conditions, absorber uniformity, Ga/In distribution, and front/back-interface control.	CIGSSe benefits from higher defect tolerance than CZTSSe, but high non-vacuum performance still requires quantitative control of alkali distribution, absorber-depth uniformity, interface reaction, and back-contact quality.
CZTSSe molecular benchmark	[6,36]	Hydrazine-based molecular precursor processing produced a 12.6% CZTSSe device with approximately V_OC_ = 0.51 V, J_SC_ = 35.2 mA cm^−2^, and FF = 69.8% [36].	Cu-poor/Zn-rich processing, molecular-scale compositional mixing, selenization control, and optimized device architecture.	High current density and fill factor are possible in molecularly processed CZTSSe, but the remaining VOC deficit shows that dense absorber formation and good crystallinity do not eliminate intrinsic kesterite disorder, band tailing, and recombination losses.
CZTSSe nanoparticle benchmark	[15,48]	Selenized nanoparticle inks have produced approximately 9.0% total-area and 9.8% active-area efficiency [48].	Nanocrystal synthesis, ligand removal, green-body packing, chalcogenization, grain coarsening, and secondary-phase control.	This is the most direct evidence base for the particulate green-body framework. The lower performance relative to molecular and more recent defect-controlled routes indicates that carbon removal, particle-contact coalescence, secondary-phase suppression, and interface control remain coupled limitations.
Residual carbon and burnout	[15,34,38,48]	Comparable carbon wt%, at%, or depth-integrated carbon values are not consistently tabulated across benchmark device studies.	TGA/DSC, FTIR, Raman D/G bands, XPS C 1s, SIMS carbon depth profiles, and TEM/EELS at grain boundaries or interfaces.	Improved morphology is not sufficient evidence for clean burnout. Carbon metrics should be correlated with FF, series resistance, EQE, carrier lifetime, and recombination diagnostics. NR entries identify a major reporting gap in nanoparticle-derived absorber studies.
Grain size and bilayer morphology	[15,32,37,48,53]	Representative reports frequently show large-grained upper layers and fine-grained bottom regions, but grain-size statistics and bottom-layer thickness are not always reported in comparable form.	Cross-sectional SEM/TEM, grain-size distribution, absorber thickness, fine-grained bottom-layer thickness, and interrupted or quenched chalcogenization analysis.	Large grains are beneficial only when accompanied by reduced secondary phases, cleaner interfaces, and improved carrier collection. A large-grained upper layer can coexist with a defective bottom layer that limits long-wavelength EQE, FF, and VOC.
Raman phase signatures	[25,38,48]	The relevant comparison is not a single kesterite peak position, but phase-resolved identification of CZTSSe, ZnSe, Cu_2−x_Se, Sn–Se phases, Cu_2_SnSe_3_, MoSe_2_, and carbon D/G bands where applicable.	Multi-wavelength Raman spectroscopy, surface and cross-sectional Raman mapping, selective etching, and correlation with SEM/TEM/EDS.	XRD-only phase-purity claims are weak for CZTSSe because secondary phases may be Raman-active, spatially localized, or buried. Raman mapping should be linked to device statistics and local microstructure rather than used only for qualitative phase confirmation.
SIMS and depth-profile evidence	[37,47,48,52,54]	Comparable SIMS profiles are not consistently available for all benchmark devices.	C, Na/K/Li/Ag/Ge/Sb, S/Se, Sn, and impurity depth profiles across the absorber thickness and Mo interface.	Depth profiles are essential for distinguishing beneficial alloying or passivation from segregation, buried carbon, Sn imbalance, S/Se gradients, and interface-localized secondary phases. The lack of standardized SIMS reporting limits cross-study comparison.
MoSe_2_ and back-contact evidence	[29,30,39]	The most relevant quantitative variable is MoSe_2_ thickness and continuity, together with its correlation with FF, series resistance, adhesion, and long-wavelength EQE.	Cross-sectional Raman, TEM, SEM, GDOES/SIMS, contact-resistance analysis, adhesion tests, and EQE.	A thin and continuous MoSe_2_ layer can improve back contact, whereas thick, discontinuous, or nonuniform MoSe_2_ can increase resistance, promote delamination, interrupt bottom-layer coarsening, and degrade carrier collection.

**Table 24:** Processing-defect-to-device-loss diagnostic matrix for CZTSSe solar cells.

Processing Defect or Loss Mechanism	Expected Measurable Signature	Primary Diagnostics	Interpretation for CZTSSe Devices	Refs.
Cu/Zn disorder, Sn-related deep defects, and band tailing	Large Eg/q-V_OC_; broad low-energy PL; large Stokes shift; low PL yield; shortened TRPL lifetime; broad sub-gap response	Band-gap/Eg analysis, calibrated PL, TRPL, EQE tail/Urbach analysis, admittance spectroscopy, DLTS or TAS, Raman order-disorder analysis	Identifies whether the voltage loss arises from intrinsic kesterite disorder, tail states, or deep recombination centers rather than from morphology alone.	[7,8,9,36]
Front CdS/CZTSSe interface defects, cliff-like band offset, surface ZnSe or Cu_2−x_Se	V_OC_ activation energy below Eg; high ideality factor; strong light-intensity dependence of V_OC_; low Suns-V_OC_ implied voltage; weak blue/near-surface EQE	Temperature-dependent J-V, Suns-V_OC_, light-intensity J-V, PL/TRPL after surface treatment, XPS/UPS band alignment, Raman/XPS surface analysis	Separates interface recombination and band-alignment losses from bulk-lifetime limitations.	[37,38,39]
Back-contact reaction, excessive or nonuniform MoSe_2_, fine-grained bottom layer	Reduced long-wavelength EQE; increased series resistance; lower FF; carrier-collection loss under weak bias; possible rear-interface recombination	Bias-dependent EQE, cross-sectional SEM/TEM, TEM/EELS, SIMS depth profiles, Raman mapping, J-V series-resistance analysis	Tests whether rear-interface chemistry and bottom-layer microstructure limit collection from the back side of the absorber.	[29,30,31]
Residual carbon from incomplete ligand exchange or burnout	Low mobility or high resistivity; reduced PL yield; short TRPL lifetime; poor FF; nonuniform device statistics	Raman D/G bands, XPS C 1s, SIMS carbon depth profiles, TEM/EELS at grain boundaries, Hall measurements, J-V statistics	Connects green-body organic removal directly to recombination-active boundaries and transport barriers.	[21,22,34]
Conductive Cu_2−x_Se, Cu-Sn-Se residues, or shunting secondary phases	Low shunt resistance; abnormal dark J-V leakage; low FF; spatially nonuniform photocurrent	Raman mapping, selective etching response, conductive-AFM when available, XPS, TEM/EDS/EELS, illuminated/dark J-V	Distinguishes improved conductivity from harmful conductive secondary phases that create leakage paths.	[25,26,28]
Insulating ZnSe, Sn-Se phases, or buried blocking layers	Suppressed EQE in affected spectral ranges; S-shaped J-V; poor carrier collection; composition-dependent voltage/current trade-off	Raman mapping, XPS/SIMS depth profiles, TEM/EDS/EELS, bias-dependent EQE, temperature-dependent J-V	Identifies blocking barriers and compositionally localized phases that may be invisible in XRD.	[28,37,38]
Nonuniform chalcogenization, Sn loss, S/Se or dopant gradients	Band-gap gradients inconsistent with intended design; spatially variable PL/EQE; enhanced VOC scatter across devices	SIMS or GDOES depth profiles, XPS depth profiling, cross-sectional Raman/PL mapping, statistical J-V/EQE	Links furnace and vapor-transport conditions to depth-dependent defect formation and reproducibility.	[24,32,35]

**Table 25:** High-impact future directions for colloidal CIGS and CZTSSe processing.

Direction	Key scientific Question	Recommended Tools	Expected Impact	Refs.
Operando characterization	What transient phases and vapor species control absorber formation?	In situ XRD, Raman, MS, synchrotron profiling, real-time TEM	Clarifies Se/Sn transport, liquid phases, and reaction pathways	[24,32]
Interface passivation	How can recombination at CdS/CZTSSe and CZTSSe/Mo be reduced?	XPS/UPS, PL/TRPL, temperature-dependent J–V, TEM, SIMS	Reduces Voc deficit and improves carrier collection	[29,30,31,39]
Alloying and defect control	Which additives reduce disorder without creating new losses?	PL, admittance, SIMS, APT/STEM, Hall, EQE	Links Ag/Ge/Sb/Li/alkali effects to Voc and recombination	[47,51,52,53,54]
Scalable coating	Can ink formulation and drying be controlled over large areas?	Rheology, slot-die coating, roll-to-roll drying, coating-defect mapping	Enables manufacturable non-vacuum absorber fabrication	[18,21,22]
Safer processing	Can toxic solvents and etchants be replaced?	Cyanide-free etching, hydrazine-free inks, waste analysis	Improves environmental and industrial viability	[15,43,44]

**Table 26:** Indicative module cost drivers and EPBT trends for representative photovoltaic technologies. The cost shares are approximate literature-based ranges and should be interpreted as technology- and manufacturing-dependent trends rather than universal values.

PV Technology	Indicative Cost Drivers or Component Share	EPBT Trend	Implication for CIGS/CZTSSe Comparison	Refs.
Crystalline Si	Wafer/cell stack and metallization can account for approximately 35–55% of module-related cost; glass, encapsulant, frame, junction box, and backsheet can account for approximately 25–40%; process, capital, yield, and balance-of-system terms form the remainder.	Typically about 1–3 years, depending on irradiation, module efficiency, lifetime, and manufacturing electricity mix.	High efficiency, high yield, and long operational lifetime reduce area-related cost and EPBT, even though wafer production is energy-intensive.	[59,60,61,62]
CdTe	Glass, encapsulation, TCO, contacts, and module materials can account for approximately 45–65%; the thin semiconductor absorber stack is commonly a minor fraction, often below 10–15%; deposition, capital, and yield form the remainder.	Often among the shortest commercial PV EPBT values, approximately sub-year to about 1.5 years under favorable assumptions.	Demonstrates that thin-film PV can be energetically attractive when high-throughput processing, stable efficiency, and high manufacturing yield are achieved.	[59,60,61,62]
CIGS	Substrate or glass and encapsulation may account for approximately 30–45%; TCO, Mo, buffer/window layers, and metal contacts approximately 15–30%; absorber precursors, scribing, process energy, yield, and capital approximately 25–45%.	Generally comparable to other thin-film PV, often about 1–2 years depending on module design, location, and manufacturing assumptions.	The economic issue is not only In/Ga cost, but whether composition control, alkali management, interface quality, and yield can maintain high watt-per-area output.	[57,58,59,60,61,62]
CZTSSe	The Cu/Zn/Sn/S/Se absorber precursor cost is expected to be small, often below approximately 5–10% of module cost; substrate or glass, encapsulation, TCO/Mo/contact stack, process control, yield, capital cost, and lifetime dominate.	No mature commercial-module consensus exists. If efficiency and stability approach CIGS, EPBT should be thin-film-like; at present, lower efficiency increases area-normalized embodied energy per watt.	The cost argument is strongest only if VOC deficit, secondary phases, MoSe_2_/contact control, carrier collection, yield, and stability are improved at scalable module level.	[57,58,59]
Perovskite and tandem PV	Active-layer material share is projected to be small, commonly below 5–10%; encapsulation, electrodes/TCO, substrate, tandem interconnection, yield, lifetime, and replacement dominate practical cost.	Projected to be short, but strongly sensitive to operational lifetime, degradation rate, encapsulation, and replacement assumptions.	Provides a competing low-temperature route, but low process energy is meaningful only if long-term operational stability is demonstrated.	[59,61,62]

## Data Availability

No new data were created or analyzed in this study. Data sharing is not applicable to this article.
